# Synthesis and Characterization of Homo- and Heteroleptic Neptunium(IV) Heteroarylalkenolate Complexes

**DOI:** 10.1021/acs.inorgchem.4c04521

**Published:** 2025-01-27

**Authors:** Dennis Grödler, Peter Kaden, Joseph M. Sperling, Brian M. Rotermund, Benjamin Scheibe, Nicholas B. Beck, Andreas Lichtenberg, Thomas E. Albrecht, Sanjay Mathur, Robert Gericke

**Affiliations:** 1 Institute of Resource Ecology, Helmholtz-Zentrum Dresden-Rossendorf, Dresden 01328, Germany; 2 Department of Chemistry and Nuclear Science & Engineering Center, Colorado School of Mines, Golden, Colorado 80401, United States; 3 Institute of Inorganic and Materials Chemistry, Department of Chemistry, University of Cologne, Greinstr. 6, Cologne 50939, Germany

## Abstract

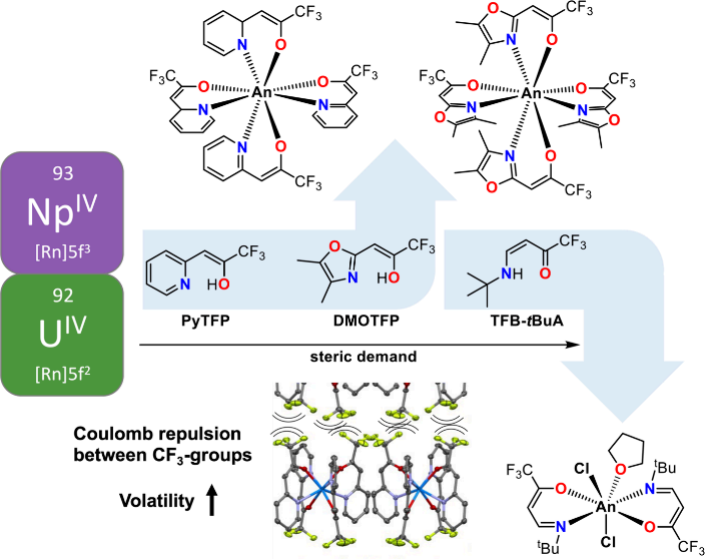

Heteroleptic An^IV^ (An = U, Np) chlorido-ketoenaminate complexes of the type [AnCl_2_(TFB-*t*BuA)_2_(THF)] (**An-1** type: **U-1**, **Np-1**; TFB-*t*BuA = 4-(*tert*-butylamino)-1,1,1-trifluorobut-3-en-2-one) and the homoleptic Np^IV^ heteroarylalkenolate complexes [Np(PyTFP)_4_] (**Np-2**, PyTFP = 1-(pyridin-2-yl)-3,3,3-trifluoroprop-1-en-2-ol) and [Np(DMOTFP)_4_] (**Np-3**, DMOTFP = 1-(4,5-dimethyloxazol-2-yl)-3,3,3-trifluoroprop-1-en-2-ol) were synthesized and characterized (SC-XRD, NMR, Vis-NIR, MS). While their solid-state structures compare well to those of their uranium analogues, the behavior in solution showed significant differences. The binding motif of the DMOTFP ligand in complex **Np-3** can change to form two different complex isomers, as seen by paramagnetic chemical shifts in NMR experiments. Furthermore, the flexibility and the influence of the steric effects at the *N*-side of the ligands are discussed and compared with its uranium counterpart.

## Abstract 2

Heteroalkenolates are suitable ligands to synthesize air-stable homoleptic Np^IV^ complexes in good yield. The Np^IV^ complexes show structural similarity to their U^IV^ homologues, suggesting similar properties as a precursor for nanomaterials shown by ASAP-APCI-MS at low temperatures.

## Introduction

Investigations on volatile actinide complexes have a long story back to the Manhattan Project, in which researchers searched for an efficient way to separate isotopes. Uranium complexes containing fluorides, acetylacetonates, or alkoxides are known to be volatile and have been studied to enrich ^235^U using gas centrifugation.^1−8^ This chemistry was revisited for laser-induced isotope separation using U^VI^ hexamethoxides in 1982.^9,10^ However, the use of UF_6_ has become established and is still the standard compound for uranium enrichment to this day. Since then, interest in volatile actinide compounds has shifted to materials science. The gas-phase deposition of thorium oxide and uranium oxide films was first demonstrated by Shiokawa et al. using actinide containing β-diketonate complexes in 1991.^11^ Volatile and air-stable U^IV^ heteroarylalkenolate complexes have shown to be applicable in chemical vapor deposition (CVD) processes, yielding uranium oxide thin films.^12^ Furthermore, it was reported that U^IV^ amidate complexes have the appropriate properties to produce phase-pure UO_2_ thin films via CVD.^13^ These phase-pure UO_2_ films can serve as a model system for bulk UO_2_ fuel pellets to investigate grain boundaries and changes of the material under irradiation.^14^ Also, the CVD of phase-pure thorium dioxide thin films from highly volatile Th^IV^ alkoxo-enaminonate complexes has recently been demonstrated.^15^

Thin films of neptunium oxides could be advantageous for the in-depth fundamental investigation of neptunium’s physiochemical properties to any great extent. Neptunium complexes, which are suitable as molecular precursors for gas-phase depositions, are scarce. Typically, metal-organic precursors should be sufficiently volatile for an intact transport in the gas phase at low pressures without showing any pronounced tendency for premature decomposition during the materials synthesis.^16,17^ At elevated substrate temperatures, a defined decomposition pathway with preferably volatile byproducts is preferred to ensure direct conversion of the molecular compounds in the desired solid-state phase. Only in 2017, Johnson et al. demonstrated that methylated and/or fluorinated β-diketonates of Np^IV^ show high volatility and are reasonable for further investigations for isotope separation.^18^ The motivation behind this study was to find volatile neptunium compounds that can replace NpF_6_ in the electromagnetic isotope separation, as the corrosive NpF_6_ attacks the internal parts of the instruments. After separation to known ratios of Np isotopes, reference materials can be produced that are needed in nuclear forensic analyses.^18^ In order to synthesize suitable Np^IV^ complexes for gas-phase deposition, we decided to revisit bidentate heteroarylalkenolate and β-ketoiminate ligands that have already proven to stabilize U^IV^ in homoleptic complexes.^12,19^

**Chart 1:**
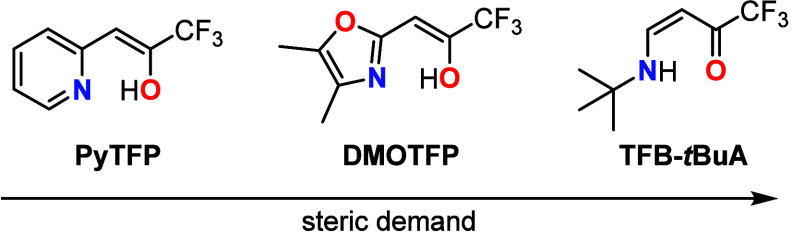
Lewis Structures of Bidentate *N,O*-Ligands Discussed in this Manuscript

Herein, we report the synthesis and characterization of three Np^IV^ complexes with the bidentate *N*,*O*-ligands 4-(*tert*-butylamino)-1,1,1-trifluorobut-3-en-2-one (TFB-*t*BuA, **1**), 1-(pyridin-2-yl)-3,3,3-trifluoroprop-1-en-2-ol (PyTFP, **2**), and 1-(4,5-dimethyloxazol-2-yl)-3,3,3-trifluoroprop-1-en-2-ol (DMOTFP, **3**) (Chart 1). These ligands differ mainly in their conjugated system and the steric demand of the substituents at the *N*-side to increase volatility by surpassing intermolecular interactions. The insertion of CF_3_ groups on the other side has proven useful to also increase the volatility of the resulting complexes, due to the electrostatic *Coulomb* repulsion.^20^ The customizability of these ligands led to isolation of a wide range of transition-, main group-, and *f*-block metal complexes.^12,19−25^ By adjusting the steric demand of the ligand to the ionic radii of the metal centers, homoleptic and heteroleptic complexes could be isolated that show different suitability as precursors for CVD. In addition, ^19^F is a very sensitive nucleus for NMR spectroscopic investigations and can be used to provide useful information about speciation in solutions.

## Results and Discussion

The sterically demanding TFB-*t*BuA (4-(*tert*-butylamino)-1,1,1-trifluorobut-3-en-2-one, **1**) ligand has not been studied in the coordination chemistry of U^IV^. However, the use of PyTFP (1-(pyridin-2-yl)-3,3,3-trifluoroprop-1-en-2-ol, **2**) and DMOTFP (1-(4,5-dimethyloxazol-2-yl)-3,3,3-trifluoroprop-1-en-2-ol, **3**) as bidentate ligands led to the known complexes [U(PyTFP)_4_] (**U-2**) and [U(DMOTFP)_4_] (**U-3**).^12,19^

Attempts to synthesize complexes of the form [An(TFB-*t*BuA)_4_] (An = U, Np), UCl_4_, or [NpCl_4_(DME)_2_] with four equivalents of K-TFB-*t*BuA (**K-1**) did not lead to the formation of the targeted products. Titrations of UCl_4_ with one, two, and three equivalents of **K-1** in THF-*d*_8_ (THF = tetrahydrofuran) were monitored with ^1^H NMR experiments to determine the limitations of this ligand system (Figure S5). Significant signal broadening in the ^1^H NMR spectrum takes place when more than two equivalents of **K-1** were added to the actinide chloride. The reaction of either one or two equiv of **K-1** with UCl_4_ in THF-*d*_8_ leads to a green suspension and precipitation of KCl. After separation of KCl, the ^1^H NMR spectra show the same one set of ligand signals, which points to the presence of one species in the bulk and time average of this method. The signals can be assigned to isolated structure **U-1**, although other isomers and conformers are conceivable in THF solutions. Due to the paramagnetic character and fast chemical exchange, the signals were too broad (full width at half-maximum: 210 Hz at 298 K) for 2D NMR experiments and it is only possible to distinguish the vinylic protons from the methyl group by comparing the integrative ratio. Therefore, the vinylic protons are found at 47.89 and 35.87 ppm and the methyl groups are strongly shielded to −42.77 ppm. Traces of the protonated ligand are found at 9.12 and 6.73 ppm due to incomplete deprotonation of ligand **1** (Figure S5). After addition of one more equivalent of ligand to the solution, the resonances did not shift but show peak broadening especially for the signal at −42.77 ppm. These broadening effects develop drastically with further addition of **K-1**, where the vinylic protons also show strong signal broadening (Figure S5). A possible explanation for this effect is a faster chemical exchange rate of the ligands that leads to an exchange broadening in the spectrum. VT measurements did not result in sharper signals but enabled the identification of other conformers at low temperatures (Figure S6). At 218 K, seven other signals can be found for ^19^F in a range from 20.3 to −100.4 ppm (Figure S7), while the main signal from F-1 is significantly broadened to 330 Hz and shifted to −12.96 ppm (in comp.: −33.99 ppm, 73 Hz at 298 K). This indicates a very dynamic behavior in solution, possibly due to THF coordination and the steric influence of the bulky N-*t*Bu group.

In an attempt to synthesize **Np-1**, the ^1^H NMR spectrum of the crude reaction mixture showed a very similar behavior in THF, as observed for **U-1**. The signal at −39.30 ppm, which was assigned to the N-*t*Bu group, is already broadened to 285 Hz at room temperature (Figure S8). The vinylic protons are found at 37.11 and 44.98 ppm, respectively. The ^19^F NMR spectrum indicates decomposition of the ligand, as several diamagnetic resonances are found with low intensity between 74.2 and 75.5 ppm (Figure S9). However, the signal at −14.90 ppm was assigned to F-1 of possible compound **Np-1**. At low temperatures, the signal for F-1 drastically broadens and shifts to 20.72 ppm. The NMR results are in alignment with a putative **Np-1** as the main product, but due to signal broadening and the presence of additional signals, further isomers or byproducts cannot be excluded.

**Scheme 1:**
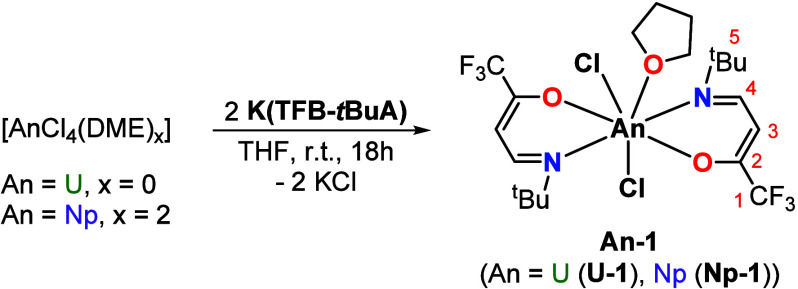
Synthesis of [UCl_2_(TFB-*t*BuA)_2_(THF)] (**U-1**) and [NpCl_2_(TFB-*t*BuA)_2_(THF)] (**Np-1**) Other isomers and conformers cannot be completely excluded due to the dynamic behavior in solution.

**Figure 1:**
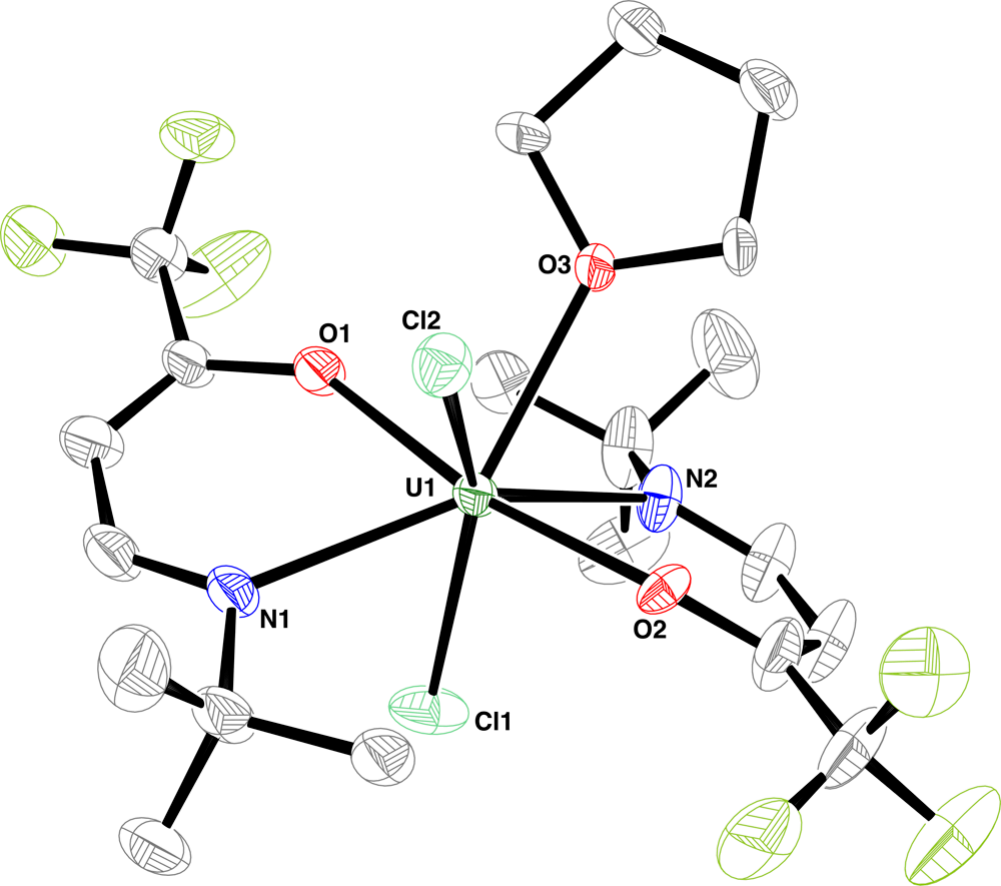
Molecular structure of **U-1**. Hydrogen atoms have been omitted for the sake of clarity. All ellipsoids are drawn at the 30% probability level. The ligand at N2 and the THF position at O3 are disordered in 71.2(4)%/28.8(4)%. Selected bond lengths (Å): U1—O1 2.158(4), U1—O2 2.193(13), U1—O3 2.473(5), U1—N1 2.587(5), U1—N2 2.612(8), U1—Cl1 2.655(1), and U1—Cl2 2.666(1).

Single crystals of [UCl_2_(TFB-*t*BuA)_2_(THF)] (**U-1**, Figure 1) could be grown from saturated THF solutions after workup of reaction mixtures of UCl_4_ and two equivalents of **K-1** (Scheme 1). **U-1** crystallizes in the orthorhombic space group *Pccn*, where the uranium center is 7-fold coordinated, resulting in a distorted pentagonal bipyramid (O1—U1—O2 169.7(4)° axial, Cl1, Cl2, N1, N2, and O3 equatorial plane). This unusual geometry can possibly be attributed to the increased size of the N-*t*Bu substituent, whereby only two ligands can coordinate at most under the chosen conditions. Average bond lengths between the uranium center and oxygen are found at U–O 2.175(23) Å and to nitrogen at U–N 2.600(16) Å. The bonds between uranium and chlorine (avg. U–Cl 2.660(6) Å) seem to be unaffected by the coordination of two bidentate ligands since those lengths are common for UCl_4_ and its adducts.^26−28^ It should be mentioned that compound **U-1** is rather unstable compared to the heteroalkenolate complexes (Figure S5).^12^ The crystallization of **U-1** required several attempts since visual decomposition was observed during the drying of the compound. The stability of this complex type seemed to further decrease when attempts were made to crystallize [NpCl_2_(TFB-*t*BuA)_2_(THF)] (**Np-1**). Until now, it has not been possible to isolate single crystals of this material suitable for single-crystal X-ray diffraction analysis.

**Figure 2:**
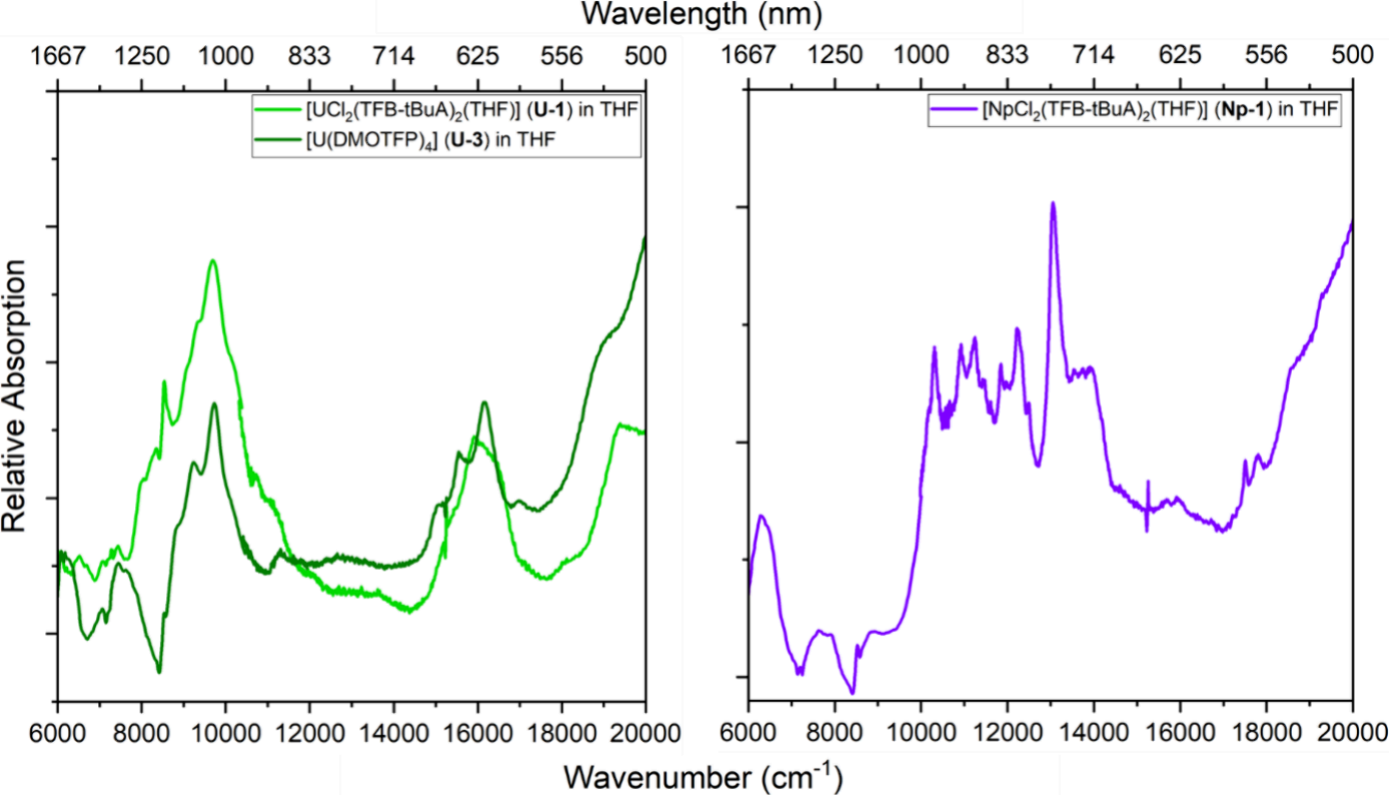
Vis-NIR spectra of [UCl_2_(TFB-*t*BuA)_2_(THF)] (**U-1**, chartreuse) and [U(DMOTPF)_4_] (**U-3**, dark green) in THF solution of **U-1** (left). Vis-NIR spectra of [NpCl_2_(TFB-*t*BuA)_2_(THF)] (**Np-1**) in a THF solution (right).

Figure 2 shows the optical spectra of **U-1** and **U-3** in the THF solutions. Due to different possible isomers of **U-1** in THF solutions, the extinction coefficients could not be calculated with sufficient certainty. However, comparisons of the band positions between **U-1** and **U-3** are drawn using relative absorption. In both compounds, two broad bands are observed in the visible region at 16,100 cm^–1^ (621 nm) and in the near-infrared region at 9670 cm^–1^ (1034 nm), while **U-3** is slightly shifted to 9730 cm^–1^ (1028 nm) at this position. These band positions are consistent with reported absorption spectra of U^IV^ compounds in the literature.^29−32^ However, assignment of electronic transitions and interpretation of such is particularly difficult for U^IV^ due to the larger crystal-field splitting and relatively strong vibronic lines.^29,31,33^ In addition, the collected Vis-NIR spectrum of **Np-1** shows a similar dense structure of 5f → 5f transitions between 10,000 cm^–1^ (1000 nm) and 16,000 cm^–1^ (625 nm) as **Np-2** and **Np-3**, confirming the oxidation state to be +IV (*vide infra*).^29^

**Scheme 2:**
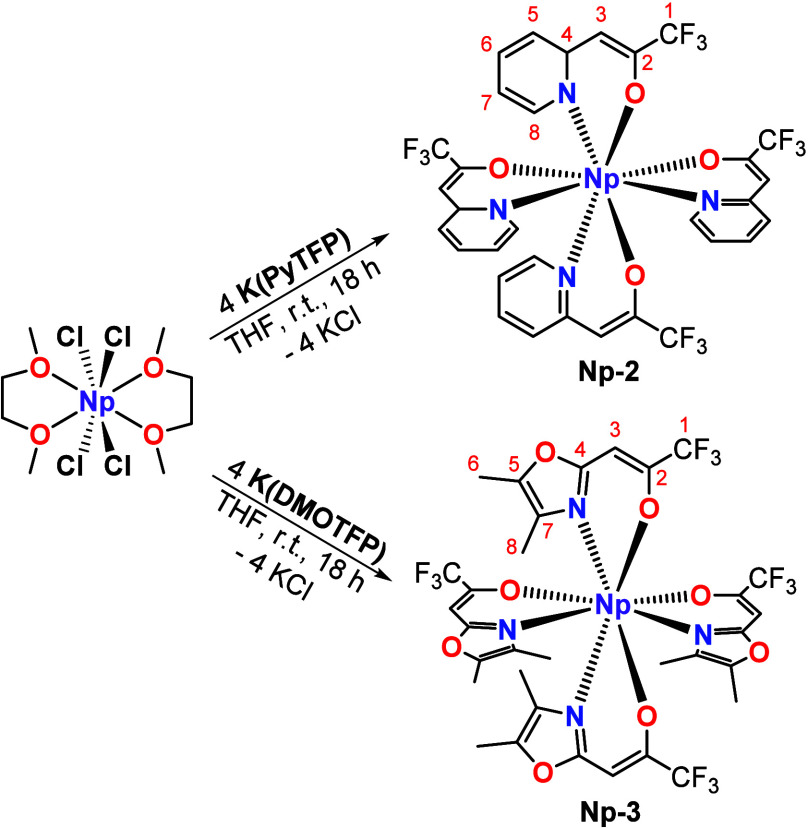
Synthesis of [Np(PyTFP)_4_] (**Np-2**) and [Np(DMOTFP)_4_] (**Np-3**)

Heteroarylalkenolate complexes of the type [Np^IV^(L)_4_] (L = PyTFP^–^, DMOTFP^–^) were synthesized via the salt metathesis reaction of [NpCl_4_(DME)_2_] with the potassium salts K(PyTFP) (**K-2**) and K(DMOTFP) (**K-3**) (Scheme 2). Ligands **2** and **3** can be deprotonated with either KO*t*Bu or K[N(SiMe_3_)_2_], whereby K[N(SiMe_3_)_2_] can directly be added to a solution of [NpCl_4_(DME)_2_] and **2** or **3** in THF. Both methods resulted in similar yield (>80%).

**Figure 3:**
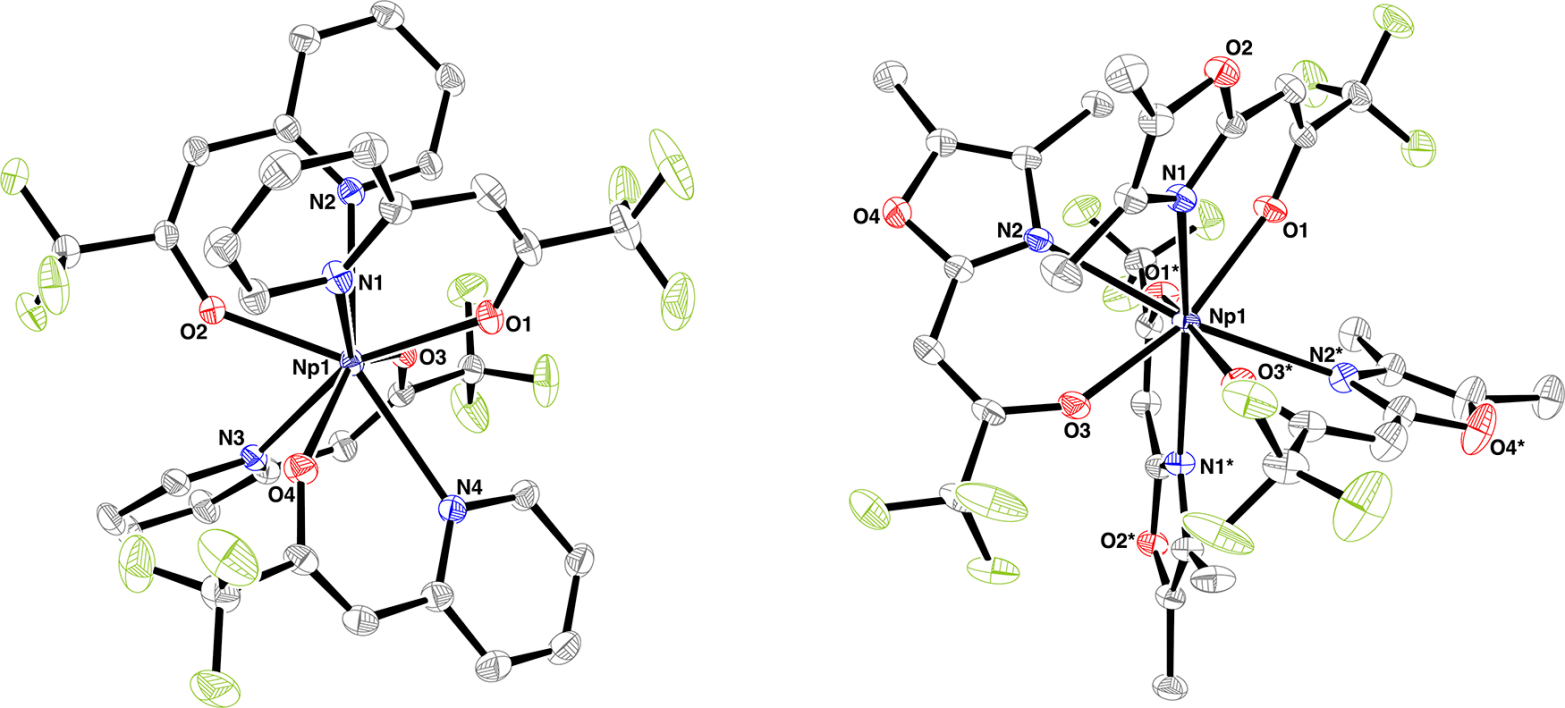
Molecular structure of **Np-2** (left) and **Np-3** (right). Hydrogen atoms are omitted for clarity, and ellipsoids are drawn at the 50% probability level. The asymmetric unit of **Np-3** comprises one-half molecule of **Np-3** (its atom Np1 is located on a crystallographic twofold rotation axis). Symmetry equivalent atom labels are asterisked. Selected bond lengths (Å): **Np-2**: Np1—O1 2.225(2), Np1—O2 2.211(2), Np1—O3 2.222(2), Np1—O4 2.212(2), Np1—N1 2.612(2), Np1—N2 2.642(2); Np1—N3 2.668(2), Np1—N4 2.670(2); **Np-3**: Np1—O1 2.241(1), Np1—O2 2.239(4), Np1—N1 2.605(1), and Np1—N2 2.638(1).

Bright yellow single crystals of **Np-2** suitable for single-crystal X-ray diffraction analysis were grown by the slow evaporation of the solvent at ambient temperature (Figure S1). **Np-2** crystallizes in the orthorhombic space group *Pbca*, and the neptunium atom is surrounded by four PyTFP (**2**) ligands in a distorted square antiprismatic fashion (Figure 3). **Np-2** is isostructural to its U^IV^ analogue reported by Appel et al. in 2015.^12^ The mean Np–O (2.218(7) Å) and Np–N (2.648(25) Å) distances are slightly shorter than those of the reported U–O (2.226(8) Å) and U–N (2.666(27) Å), respectively, due to the smaller ionic radius of Np^IV^ (U^IV^: 1.00 Å, Np^IV^: 0.98 Å).^34^ The endogenous CF_3_ groups lead to an overall connectivity with separated layers running along the crystallographic *a*- and *c*-axes, which can be seen in Figure S2. Since these layers are only connected through *van der Waals* forces between the fluorine atoms, the crystals grow predominantly in two directions and are isolated as yellow plates and thin rods. Even though the structure does not show extended connectivity between the Np^IV^ sites, the compound shows marked air stability. Once the compound is isolated in the solid state, it can be stored at ambient conditions.

Suitable single crystals of **Np-3** for single-crystal X-ray diffraction analysis were isolated from concentrated THF solutions at ambient temperature (Figure S3). **Np-3** crystallizes in the monoclinic space group *C*2/*c*, and the molecular structure can be seen in Figure 3. The Np^IV^ coordination is best described as a distorted cube with four DMOTFP (**3**) ligands. This derivative of heteroarylalkenolate was also used to coordinate Ce^IV^ and U^IV^ in isostructural complexes to synthesize the volatile precursor for CeO_2_ and UO_*x*_ nanomaterials.^12,19,21^ The mean Np–O (2.240(2) Å) and Np–N (2.621(18) Å) distances are slightly shorter than the reported U–O (2.246(8) Å) and U–N (2.652(21) Å), respectively, due to the smaller ionic radius of Np^IV^. The angles of the opposite oxygen O–Np–O (138.2(6)°) and nitrogen N–Np–N (140.4(22)°) atoms in complex **Np-2** are more acute than in complex **Np-3** (O–Np–O: 162.6(1)°; N–Np–N: 164.6(1)°), making **Np-3** the species with a more distorted square antiprism. In fact, a continuous shape measures (CShM) analysis showed that **Np-3** deviates less from a cube (CShM: **Np-3**: 2.580; **Np-2**: 9.281) or triakis tetrahedral (CShM: **Np-3**: 3.508; **Np-2**: 8.699) coordination than from a square antiprism (CShM: **Np-3**: 4.638; **Np-2**: 1.283).^35^ Due to the repulsive interactions of the bulky methyl groups in **Np-3**, the trans-laying ligands are also more twisted by 49.6(16)° compared to **Np-2** with approximately 21.3(17)°. In addition, the repulsive interactions of the CF_3_ groups lead to a twisting of the originally planar ligands. Detailed bond lengths and angles are listed in Table 1.

**Table 1:** Mean Bond Lengths (Å) and Mean Angles (°) of Complex **Np-2**, **U-2**,^12^**Np-3**, and **U-3**

	Np-2	U-2	Np-3	U-3
An—O	2.218(7)	2.226(8)	2.240(2)	2.246(8)
An—N	2.648(25)	2.666(27)	2.621(19)	2.652(21)
O—An—O	138.2(6)	138.1(7)	162.6(1)	163.3(1)
N—An—N	140.4(22)	140.5(23)	164.6(1)	166.2(1)
L—An—L^a^	21.3(17)	21.3(7)	49.6(16)	48.5(6)

a The angle L-An-L describes the angle between the planes of two opposing ligands (e.g., in **Np-2**, ligand at N1 and N3). The plane of a ligand was constructed including all carbon, nitrogen, and oxygen atoms.

NMR spectra of complex **Np-2** (Figures S10–S18) were collected via 1D and 2D NMR spectroscopy in solution, and all signals could be unambiguously assigned. Due to the paramagnetic character of the Np^IV^ center, signals are shifted characteristically. At 293 K, the ^1^H NMR spectrum (Figure S17) shows one set of five broad signals at 13.74 (H-8), 7.65 (H-6), 6.73 (H-7), 5.75 (H-5), and 2.10 ppm (H-3), which drastically sharpen and shift to 12.86 (H-8), 7.94 (H-6), 6.52 (H-7), 5.79 (H-5), and 0.96 ppm (H-3) when the sample was cooled to 233 K. Only one signal can be found in the ^19^F NMR spectrum (Figure S15) at −68.5 ppm (233 K), confirming that the complex exhibits a *D*_*2*_ symmetry in THF solution in the time and bulk average of the method.

Interestingly, two sets of signals can be found in ^1^H, ^13^C, and ^19^F NMR spectra of **Np-3** at 223 K (sets: **Np-3**, **Np-3′**; Figures S19–S31). By comparing the integrals in the ^19^F spectrum (Figure S27), the two signal sets were found to be in an approximate 70%/30% (**Np-3/Np-3′**) ratio. Despite the paramagnetic character of both species, the signals were sufficiently sharp for 2D experiments at 233 K. All of the ^1^H, ^13^C, and ^19^F NMR signals could be unambiguously assigned. The methyl groups are strongly shielded to −10.07 ppm (H-8) and −2.20 ppm (H-6) for **Np-3** and −3.09 ppm (H-8) and 2.52 ppm (H-6) for **Np-3′**. The vinylic proton (H-3) is found at 7.44 ppm (**Np-3**) and 5.85 ppm (**Np-3′**). The ^1^H,^1^H-NOESY experiment (Figure S31) has shown an exchange signal between **Np-3** and **Np-3′** at the H-6/H-6′ position. Dissolution of the crystalline material of **Np-3** results in the two signal sets in NMR spectroscopy. Recrystallization of **Np-3** from the solution used for NMR experiments gave only single crystals of the composition, as presented in Figure 3. In conclusion, it appears that **Np-3** undergoes some kind of isomerization in a THF solution, which is in agreement with the NMR signal broadening observed at ambient temperature (Figure S29). Given that only one other set with three signals in the ^1^H NMR spectrum is observed, it appears that **Np-3′** also possesses *D*_*2*_ or *C*_*4*_ symmetry in solution, which means that only one other isomer with all CF_3_ groups is facing in one direction (Scheme S1) is conceivable. Calculations at the DFT level of theory have shown that the relative energy difference between these two isomers is about 18.1 kcal/mol, with **Np-3** being lower in energy (Figures S53 and S54 and Tables S3 and S4). As **Np-3′** is only slightly higher in energy, it appears feasible that both isomers can coexist in solution at ambient temperature. However, we have not been able to isolate another isomer in the solid state and could only observe this phenomenon in solution. NMR experiments in DCM-*d*_2_ gave the same two sets of signals in the same integrative ratio (Figure S32), clearly demonstrating that THF coordination does not take place.

In contrast, compound [U(DMOTFP)_4_] (**U-3**) was reported to give only one set in the ^1^H, ^13^C, and ^19^F NMR spectra at ambient temperature.^12^ The proton signals were found at 11.79 ppm (H-3), −1.96 ppm (H-6), and −14.16 ppm (H-8), while ^19^F was found at −67.0 ppm. After reinvestigation of **U-3** at 218 K, these signals shift to 14.46 (H-3), −3.17 (H-6), and −22.07 ppm (H-8), while ^19^F is shifted to −57.68 (F-1) and a second signal set at 15.50, −2.27, and −19.76 ppm is visible at about 4% (Figures S33–S41). At lower temperatures, isomerization is thermally hindered, which makes this observation to accord well with the NMR results found for complex **Np-3**.

**Figure 4:**
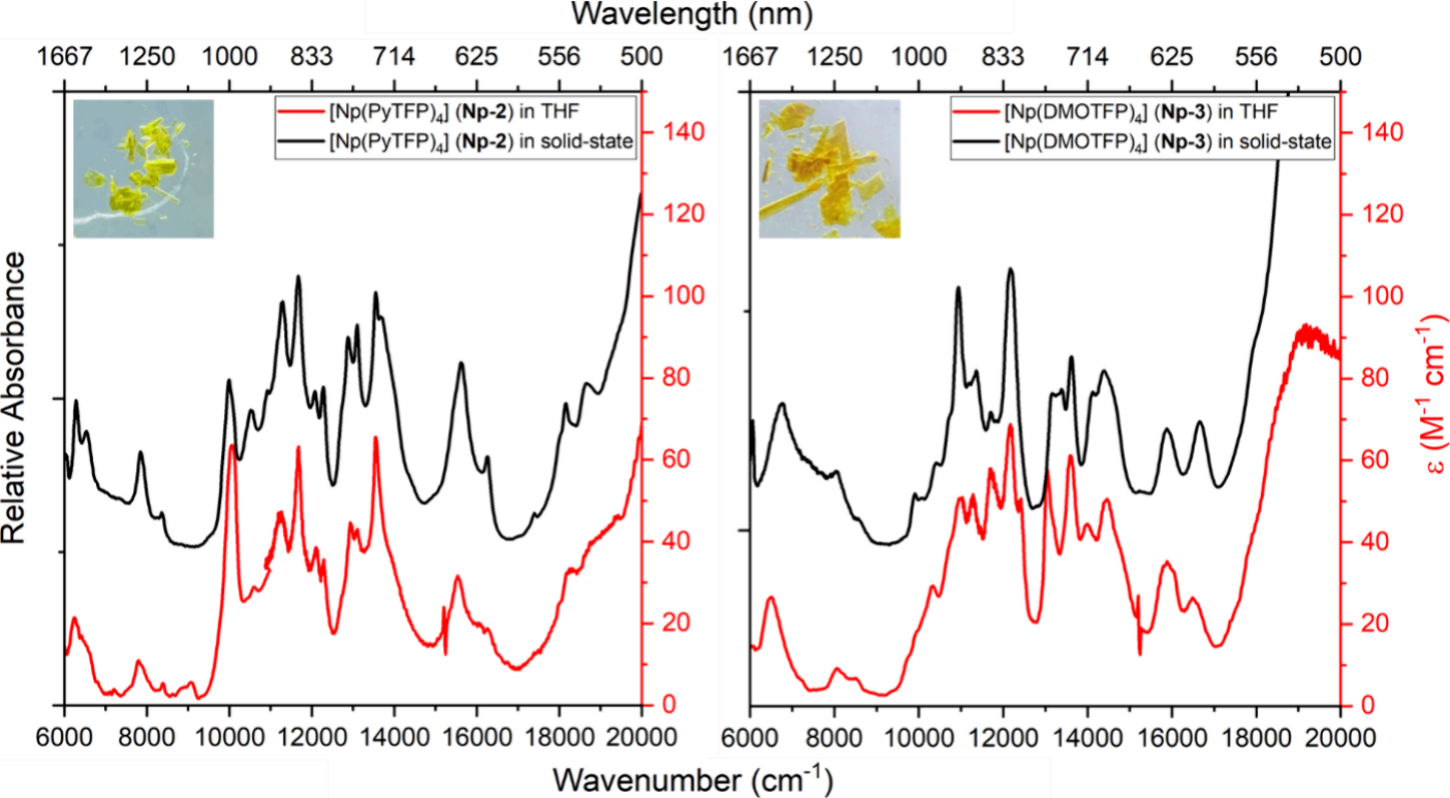
Vis-NIR spectra at ambient temperature of [Np(PyTFP)_4_] (**Np-2**, left) in the solid state (black line) and in THF solution (7.5 mM, red line) and [Np(DMOTFP)_4_] (**Np-3**, right) in the solid state (black line) and in THF solution (13.4 mM, red line) with a photograph of each compound.

Optical absorption spectra of **Np-2** and **Np-3** were collected at room temperature in the solid state and in THF solutions (Figure 4). The 5f → 5f transitions observed between 6000 and 20,000 cm^–1^ are consistent with the electronic profile that is reported in many Np^IV^-containing structures.^29,36,37^ Interestingly, the characteristic dense structure between 10,000 and 16,000 cm^–1^ is very pronounced and shows quite sharp 5f → 5f transitions. Due to the huge overlap and mixing of the higher-lying transition states, the transitions cannot be assigned unambiguously to their Russel Saunders term symbols, which is why the following mainly discusses the positions and relative intensities in order to analyze differences between the ligand systems and between the solid state and structure in solution.^29^ In general, the peak positions, splitting, and shape of **Np-2** in the solid state and solution are comparable. A few transitions at, e.g., 11,682 and 13,550 cm^–1^, have a similar intensity ratio in solution (1.04) and in the solid state (*I*_13,541cm–1_/*I*_11,669cm–1_ = 0.98). However, the relative intensity between the signals at 12,933 and 13,550 cm^–1^ is ∼1.47 in solution and the corresponding ratio in the solid state is ∼1.06 (*I*_13,541cm–1_/*I*_12,875cm–1_). We assign this change in relative intensities to a slight change in the ligand field of Np^IV^ in **Np-2** with respect to the solid-state structure due to the absence of packing effects in solution. The solution absorption spectrum of **Np-3** shows not only differences in relative intensity in comparison to the solid-state spectrum but also small bathochromic shifts of, e.g., the peak at 16,670 cm^–1^ (solid) to 16,480 cm^–1^ (solution) and from 13,200 cm^–1^ (solid) to 13,050 cm^–1^ (solution). This suggests pronounced fluxional behavior in solution in comparison to **Np-2** (Scheme S1). These findings are in accordance with those of the NMR spectra discussed earlier.

To evaluate the suitability of the homoleptic complexes **Np-2** and **Np-3** for CVD, a series of ASAP-APCI-MS (ASAP-APCI = atmospheric solid analysis probe – atmospheric-pressure chemical ionization) analyses were conducted at different temperatures (Figures S42–S51). With this method, solid **Np-2** or **Np-3** was placed between the heated nitrogen gas flow outlet and the corona discharge needle. The nitrogen gas will evaporate the sample, which will be ionized later at the corona discharge needle. By incremental heating of the nitrogen gas, an indication of the volatility of the samples can be concluded. In direct comparison, **Np-3** demonstrated higher volatility as indicated by the increased presence of [Np(DMOTFP)_3_]^+^ (*m*/*z* 855.2) in the mass spectra at 125 °C. Conversely, **Np-2** began to show [Np(PyTFP)_3_]^+^ (*m*/*z* 801.2) only at 175 °C. In addition, a second fragment [Np(DMOTFP)_4_-F]^+^ (*m*/*z* 1042.2) became detectable at 200 °C. A similar fragment for **Np-2** [Np(PyTFP)_4_-F]^+^ (*m*/*z* 970.2) was observed at 250 °C. Above 300 °C, both complexes mainly fragmented through the CF_3_ group, suggesting that much higher substrate temperatures would be necessary during a CVD process in order to deposit neptunium oxide layers. This would be in agreement with similar Th^IV^ or U^IV^ complexes, where substrate temperatures in the range of 500 to 700 °C were used during CVD.^12,13,15^ Given that ASAP-APCI-MS operates at atmospheric pressure, the observed fragmentation pattern and volatility differences suggest that **Np-3** is the most suitable candidate for CVD, with the assumption that its volatility would be even higher under the reduced pressure conditions typically used in CVD processes.

## Conclusions

In summary, we have shown that heteroalkenolates are suitable ligands to synthesize air-stable homoleptic Np^IV^ complexes **Np-2** and **Np-3** in good yield. These complexes were analyzed in the solid state and in THF solution. We found that they show structural similarity to their U^IV^ homologues suggesting similar properties as a precursor for nanomaterials, also shown by the presence in ASAP-APCI-MS at low temperatures. In solution, however, **Np-2** shows some kind of isomerization, which was not observable for the uranium homologue to such extent. This shows that despite the similar ionic radii and oxidation state of U^IV^ and Np^IV^, the binding affinity between N- and O-donor atoms can change along the actinide series. The steric demand of the ligand was then further increased by substituting a *tert*-butyl group at the *N*-side. It was shown that under chosen conditions, only two chlorides could be substituted from the actinide center, resulting in heteroleptic complexes **U-1** and possibly **Np-1**. These complexes showed a dynamic behavior in solution, due to fast exchange between the ligand and THF molecules. These complexes are potentially not suitable for CVD due to their low volatility and instability in air. Along the investigated series of ligands, **3** seems to be the most suitable candidate, as it balances steric demand and complex stability with tetravalent actinides.

## Experimental Section

**Caution!** Neptunium-237 (*t*_1/2_ = 2.144 × 10^6^ years; *a* = 2.6 × 10^4^ Bq/mg) and uranium (^234^U, ^235^U, and ^238^U from natural uranium; *a* = 25.4 Bq/mg) are serious internal health hazard due to their α-emissions. All experiments were carried out in radiochemical laboratories at the Helmholtz-Zentrum Dresden-Rossendorf (Germany) or Florida State University (USA), and all handling with radioactive material was choreographed with nonradioactive material and operational procedures were approved by radiation safety prior to any experiments involving radioactive material.

### General Considerations

The reactions were conducted inside a negative pressure nitrogen atmosphere glovebox (MBraun). Glass vials were dried at 150 °C in a drying oven before use. THF (Carl Roth, ≥99.9%, unstabilized) was purified and dried via SPS (MB SPS 5) and stored over 3 Å molecular sieves prior to use. Potassium bis(trimethylsilyl)amide K[N(SiMe_3_)_2_] (Sigma-Aldrich, 95%) was used without further purification. The preparations of the starting materials [NpCl_4_(DME)_2_], UCl_4_, TFB-tBuA (**1**), PyTFP (**2**), DMOTFP (**3**), and [U(DMOTFP)_4_] (**U-3**) were conducted according to previously reported procedures.^12,38−41^ Ligands **1**–**3** were either deprotonated with 1 equiv. KO*t*Bu or 1 equiv. K[N(SiMe_3_)_2_] in THF. All volatiles were removed prior to use. Further experimental details are given in the Supporting Information.

## Supporting Information Available

Instrumentation, experimental details for the preparation of Np^IV^ heteroarylalkenolates, and crystallographic details (PDF)
