# Supplementary material for: Synthesis and Characterization of Homo- and Heteroleptic Neptunium(IV) Heteroarylalkenolate Complexes
Source: Inorg Chem. 2025 Jan 27;64(5):2321–8. doi: 10.1021/acs.inorgchem.4c04521 (PMC11815852; doi:10.1021/acs.inorgchem.4c04521)
Supplement: Supplementary file 1 — ic4c04521_si_001.pdf [file ic4c04521_si_001.pdf]

Supporting Information for

Synthesis and Characterization of Homo- and Heteroleptic Neptunium(IV) Heteroarylalkenolate Complexes

Dennis Grödler,[1] Peter Kaden,[1] Joseph M. Sperling,[2] Brian M. Rotermund,[2] Benjamin Scheibe,[2] Nicholas B. Beck,[2] Andreas Lichtenberg,[3] Thomas E. Albrecht,[2] Sanjay Mathur,[3] Robert Gericke[1*]*

[1]Institute of Resource Ecology, Helmholtz-Zentrum Dresden-Rossendorf, 01328 Dresden, Germany,

*r.gericke@hzdr.de

[2]Department of Chemistry and Nuclear Science & Engineering Center, Colorado School of Mines, Golden, Colorado 80401, United States

[3]Institute of Inorganic and Materials Chemistry, Department of Chemistry, University of Cologne, Greinstr. 6, 50939 Cologne, Germany, *sanjay.mathur@uni-koeln.de

Table of Contents

Instrumentation S2

Single-crystal X-ray diffraction S2

NMR Spectroscopy S2

Solid-State Vis-NIR Absorption Spectroscopy S2

Vis-NIR Absorption Spectroscopy in solution S2

ASAP-APCI Mass Spectroscopy S3

Experimental details S3

Synthesis of [UCl_2(TFB-*t*BuA)$_2$(THF)] (U-1) S3

Synthesis of [$NpCl_2$(TFB-*t*BuA)$_2$(THF)] (Np-1) S4

Synthesis of [Np(PyTFP)$_4$] (Np-2) S4

Synthesis of [Np(DMOTFP)$_4$] (Np-3) S5

NMR Spectra S7

[UCl_2(TFB-*t*BuA)$_2$(THF)] (U-1) S7

[$NpCl_2$(TFB-*t*BuA)$_2$(THF)] (Np-1) S9

[Np(PyTFP)$_4$] (Np-2) S10

[Np(DMOTFP)$_4$] (Np-3) S14

[U(DMOTFP)$_4$] (U-3) S22

APCI-MS Spectra S27

Crystallographic details S31

Quantum Chemical Calculations S33

Atomic coordinates and total energies from DFT calculations: S34

References S39

Instrumentation

Single-crystal X-ray diffraction

Single crystals of the compounds were selected under mineral oil and mounted onto a MiTeGen microloop. Intensity data of suitable crystals of **Np-2** and **Np-3** were recorded with a Bruker D8 Quest diffractometer (Photon III detector) with Mo-K_α radiation (λ = 0.71073 Å, multi-layered optics) at 100 K (Cryocool LN-3, *Cryo Industries*). Single crystals of **U-1** and **U-3** were measured on a Bruker D8 Venture diffractometer (Photon II 7 array detector) and micro focus Mo-K_α radiation (λ = 0.71073 Å) with mirror optics monochromator at 100 K (800 Series Cryostream Cooler, *Oxford Cryosystems*). The computer programs SMART and SAINT, which are implemented in the APEX4 software suite,[1] were used for data collection in φ- and ω-scan modes and data processing, respectively. The absorption correction of diffraction data was performed with the multi-scan method with SADABS.[2] The structures were solved with the dual-space method or intrinsic phasing and refined with full-matrix least-squares methods on F^2 using the SHELX-TL package (SHELXT).[3,4] All non-hydrogen atoms were refined with anisotropic displacement parameters. All hydrogen atoms were positioned geometrically and refined using a riding model with isotropic displacement parameters. CIF files have been deposited with the Cambridge Crystallographic Data Center (CCDC) and can be obtained free of charge (for inquiry contact: CCDC, 12 Union Road, Cambridge, CB2 1EZ, UK, fax: +44−1223−336033, e-mail: deposit@ccdc.cam.ac.uk) quoting the following reference numbers: CCDC-2370142-2370145.

NMR Spectroscopy

NMR spectra were recorded on a Varian MR 400 with a ^{1}H frequency of 401.77 MHz, ^{19}F frequency of 378.00 MHz and a ^{13}C frequency of 101.03 MHz. All spectra were recorded on a Varian OneNMR probe head. Sample solutions were filled in a J. Young tap NMR tubes and were swipe tested before they were put in the NMR spectrometer. ^{1}H and ^{13}C NMR spectra were referenced with the internal standard tetramethylsilane (TMS) or using the residual protonated solvent peaks. Deuterated solvents were purchased from Deutero GmbH, and dried over potassium metal and stored over 3 Å molecular sieve prior to use. MestReNova v14.2 was used to process and plot the recorded spectra.

Solid-State Vis-NIR Absorption Spectroscopy

Isolated crystals under Parabar 10312 immersion oil on a glass slide were placed on the stage of a Craic Technologies 20/20 PV dual microspectrophotometer. 100 W Hg and 75 W Xe lamps were used for transmission measurements, respectively. Integration times were optimized by using the Craic Technologies software.

Vis-NIR Absorption Spectroscopy in solution

Electronic absorption spectra in THF solutions were collected in 1 cm quartz cuvettes using an AIS DT2000 Deuterium-Tungsten Light Source and an Avantes AvaSpec-ULS2048x64-EVO detector

connected with fiber optic cable through the glovebox. AvaSoft 8 software was used to determine integration times, collecting background and dark spectra.

ASAP-APCI Mass Spectroscopy

Atmospheric solids analysis probe - atmospheric-pressure chemical ionization mass spectroscopy was performed on an Advion Interchim Scientific (Montluçon, France) expression-L Compact Mass Spectrometer.

Experimental details

All experiments containing ^{237}Np ($t_{1/2} = 2.144 \cdot 10^6$ a; $a = 2.6 \cdot 10^4$ Bq/mg) and uranium (^{234}U, ^{235}U and ^{238}U from natural uranium; a = 25.4 Bq/mg) were carried out in a certified radiochemical laboratory at the Helmholtz-Zentrum Dresden-Rossendorf (Germany) or Florida State University (USA), due to their high α-emitting activity. The reactions were conducted inside a negative pressure nitrogen atmosphere glovebox (MBraun). Glass vials were dried at 150 °C in a drying oven before use. THF (Carl Roth, ≥99.9%, unstabilized) was purified and dried via SPS (MB SPS 5) and stored over 4 Å molecular sieves prior to use. Potassium bis(trimethylsilyl)amide $K[N(SiMe_3)_2]$ (Sigma Aldrich, 95%) was used without further purification. The preparations of the starting materials $[NpCl_4(DME)_2]$, UCl_4, TFB-tBuA (**1**), PyTFP (**2**), DMOTFP (**3**), and $[U(DMOTFP)_4]$ (**U-3**) were conducted according to previously reported procedures.[5–9] Ligands **1-3** were either deprotonated with 1 equiv. KO^{t}Bu or 1 equiv. $K[N(SiMe_3)_2]$ in THF. All volatiles were removed prior to use.

Synthesis of $[UCl_2$(TFB-*t*BuA)$_2$(THF)] (U-1)

To a solution of 10.0 mg UCl_4 (26.3 µmol, 1.0 equiv.) in 1 mL THF was slowly added a solution of 12.2 mg K-TFB-*t*BuA (**K-1**) (52.6 µmol, 2.0 equiv.) in 1 mL THF. The green solution was stirred for 18 h at room temperature to give a colorless precipitate that was filtered with a 0.2 µm PTFE syringe filter. All volatiles were removed under reduced pressure to give a green solid (m = 14.3 mg). Green single crystals of the material were obtained by slow evaporation of the reaction mixture after filtration at room temperature after 2 days.

^{1}H NMR: (401.7 MHz, THF-d_8, 298 K): δ (ppm) = 47.59 (s_{br}, 2H, $H_{vinylic}$), 35.58 (s_{br}, 2H, $H_{vinylic}$), -42.82 (s_{br}, 18H, H-5).

^{13}C NMR: (101 MHz, THF-d_8, 298 K): δ (ppm) = not observable due to broad signals.

^{19}F NMR: (378 MHz, THF-d_8, 298 K): δ (ppm) = -33.99 (s_{br}, 6F, F-1).

APCI-MS positive mode: m/z (200 °C) = 857 (2%), 697 (2%, $[UCl_2$(TFB-*t*BuA)$_2]$-H^+), 661 (10%, [UCl(TFB-*t*BuA)$_2]^+$), 264 (10%), 208 (100%), 190 (100%, $[C_8H_7ONF_3]^+$), 140 (90%).

APCI-MS negative mode: m/z (200 °C) = 260 (100%), 191 (100%, $[C_8H_8ONF_3]^-$).

Synthesis of [$NpCl_2$(TFB-*t*BuA)$_2$(THF)] (Np-1)
To a solution of 10.0 mg [$NpCl_4$($DME)_2$] (17.8 µmol, 1.0 equiv.) in 1 mL THF was slowly added a solution of 8.3 mg K-TFB-*t*BuA (**K-1**) (35.6 µmol, 2.0 equiv.) in 1 mL THF. The orange solution was stirred for 18 h at room temperature to give a colorless precipitate that was filtered with a 0.2 µm PTFE syringe filter. All volatiles were removed under reduced pressure to give a brown/orange solid (m = 8.7 mg).

^{1}H NMR: (401.7 MHz, THF-d_8, 298 K): δ (ppm) = 44.98 (s_{br}, 2H, $H_{vinylic}$), 37.11 (s_{br}, 2H, $H_{vinylic}$), -39.29 (s_{br}, 18H, H-5).

^{13}C NMR: (101 MHz, THF-d_8, 298 K): δ (ppm) = not observable due to broad signals.

^{19}F NMR: (378 MHz, THF-d_8, 298 K): δ (ppm) = -14.95 (s_{br}, 6F, F-1).

APCI-MS positive mode: m/z (200 °C) = 441 (40%), 385 (40%), 329 (90%), 208 (100%), 190 (95%, [$C_8H_7ONF_3$]$^+$), 140 (90%).

APCI-MS negative mode: m/z (200 °C) = 680 (20%, [$NpCl_2$(TFB-*t*BuA)($C_7H_8ONF_3$)]$^-$), 383 (20%), 194 (100%, [TFB-*t*BuA]$^-$).

Synthesis of [Np(PyTFP)$_4$] (Np-2)
To a solution of 10.0 mg [$NpCl_4$($DME)_2$] (17.8 µmol, 1.0 equiv.) in 1 mL THF was added dropwise a THF (1 mL) solution of 16.2 mg K-PyTFP (**K-2**) (71.5 µmol, 4.0 equiv.). The resulting orange solution was stirred for 18 h at room temperature to give a colorless precipitate that was filtered with a 0.2 µm PTFE syringe filter. All volatiles were removed under reduced pressure to give a bright yellow solid (m = 16.2 mg, 92% yield). Yellow single crystals suitable for SC-XRD analysis were grown by slow evaporation of THF solutions at room temperature.

^{1}H NMR: (401.7 MHz, THF-d_8, 233 K): δ (ppm) = 12.86 (s_{br}, 4H, H-8), 7.95 – 7.92 (dd, $^{3}J_{H,H}$ = 7.2 Hz, 4H, H-6), 6.53 (s, 4H, H-7), 5.80 – 5.78 (d, $^{3}J_{H,H}$ = 7.2 Hz, 4H, H-5), 0.96 (s_{br}, 4H, H-3).

^{13}C NMR: (101 MHz, THF-d_8, 233 K): δ (ppm) = 177.8 – 169.6 (q, $^{1}J_{F,C}$ = 280 Hz, C-1), 154.5 (s, C-4), 151.3 (s, C-5), 131.2 (s, C-7), 122.6 (s, C-6), 85.4 (s, C-8), 75.2 (s, C-3), -9.42 (s, C-2).

^{19}F NMR: (378 MHz, THF-d_8, 233 K): δ (ppm) = -68.47 (s, $^{1}J_{F,C}$ = 280 Hz, 12F, F-1).

APCI-MS positive mode: m/z (200 °C) = 801.3 (100%, [Np(PyTFP)$_3$]$^+$), 781.1 (13%, [Np(PyTFP)$_2$($C_8H_4F_2NO$)]$^+$), 632.1 (3%, [Np(PyTFP)$_2$F]$^+$), 319 (25%), 299 (10%), 291 (5%), 189.9 (38%, [H-PyTFP]$^+$), 169.9 (5%, [$C_8H_6F_2NO$]$^+$), 149.9 (5%, [C_8H_5FNO]$^+$), 142.0 (10%), 120.0 (22%, [C_7H_6NO]$^+$), 93.1 (10%, [C_6H_6N]$^+$).

APCI-MS negative mode: m/z (200 °C) = 989.2 (100%, [Np(PyTFP)$_4$-H]$^-$), 817.1 (2%, [Np(PyTFP)$_3$O]$^-$), 187.8 (90%, [PyTFP]$^-$).

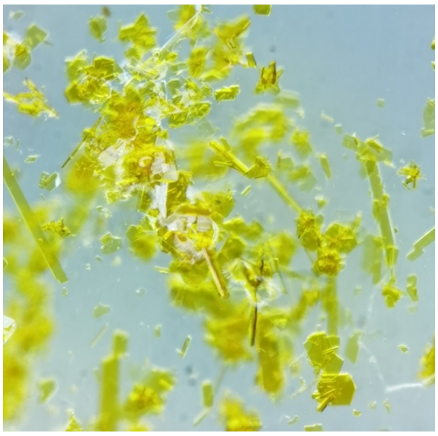

Figure S1. Photograph of crystalline **Np-2** under mineral oil.

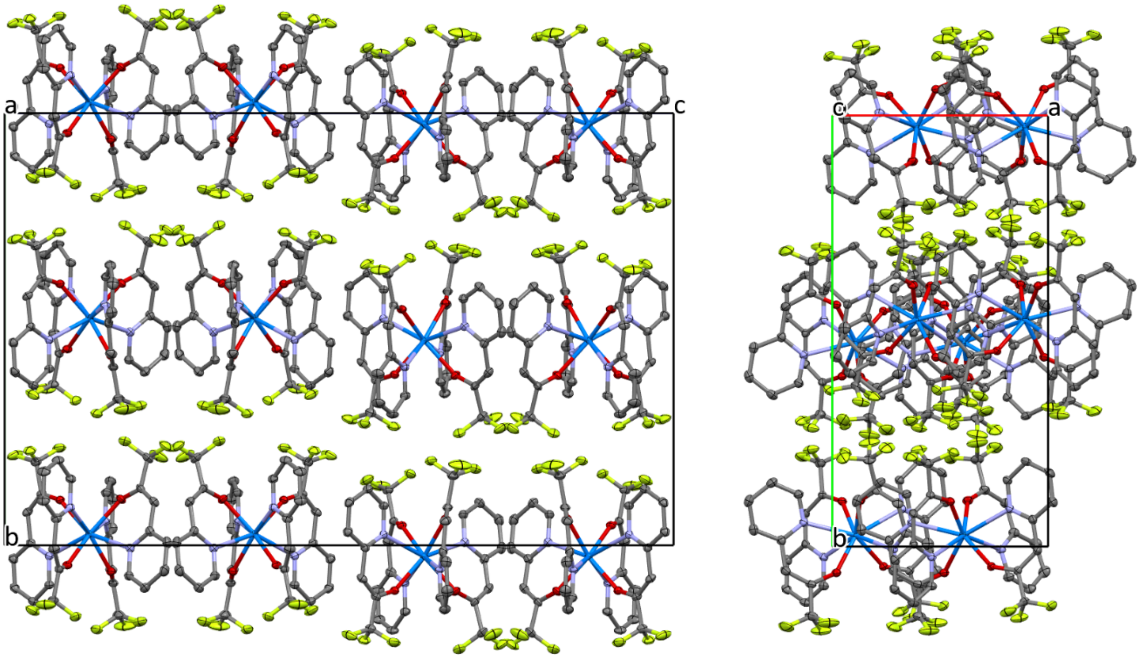

Figure S2. Crystal packing of isolated **Np-2** molecules in the direction of the crystallographic a-axis (left) and c-axis (right).

Synthesis of [Np(DMOTFP)$_4$] (Np-3)

To a solution of 10.0 mg [NpCl$_4$(DME)$_2$] (17.8 µmol, 1.0 equiv.) in 1 mL THF was added dropwise a THF (1 mL) solution of 17.5 mg K-DMOTFP (**K-3**) (71.5 µmol, 4.0 equiv.). The resulting orange-brown solution was stirred for 18 h at room temperature to give a colorless precipitate that was filtered with a 0.2 µm PTFE syringe filter. All volatiles were removed under reduced pressure to give a dark orange solid (m = 16.8 mg, 89% yield). Orange single crystals suitable for SC-XRD analysis were grown by slow evaporation of THF solutions at room temperature.

^{1}H NMR: (401.7 MHz, THF-d$_8$, 223 K): δ (ppm) = 7.44 (s$_{br}$, 4H, H-3), 5.85 (s$_{br}$, 4H, H-3'), 2.52 (s$_{br}$, 12H, H-6'), -2.20 (s$_{br}$, 12H, H-6), -3.09 (s$_{br}$, 12H, H-8'), -10.07 (s$_{br}$, 12H, H-8).

^{13}C NMR: (101 MHz, THF-d_8, 223 K): δ (ppm) = 181 (q, C-1), 178 (q, C-1'), 176.05 (s, C-7'), 169.79 (s, C-4'), 155.10 (s, C-7), 115.26 (s, C-5'), 98.26 (s, C-2), 93.56 (s, C-2'), 67.9 (s, C-5) 47.04 (s, C-3), 21.37 (s, C-3'), 8.74 (s, C-8') 8.61 (s, C-6'), 1.52 (s, C-6), -10.50 (s, C-8).

^{19}F NMR: (378 MHz, THF-d_8, 223 K): δ (ppm) = -57.19 (s, $^{1}J_{F,C}$ = 280 Hz, 12F, F-1), -66.97 (s, $^{1}J_{F,C}$ = 280 Hz, 12F, F-1').

APCI-MS positive mode: m/z (200 °C) = 1042.2 (5%, $[Np(DMOTFP)_3(C_8H_7F_2NO_2)]^+$), 871.2 (15%, $[Np(DMOTFP)_3NH]^+$), 855.4 (100%, $[Np(DMOTFP)_3]^+$), 835.2 (3%, $[Np(DMOTFP)_2(C_8H_5F_2NO_2)]^+$), 734.2 (4%, $[Np(C_8H_4FNO_2)_3]^+$), 355.2 (6%), 207.9 (100%, $[H\text{-}DMOTFP]^+$), 187.9 (5%, $[C_8H_6F_2NO_2]^+$), 159.9 (25%, $[C_6HF_2NO_2]^+$), 138.0 (70%, $[C_7H_8NO_2]^+$).

APCI-MS negative mode: m/z (200 °C) = 1061.1 (100%, $[Np(PyTFP)_4\text{-}H]^-$), 205.9 (8%, $[DMOTFP]^-$).

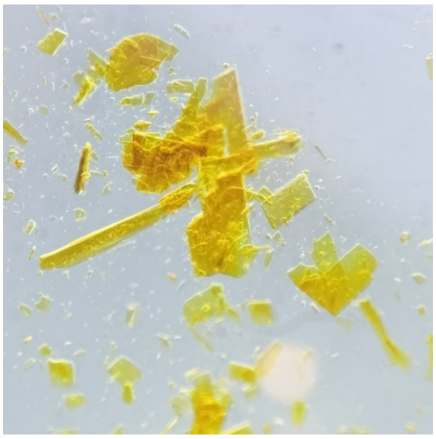

Figure S3. Photograph of crystalline **Np-3** under mineral oil.

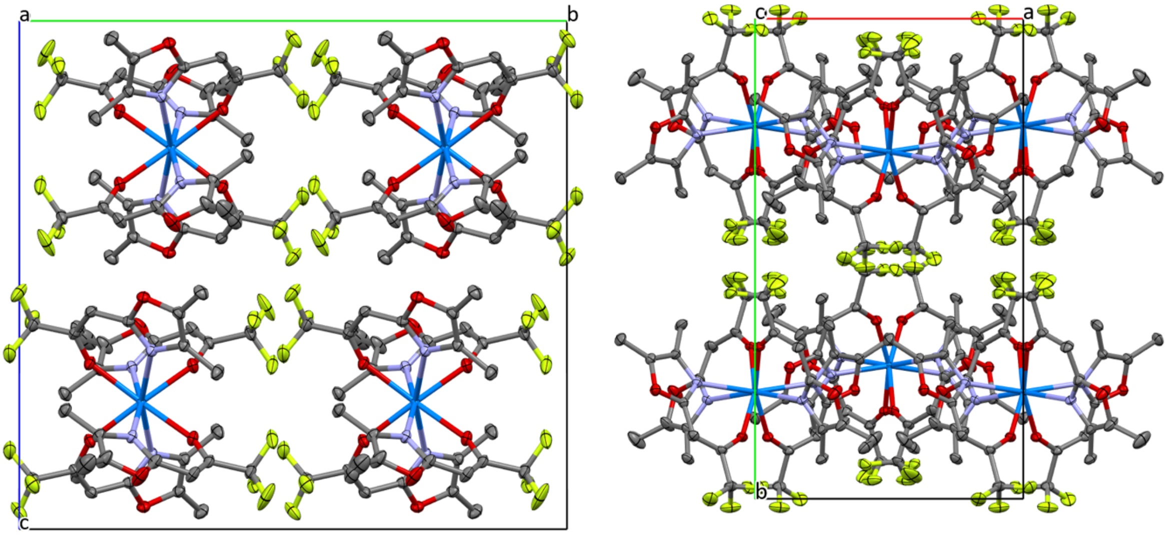

Figure S4. Crystal packing of isolated **Np-3** molecules in the direction of the crystallographic a-axis (left) and c-axis (right).

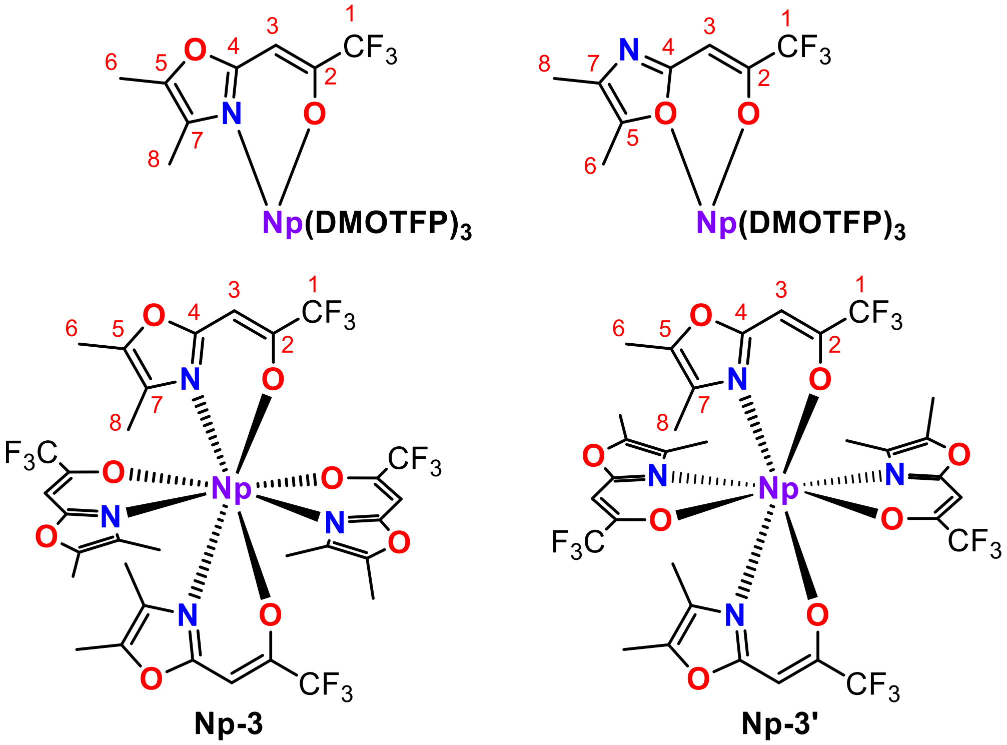

Scheme S1. Possible binding motifs for DMOTFP (**3**).

NMR Spectra

[UCl2(TFB-*t*BuA)2(THF)] (U-1)

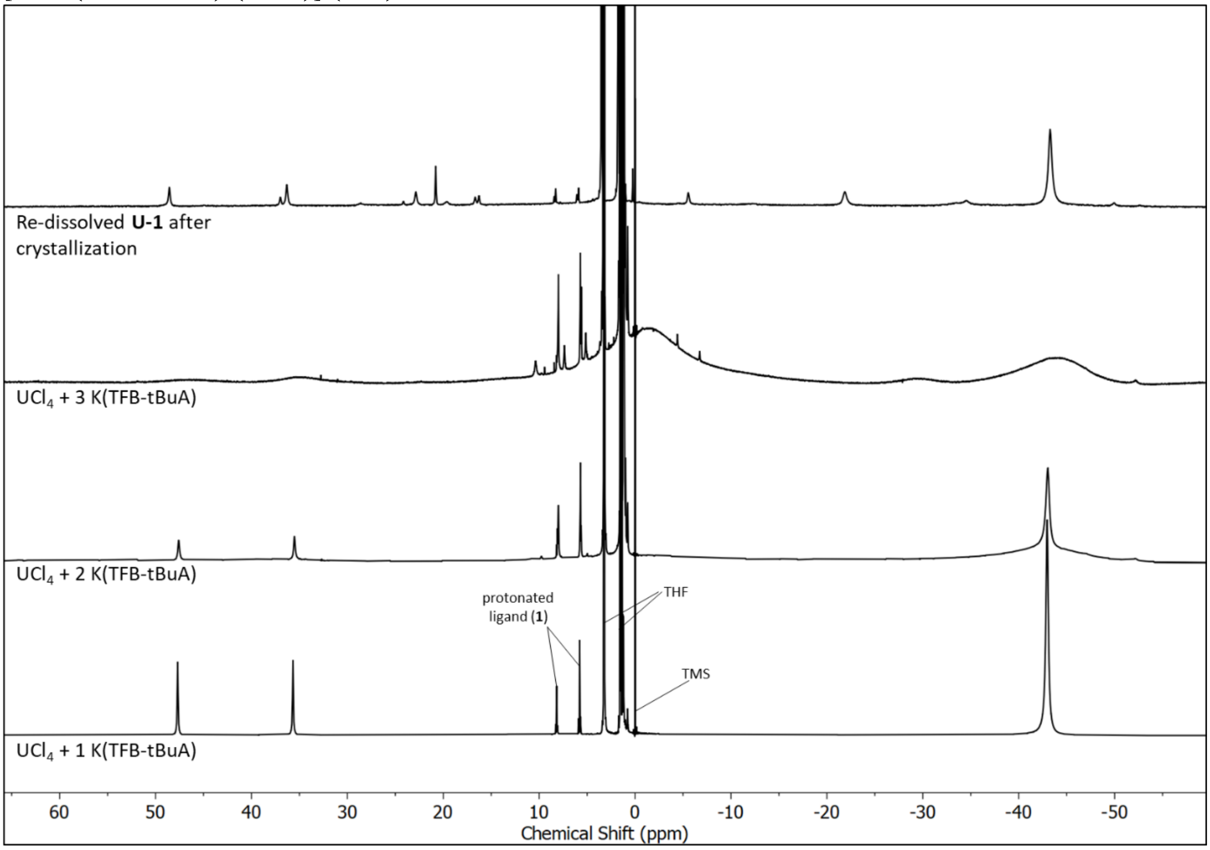

Figure S5. ¹H-NMR spectra of reactions between UCl_4 and different equivalents of K(TFB-*t*BuA) (**K-1**) in THF-d8 at 298K.

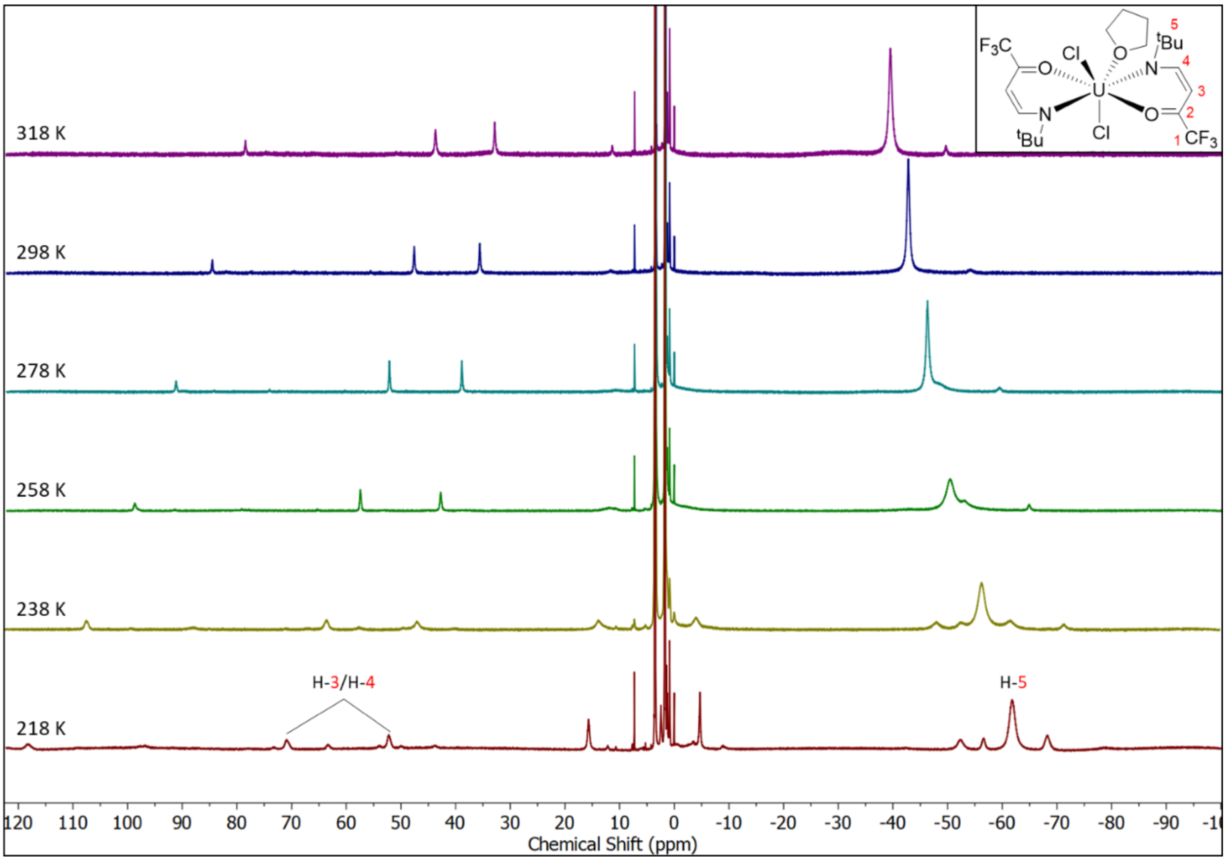

Figure S6. ^{1}H VT-NMR spectra of **U-1** between 218 and 318K in THF-d8.

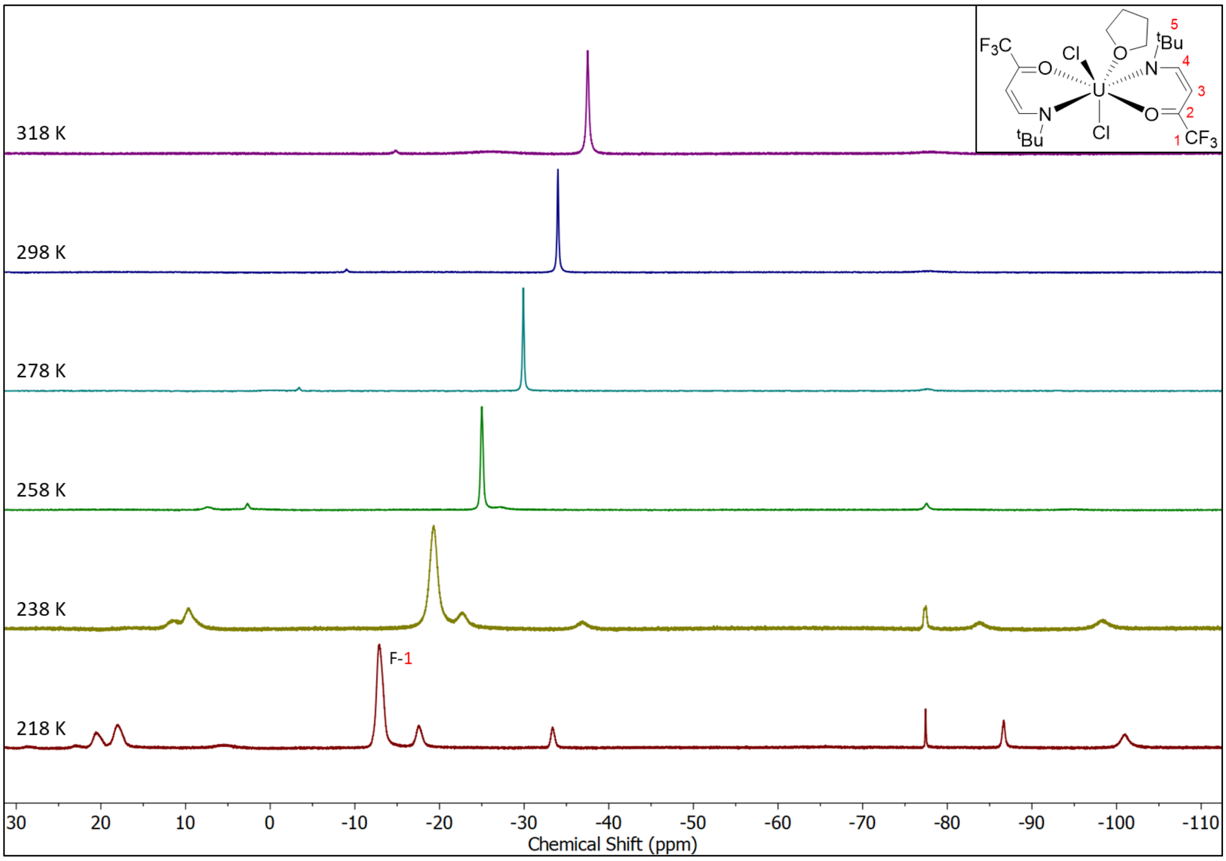

Figure S7. ^{19}F VT-NMR spectra of **U-1** between 218 and 318K in THF-d8.

[$NpCl_2$(TFB-*t*BuA)$_2$(THF)] (Np-1)

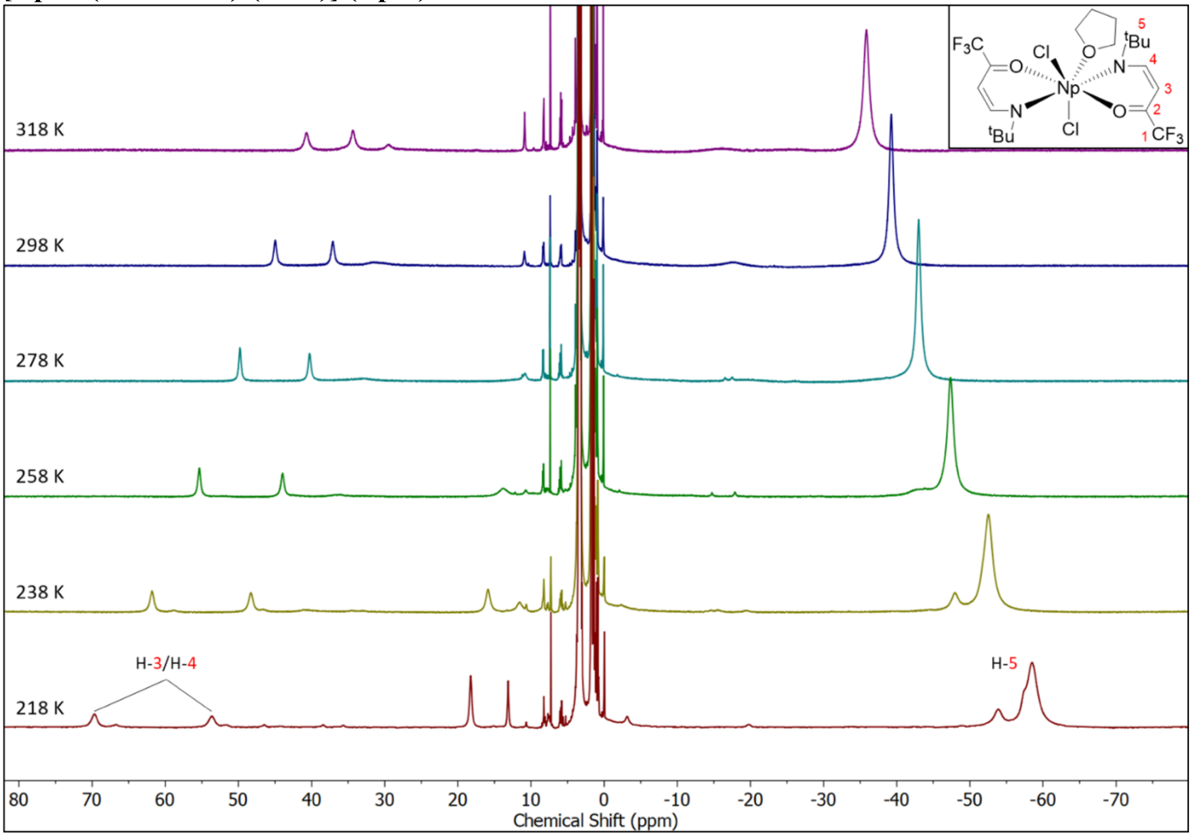

Figure S8. ^{1}H VT-NMR spectra of **Np-1** between 218 and 318K in THF-d8.

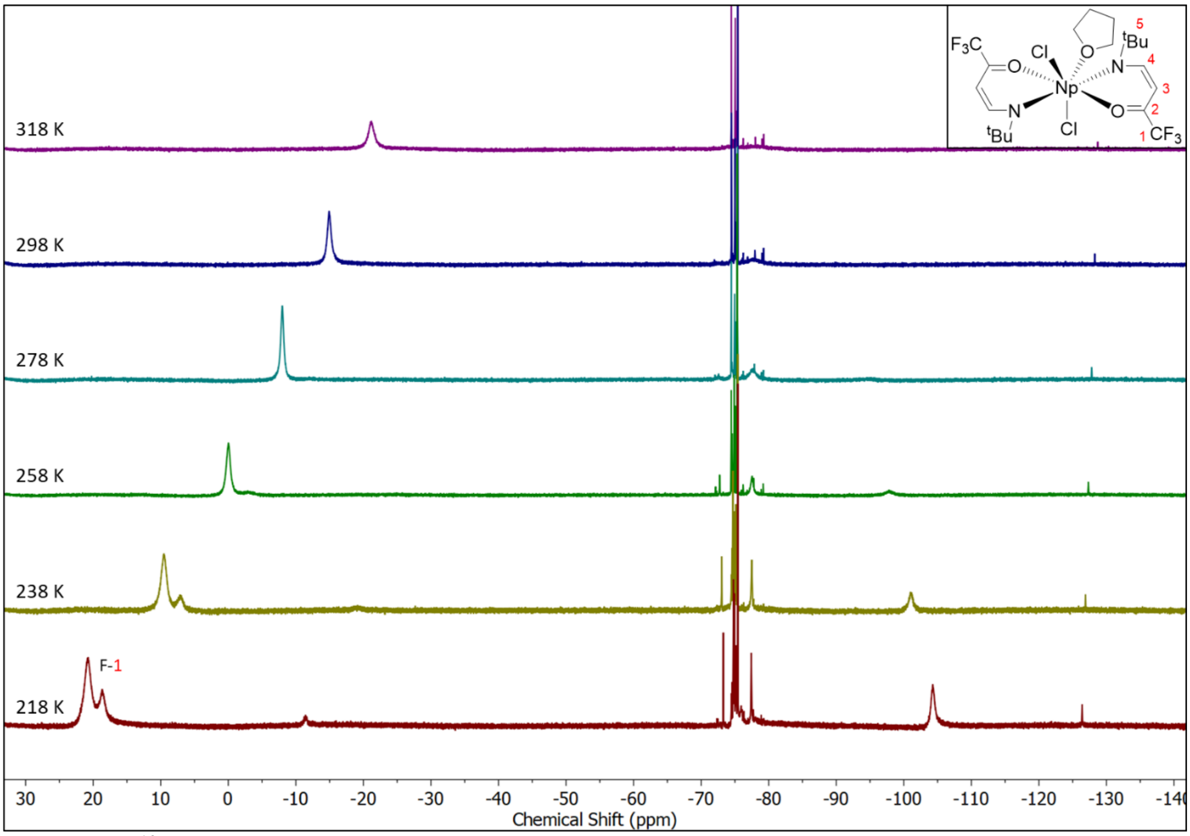

Figure S9. ^{19}F VT-NMR spectra of **Np-1** between 218 and 318K in THF-d8.

[Np(PyTFP)$_4$] (Np-2)

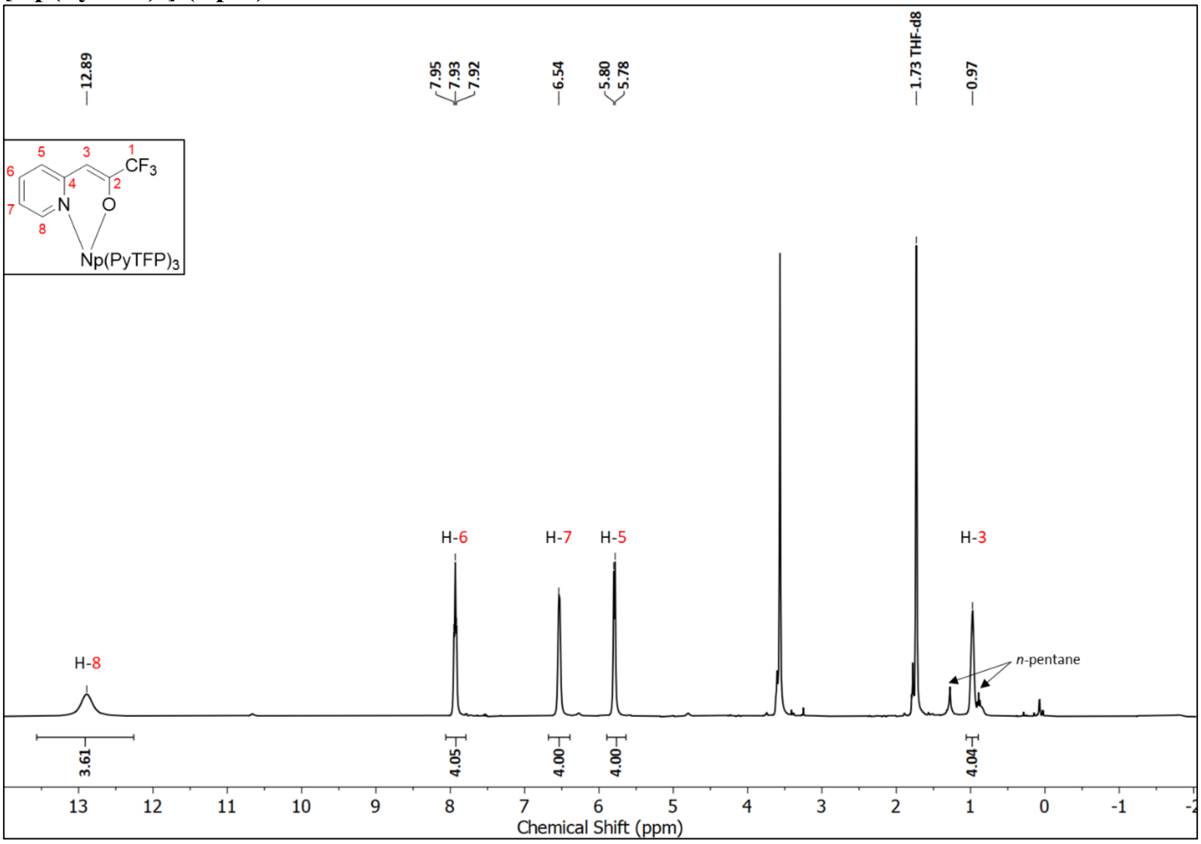

Figure S10. ^{1}H NMR spectrum of **Np-2** in THF-d8 at 233K.

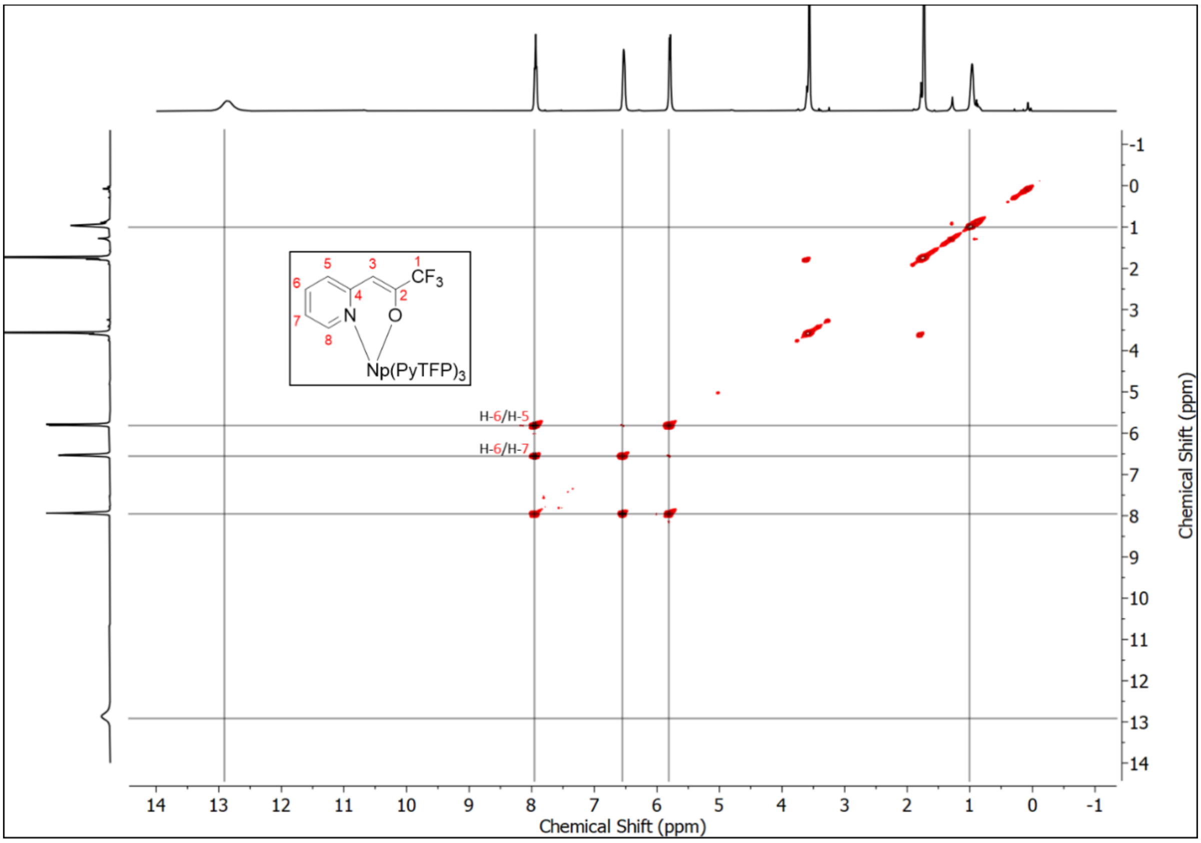

Figure S11. ^{1}H^{1}H COSY experiment of **Np-2** in THF-d8 at 233K.

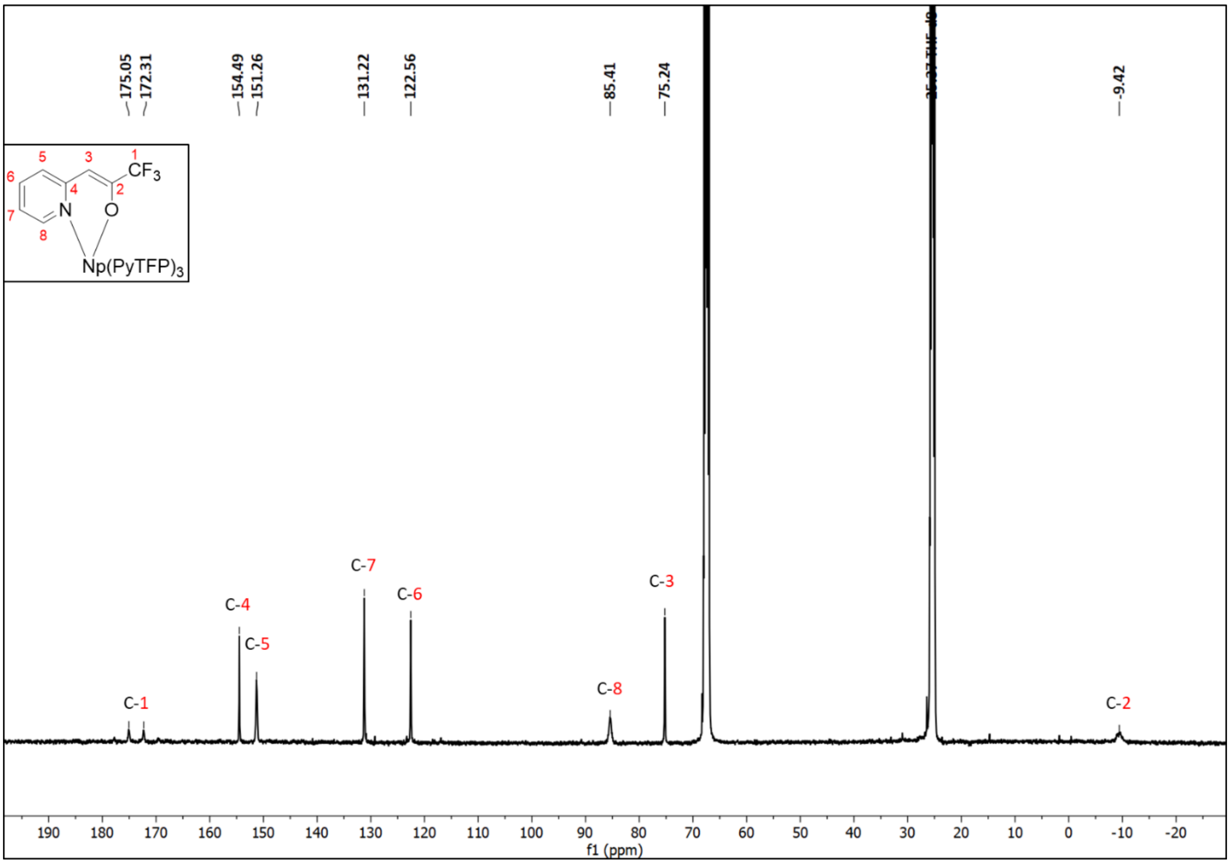

Figure S12. ^{13}C NMR spectrum of **Np-2** in THF-d8 at 233K.

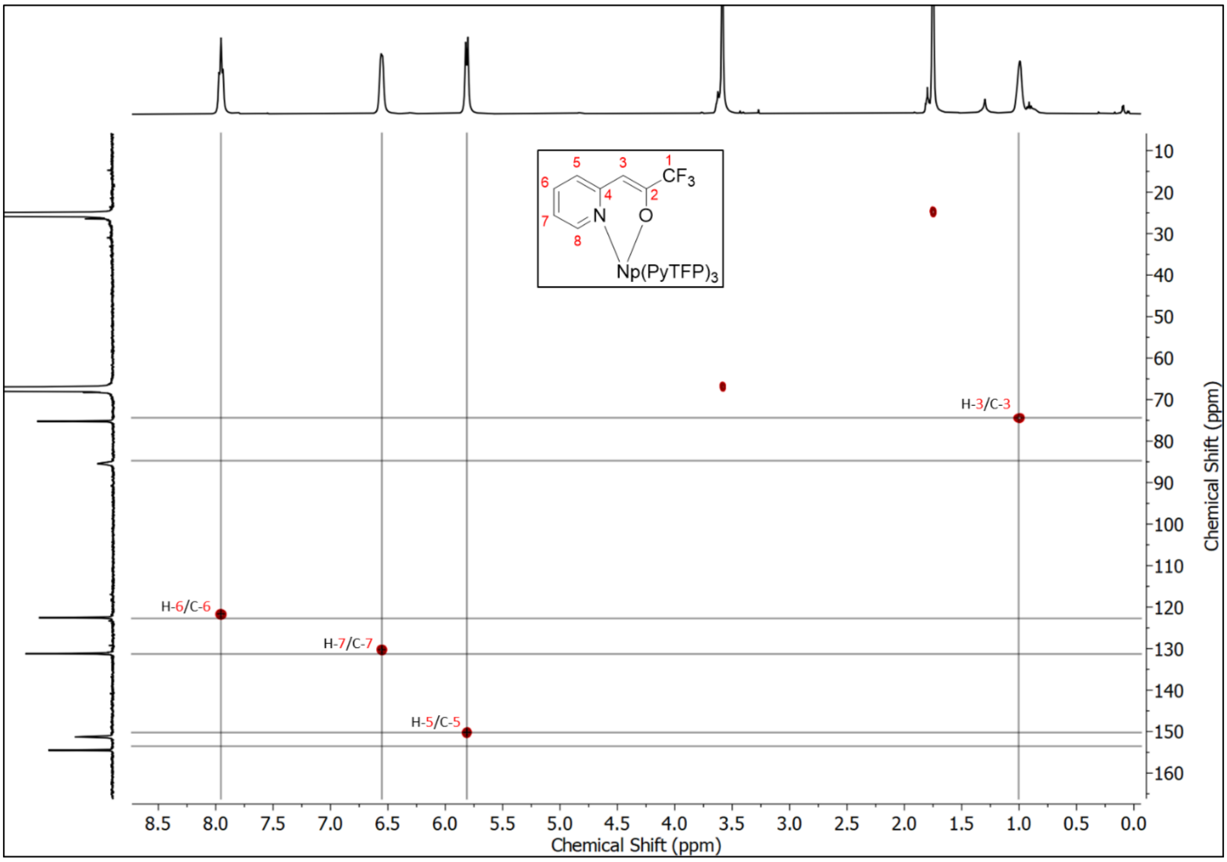

Figure S13. $^{1}H^{13}C$ HSQC experiment of **Np-2** in THF-d8 at 233K.

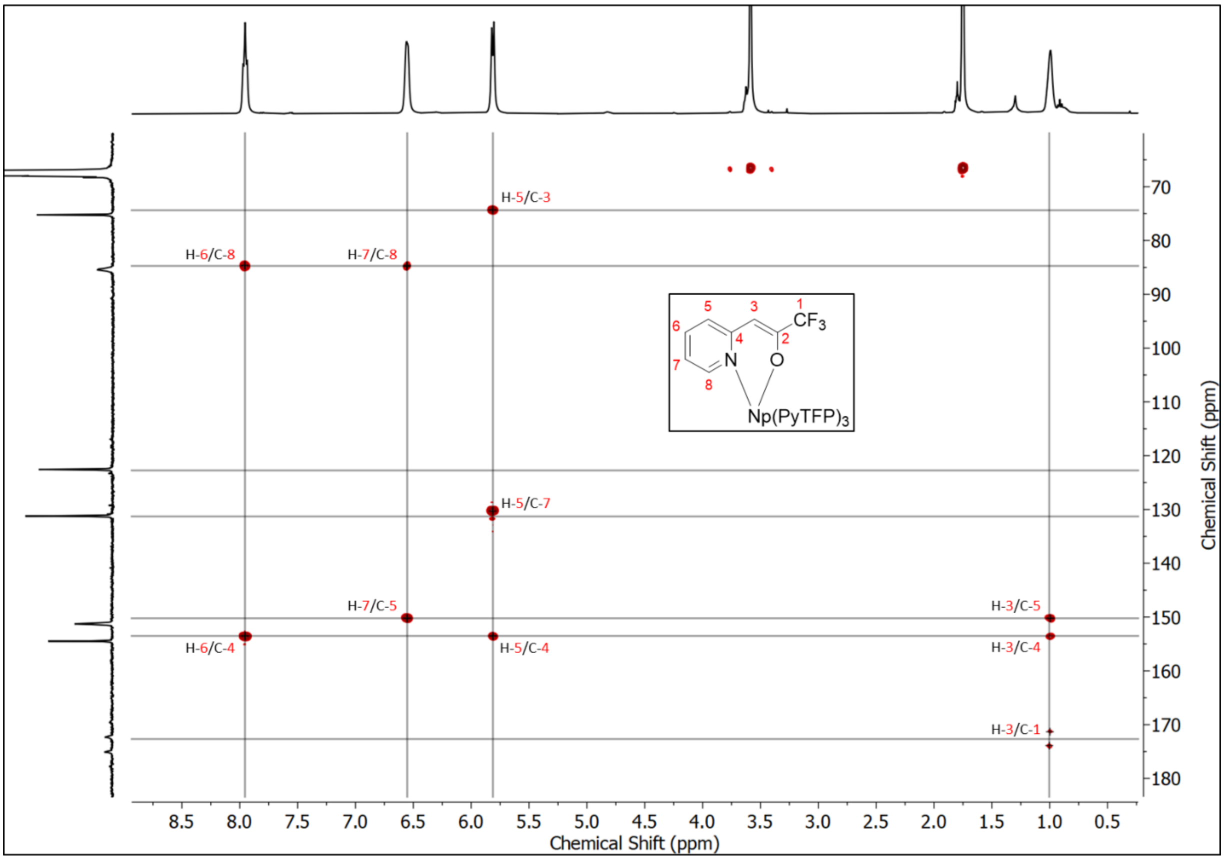

Figure S14. $^{1}H^{13}C$ HMBC experiment of **Np-2** in THF-d8 at 233K.

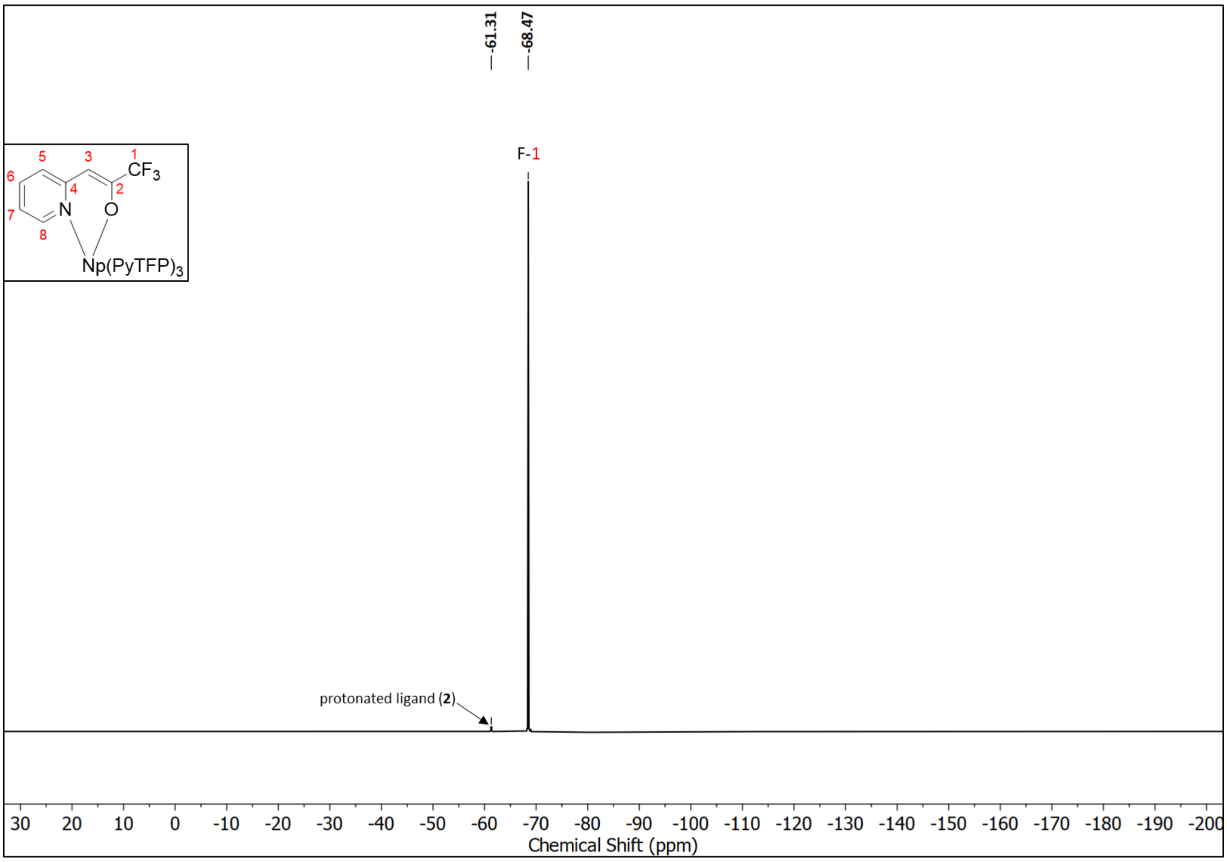

Figure S15. ^{19}F NMR spectrum of **Np-2** in THF-d8 at 233K.

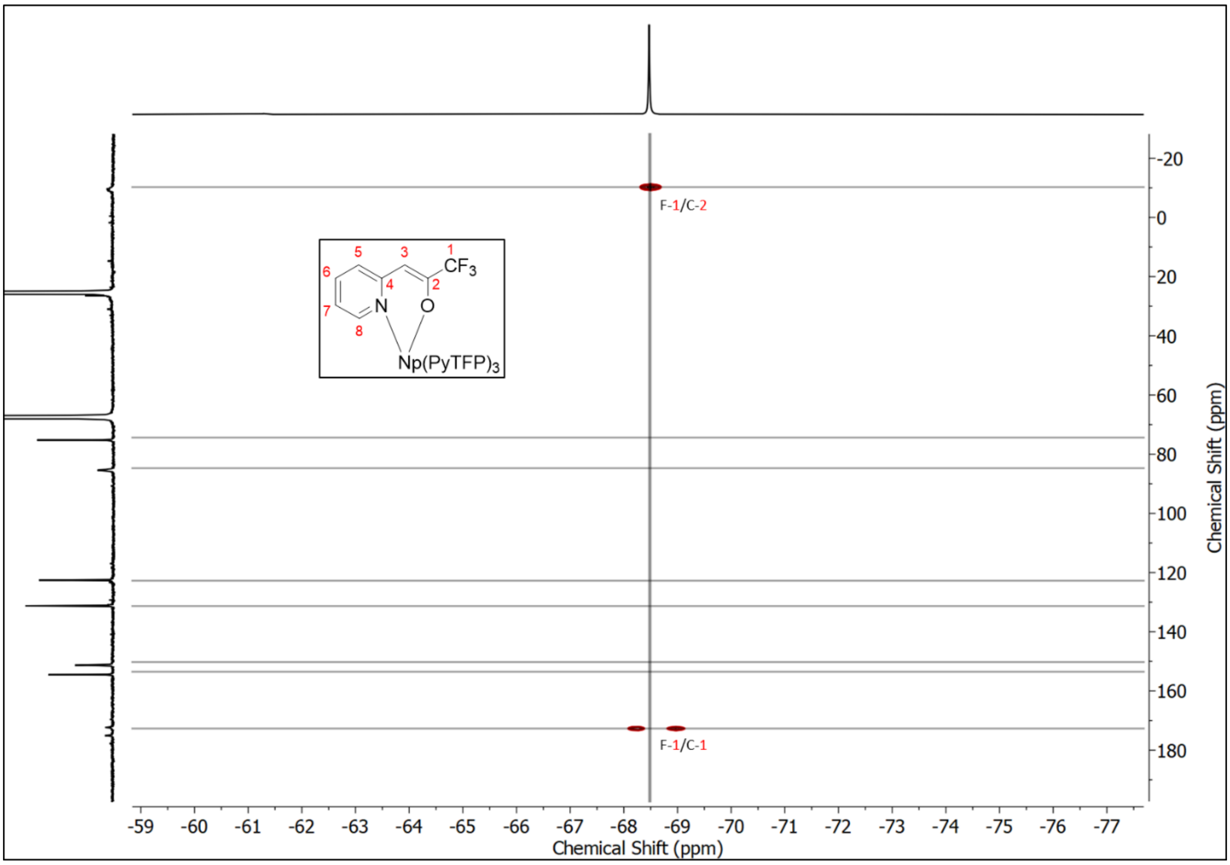

Figure S16. $^{19}F^{13}C$ HMBC experiment of **Np-2** in THF-d8 at 233K.

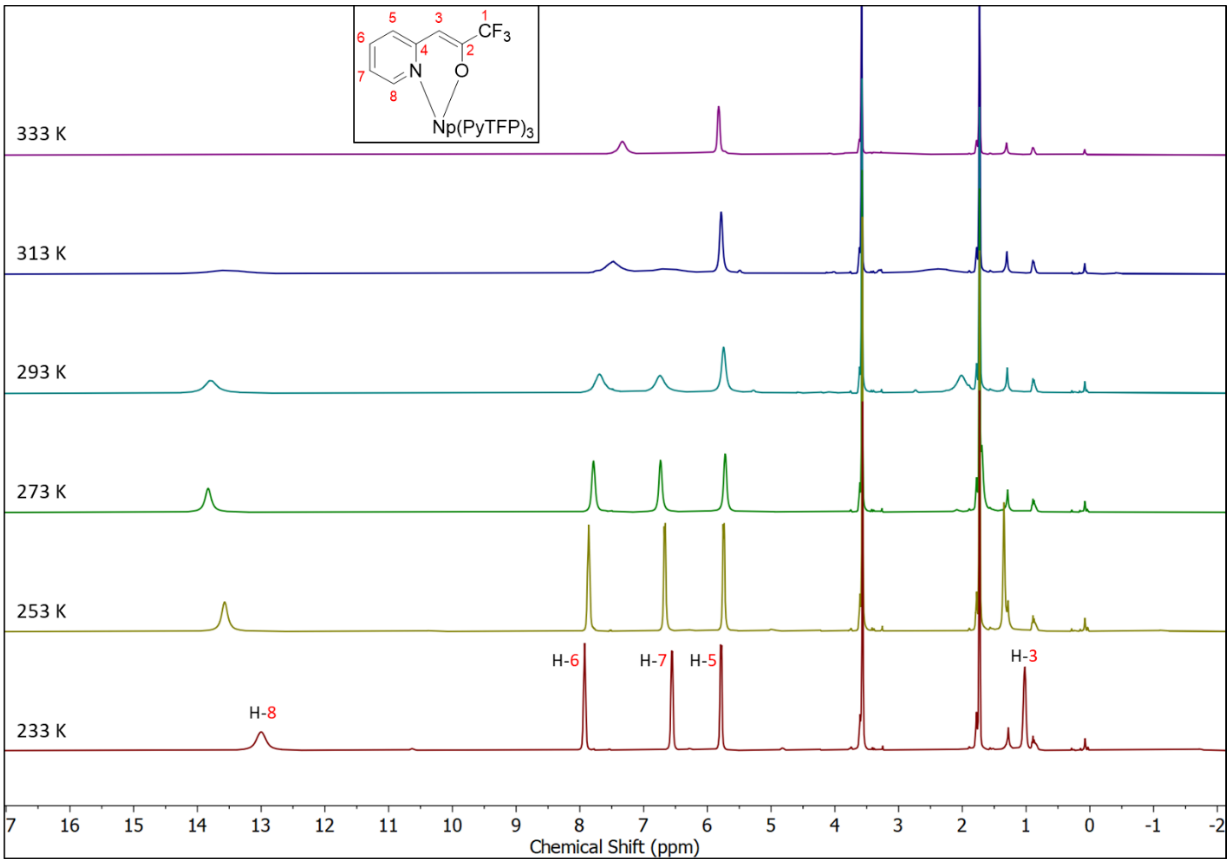

Figure S17. ^{1}H VT-NMR spectra of **Np-2** between 233 and 333K in THF-d8.

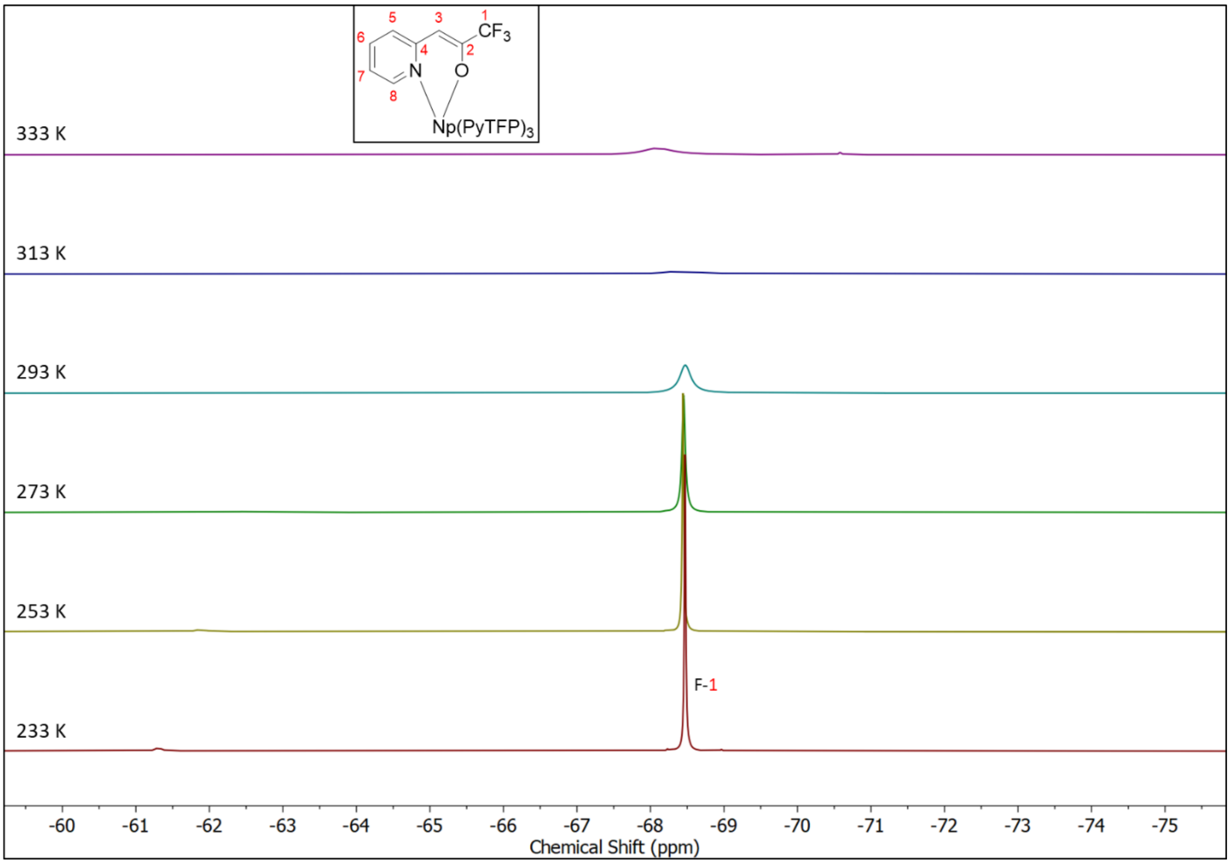

Figure S18. ^{19}F VT-NMR spectra of **Np-2** between 233 and 333K in THF-d8.

[Np(DMOTFP)$_4$] (Np-3)

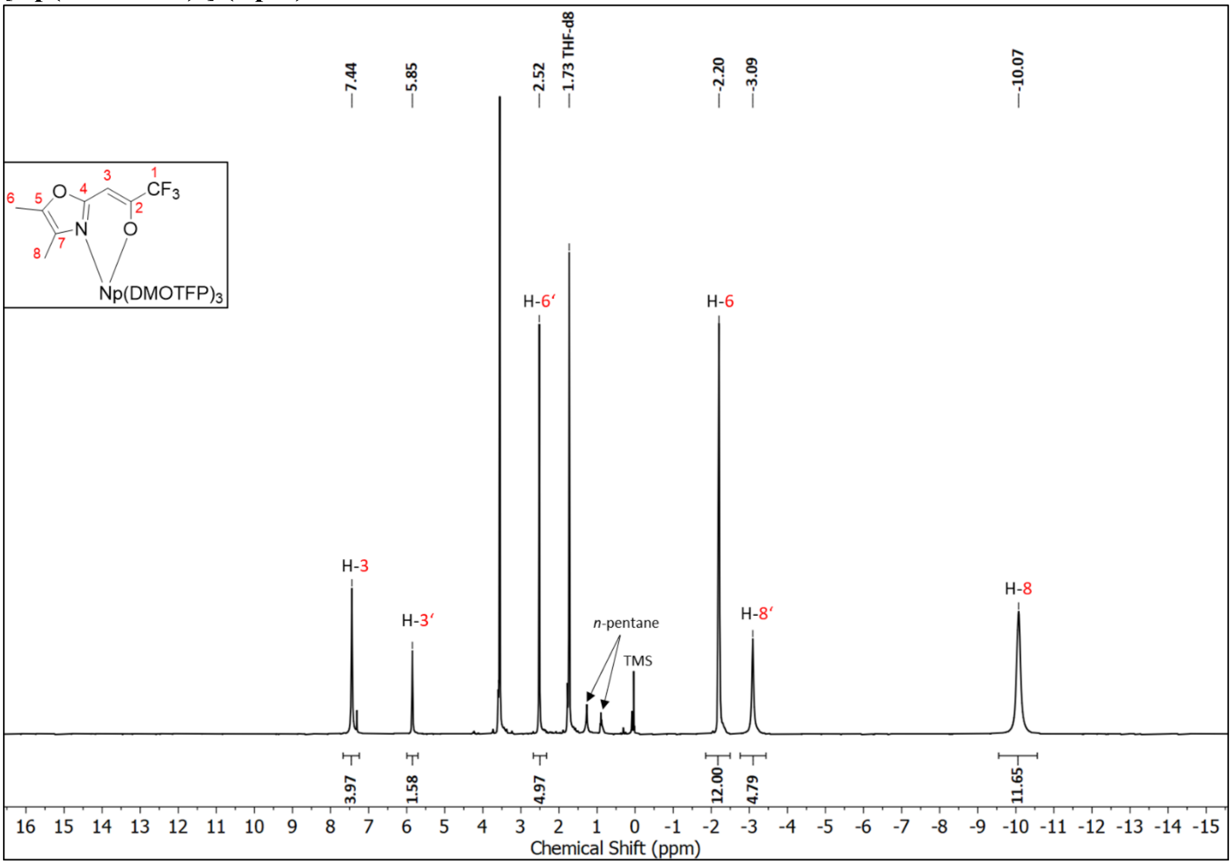

Figure S19. ^{1}H NMR spectrum of **Np-3** and **Np-3’** in THF-d8 at 223K.

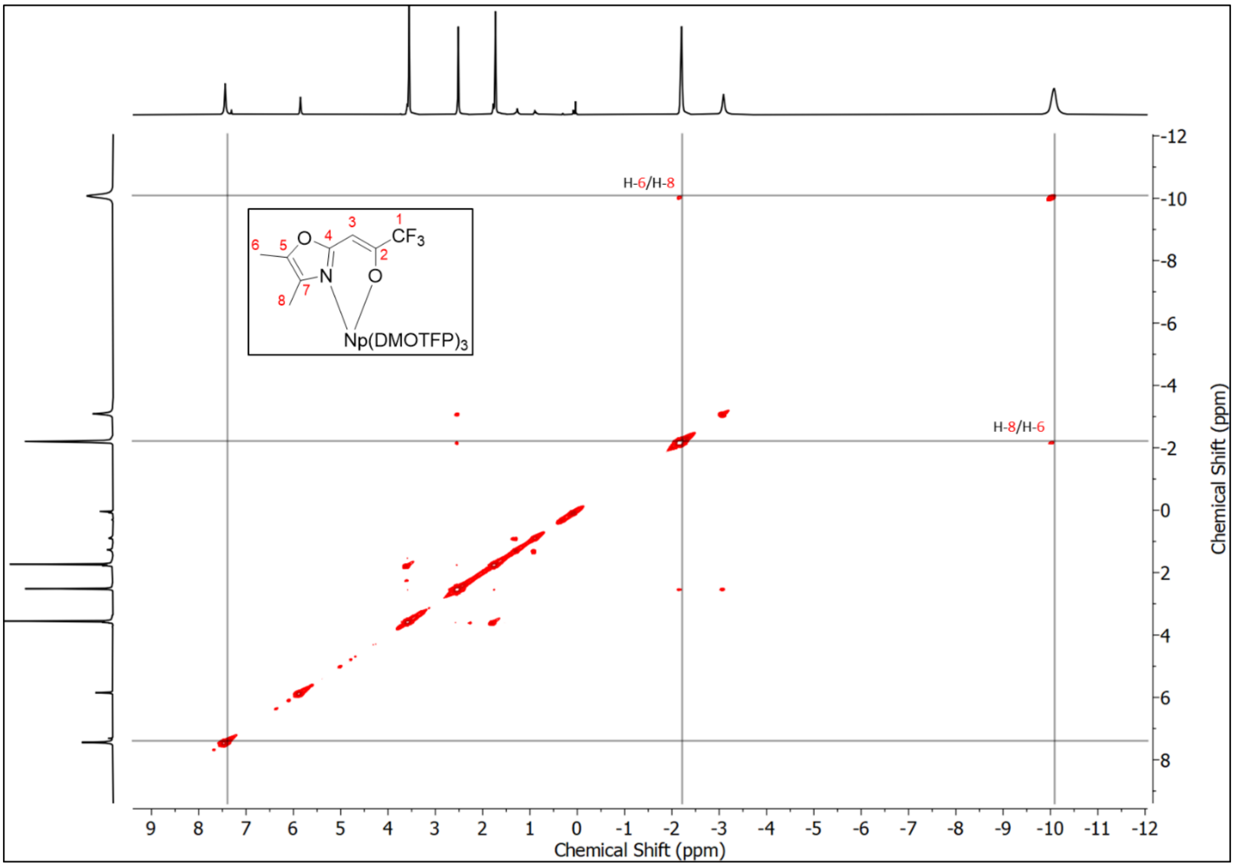

Figure S20. ¹H¹H COSY experiment of **Np-3** in THF-d8 at 223K.

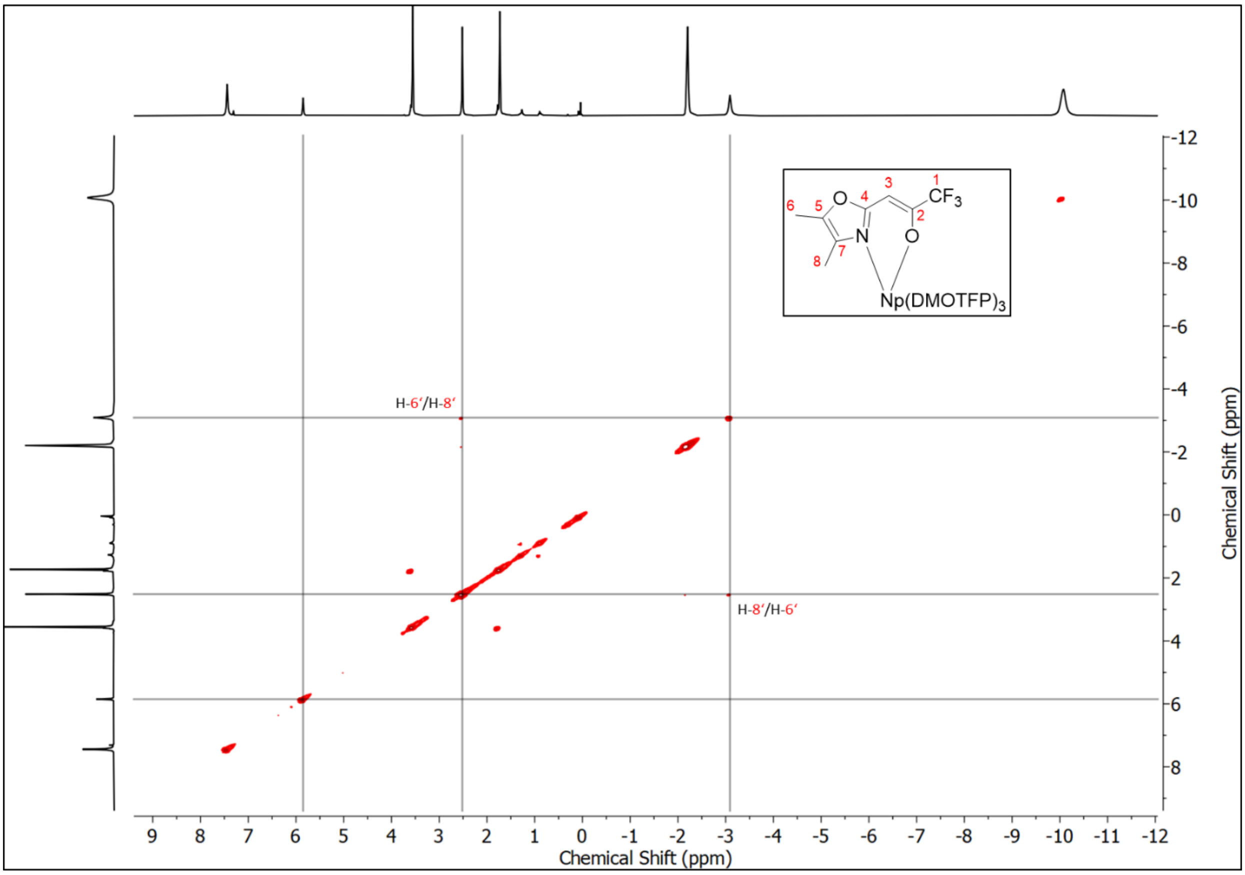

Figure S21. ¹H¹H COSY experiment of **Np-3’** in THF-d8 at 223K.

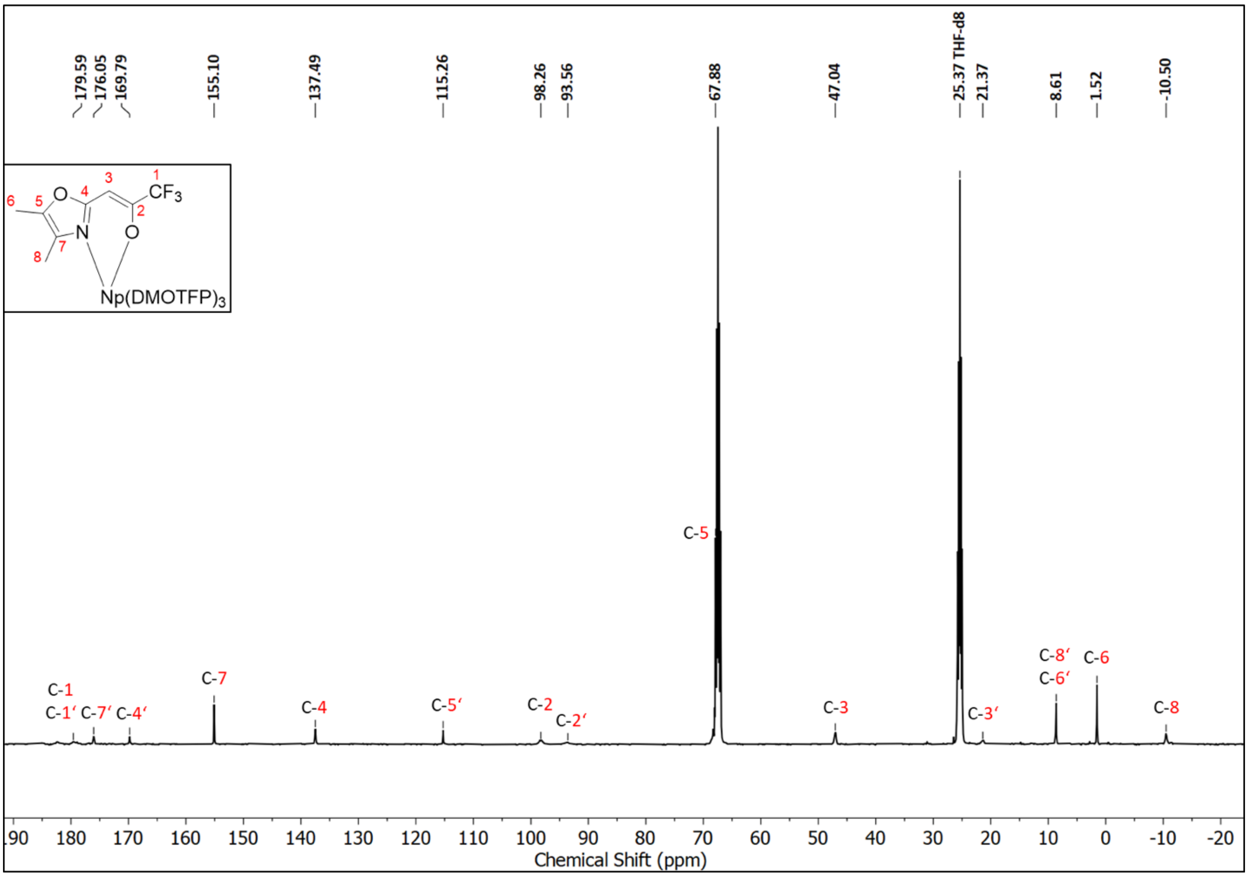

Figure S22. ^{13}C NMR spectrum of **Np-3** and **Np-3’** in THF-d8 at 223K.

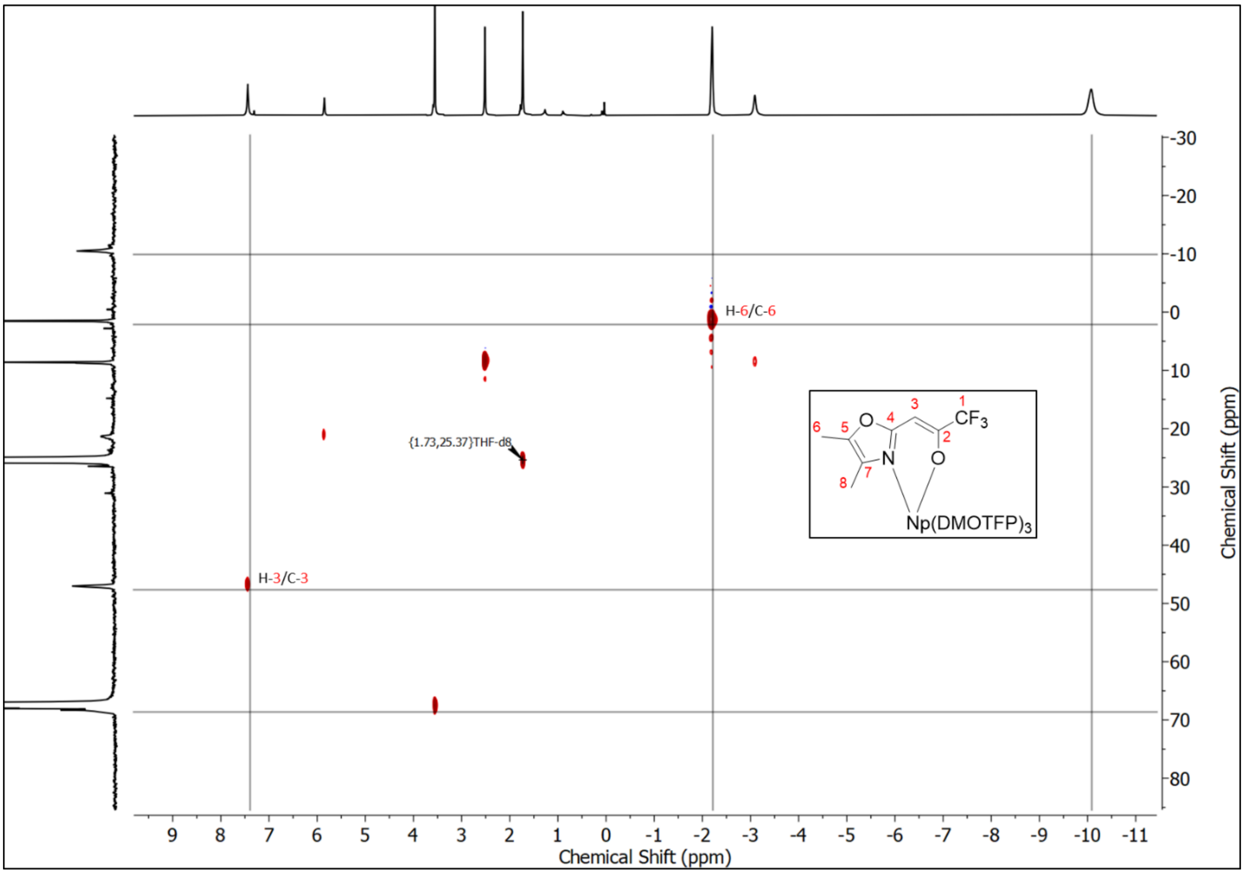

Figure S23. $^{1}H^{13}C$ HSQC experiment of **Np-3** in THF-d8 at 223K.

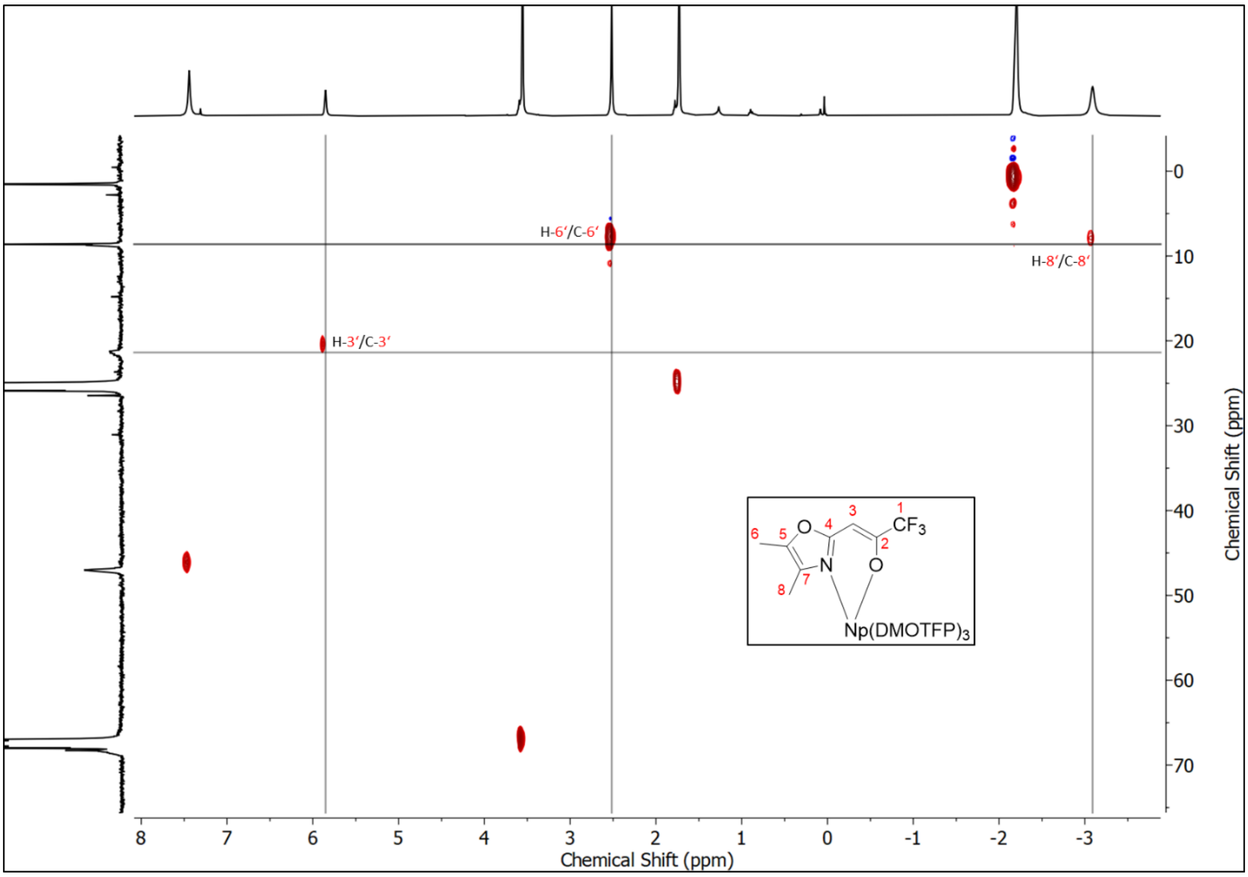

Figure S24. $^{1}H^{13}C$ HSQC experiment of **Np-3’** in THF-d8 at 223K.

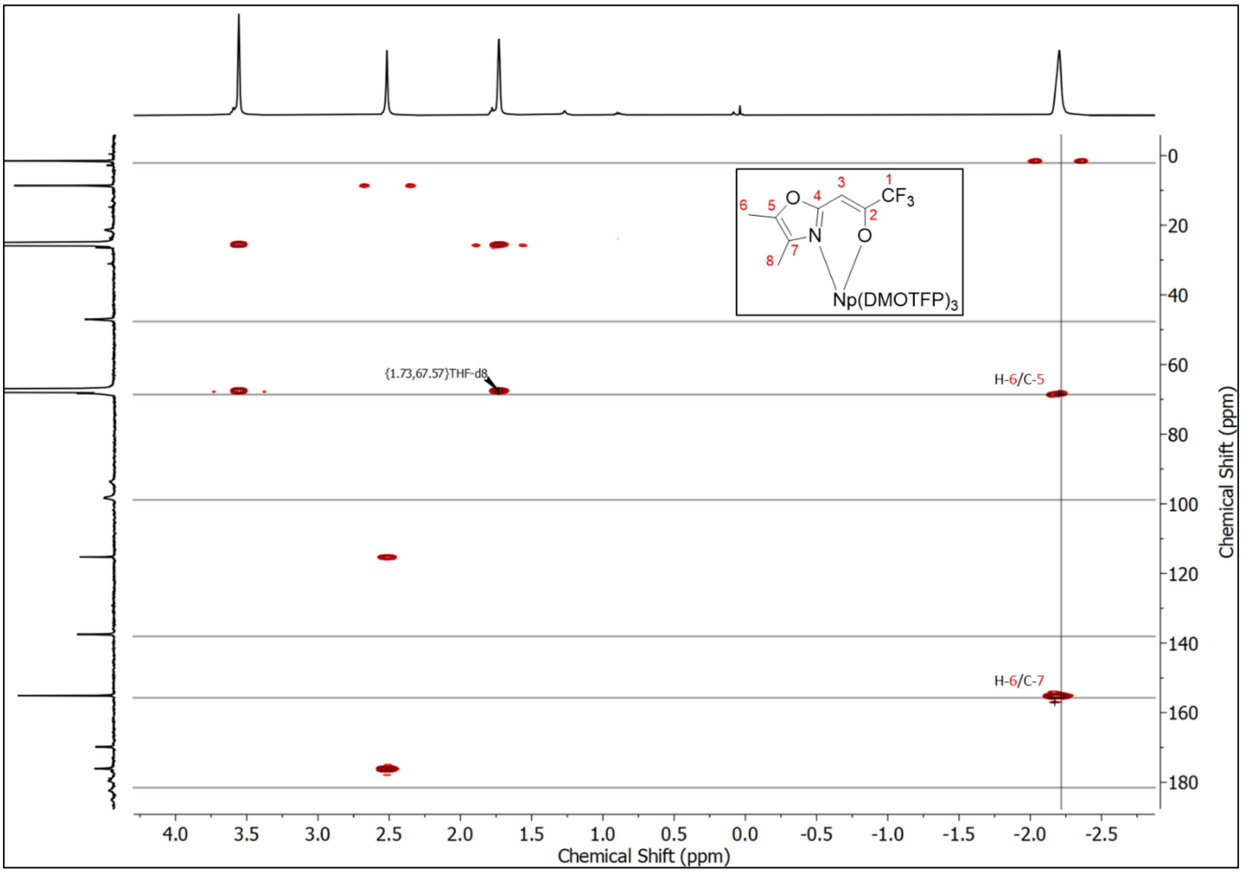

Figure S25. $^{1}H^{13}C$ HMBC experiment of **Np-3** in THF-d8 at 223K.

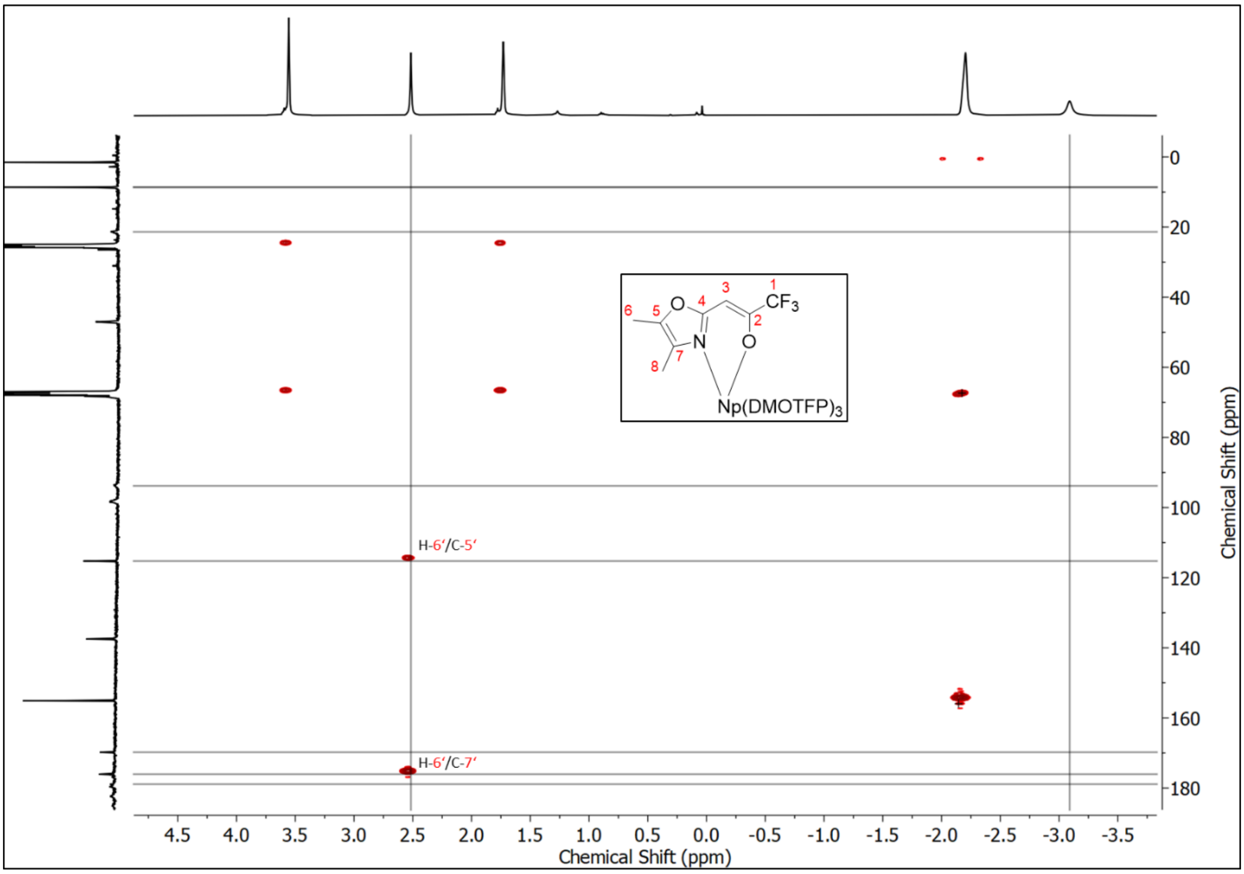

Figure S26. $^{1}H^{13}C$ HMBC experiment of **Np-3'** in THF-d8 at 223K.

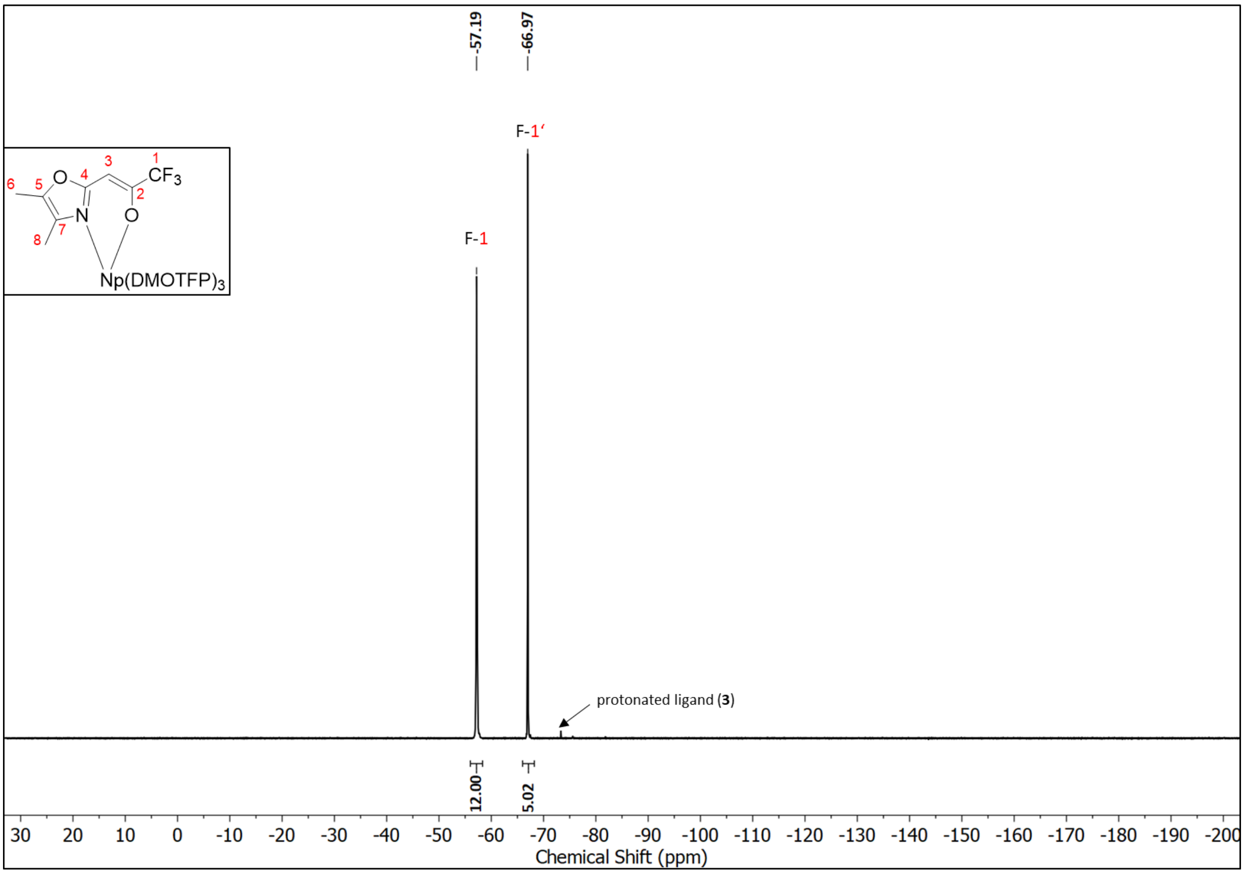

Figure S27. ^{19}F NMR spectrum of **Np-3** and **Np-3'** in THF-d8 at 223K.

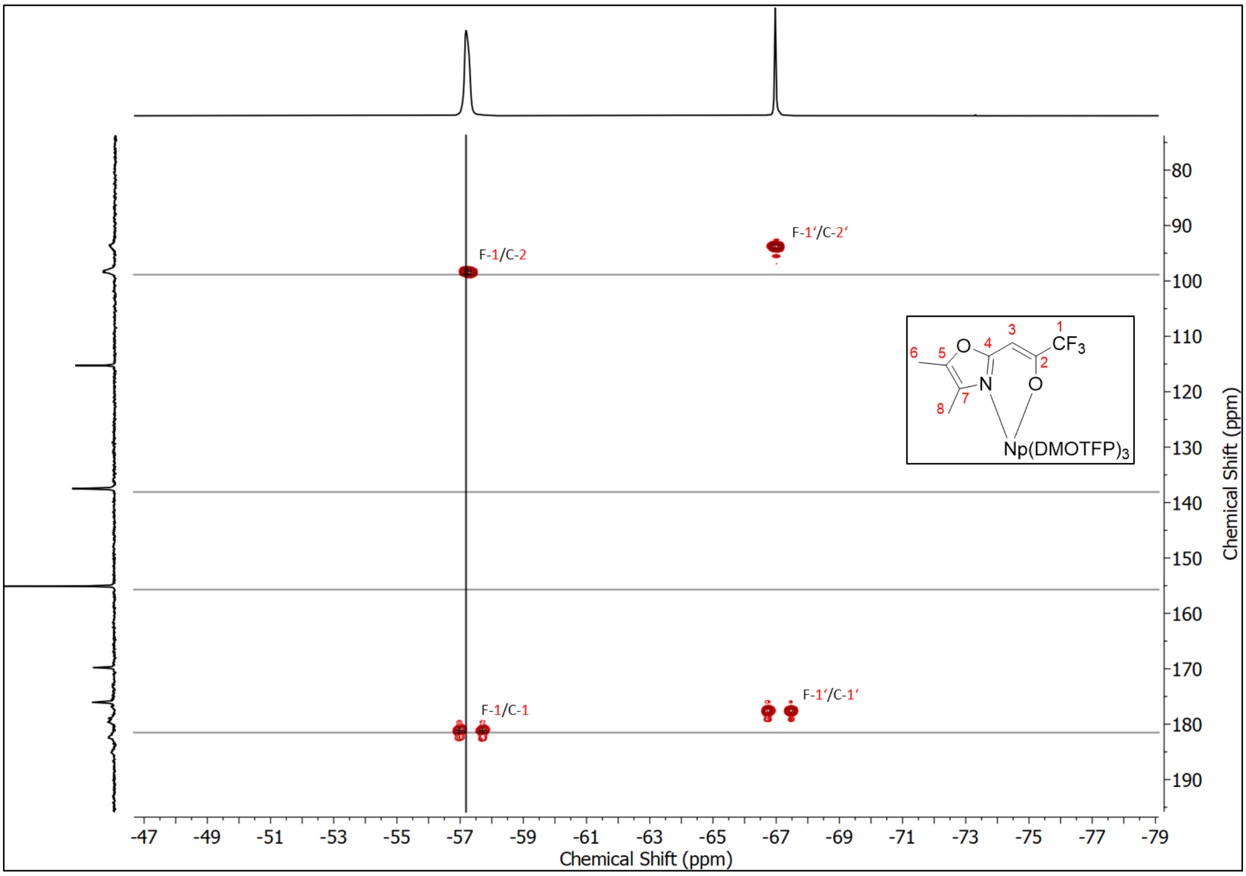

Figure S28. ^{19}F-^{13}C HMBC experiment of **Np-3** and **Np-3'** in THF-d8 at 223K.

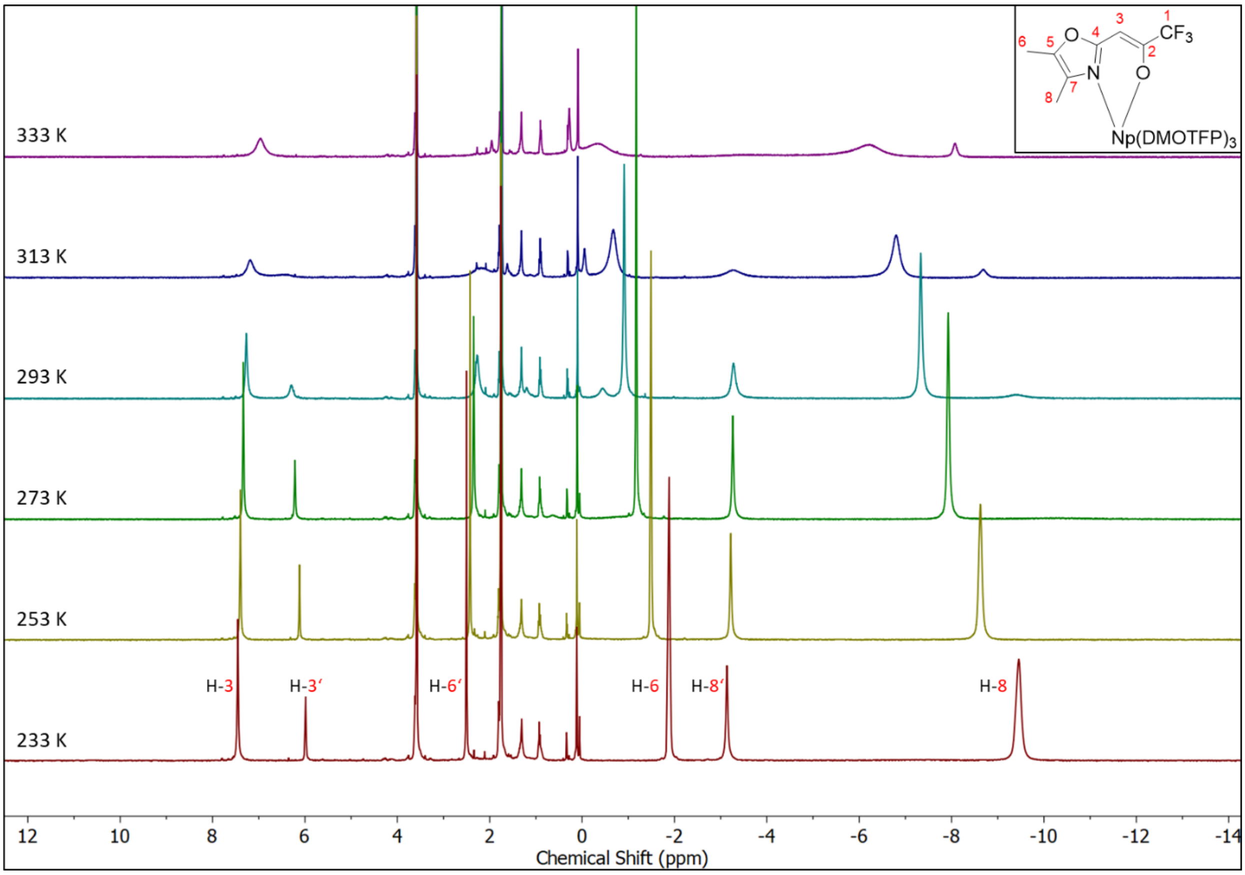

Figure S29. ^{1}H VT-NMR spectra of **Np-3** and **Np-3'** between 233 and 333K in THF-d8.

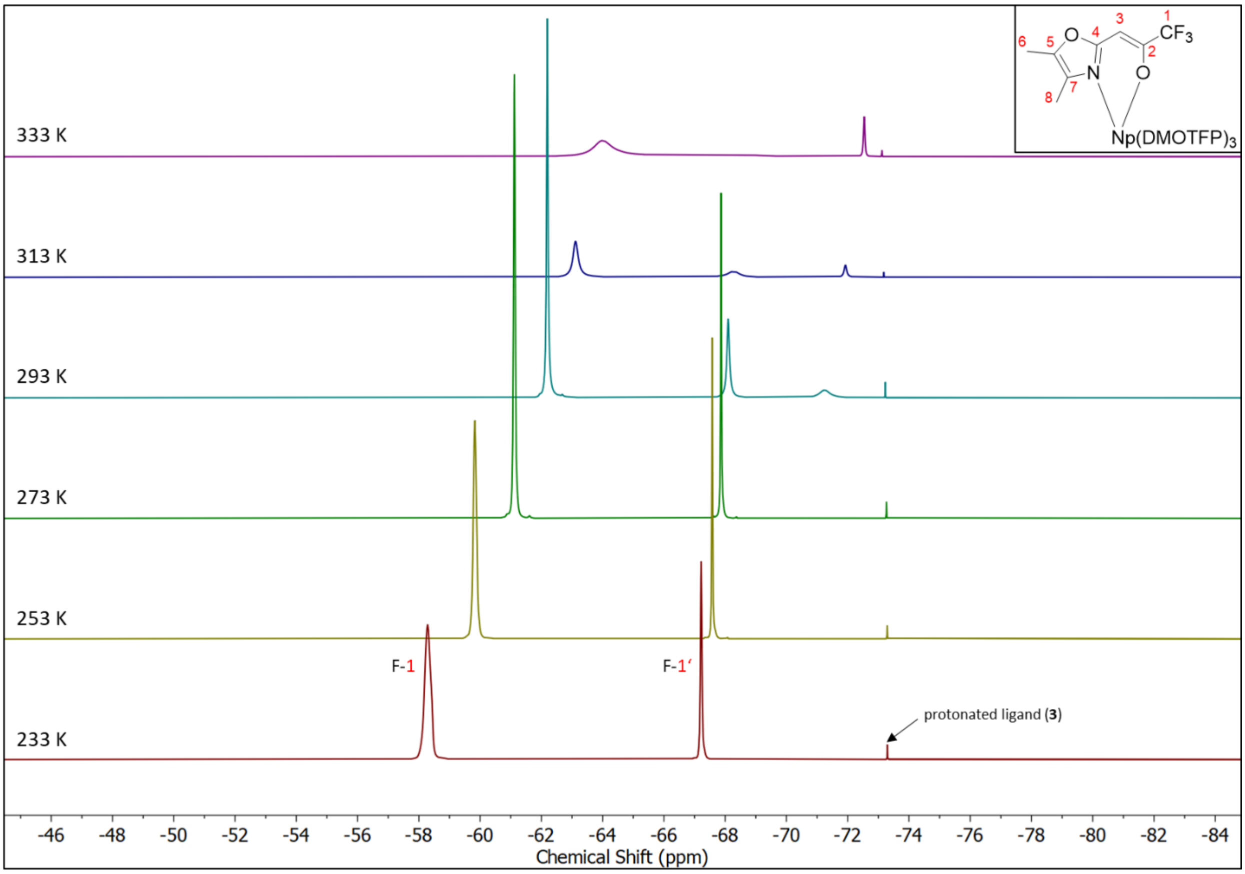

Figure S30. ^{19}F VT-NMR spectra of **Np-3** and **Np-3'** between 233 and 333K in THF-d8.

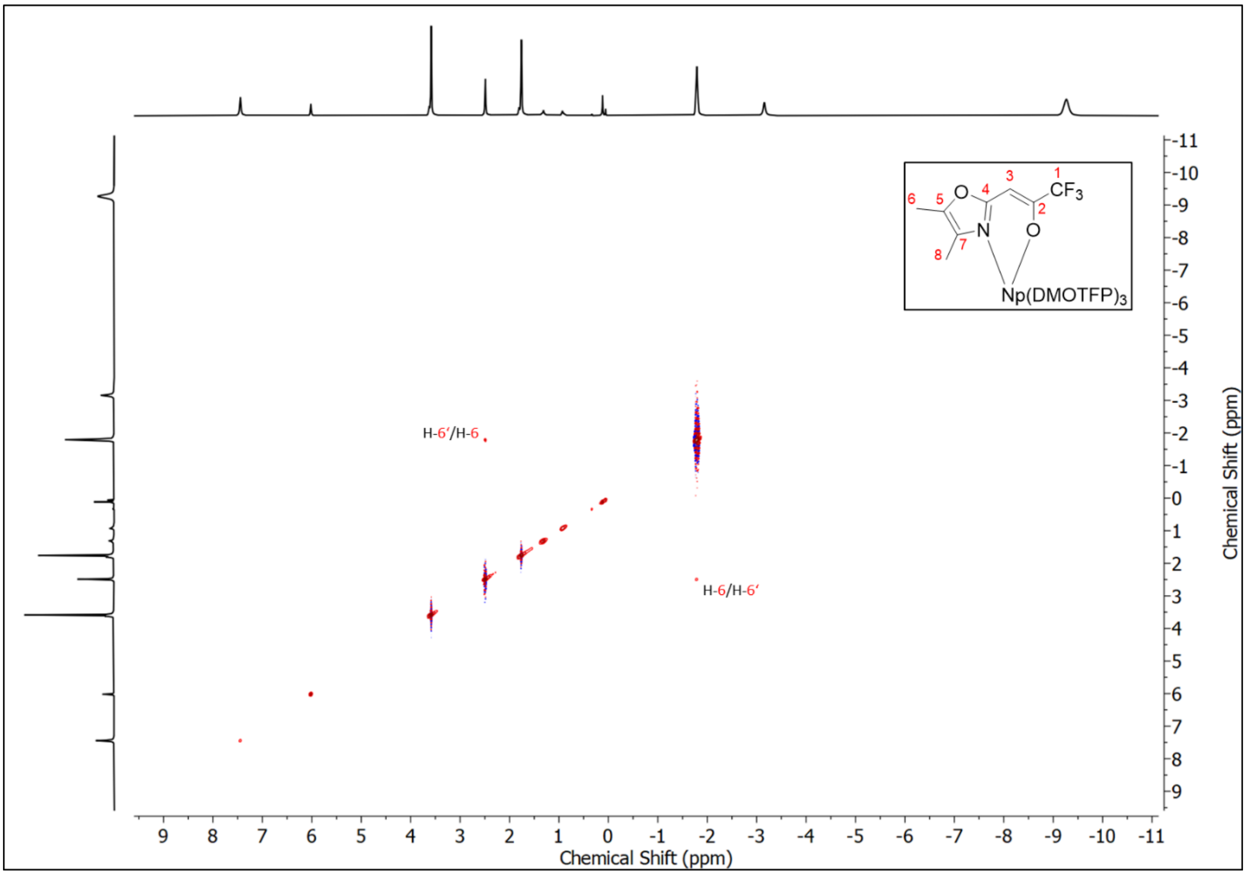

Figure S31. NOESY experiment of **Np-3** and **Np-3'** at 223K in THF-d8.

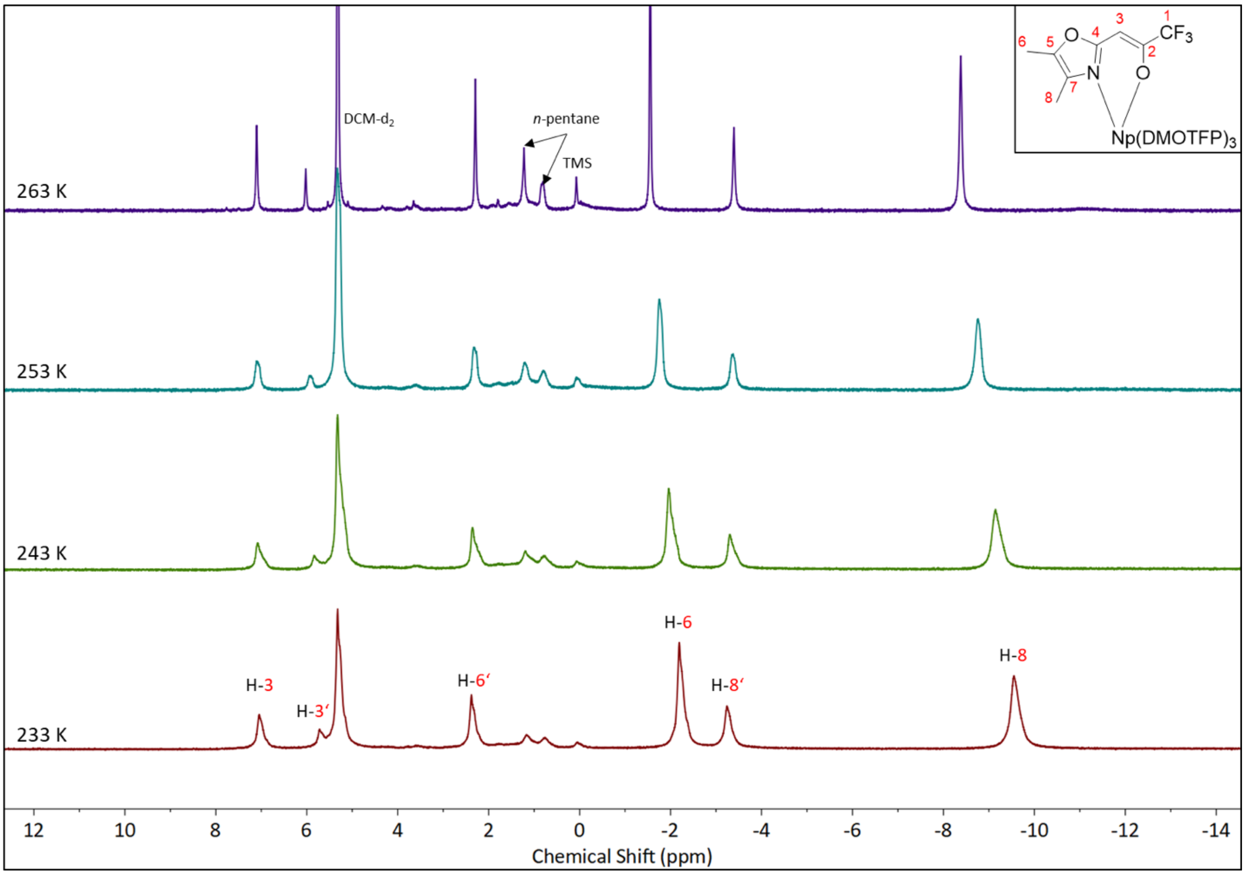

Figure S32. ^{1}H VT-NMR spectra of **Np-3** and **Np-3'** between 233 and 263K in DCM-d2. Broad signals at low temperatures due to precipitation of the complex.

[U(DMOTFP)$_4$] (U-3)

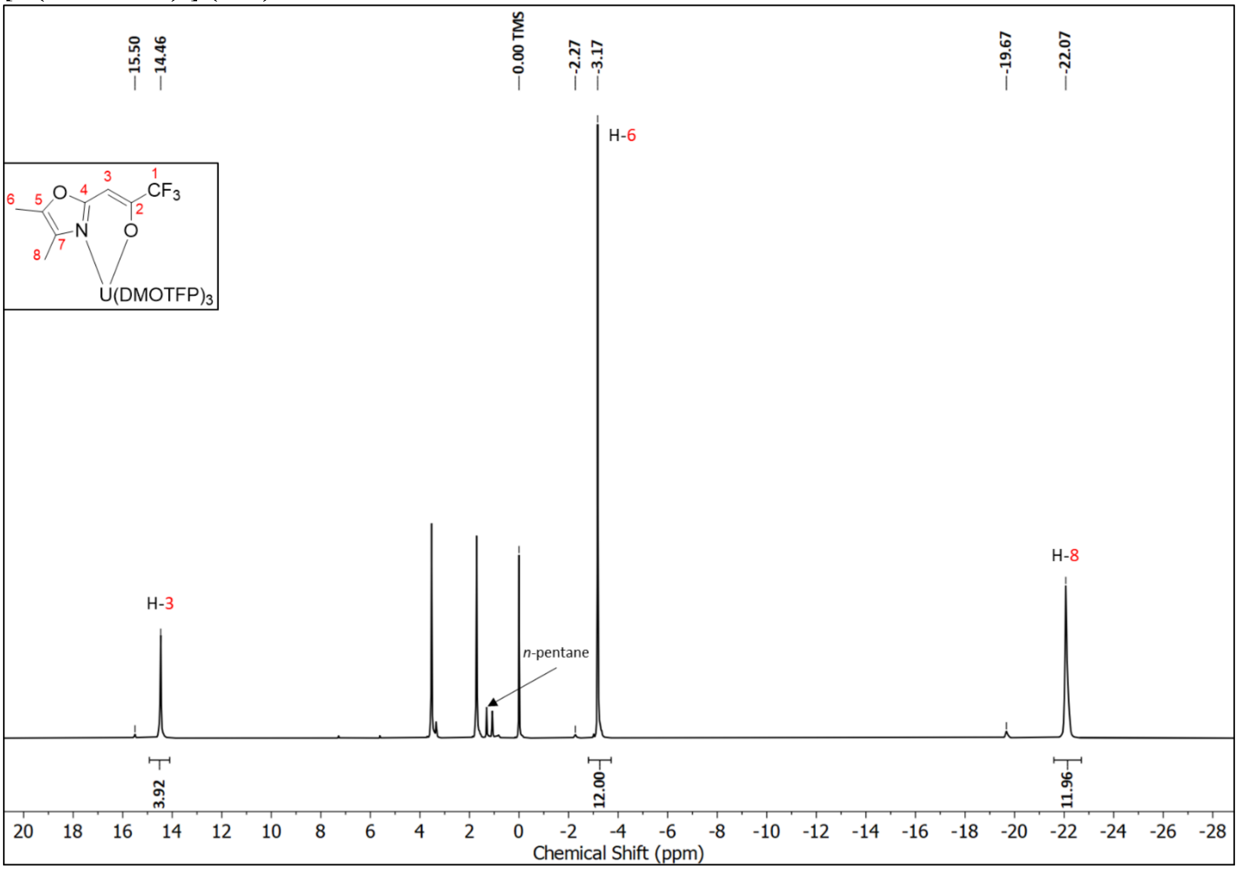

Figure S33. ^{1}H NMR spectrum of **U-3** in THF-d8 at 218K.

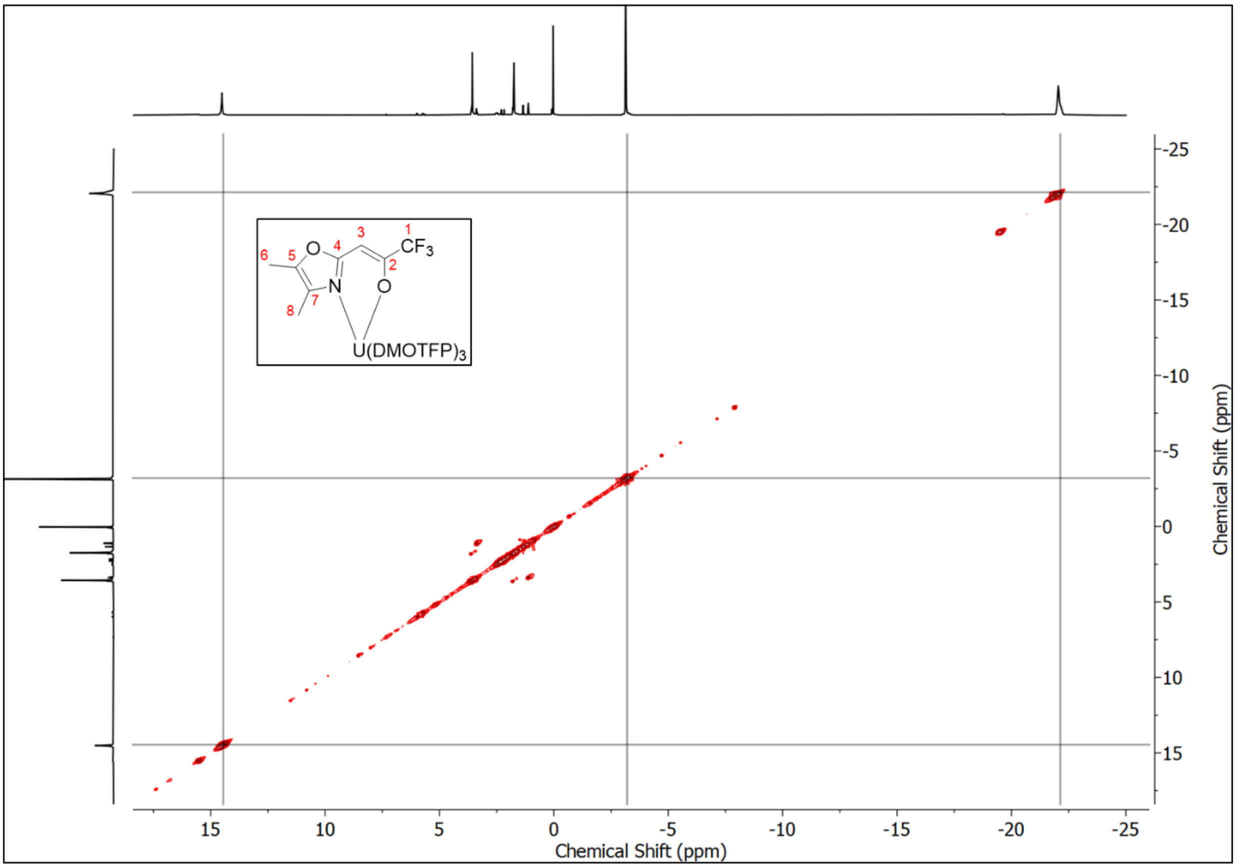

Figure S34. ^{1}H^{1}H COSY experiment of **U-3** in THF-d8 at 218K.

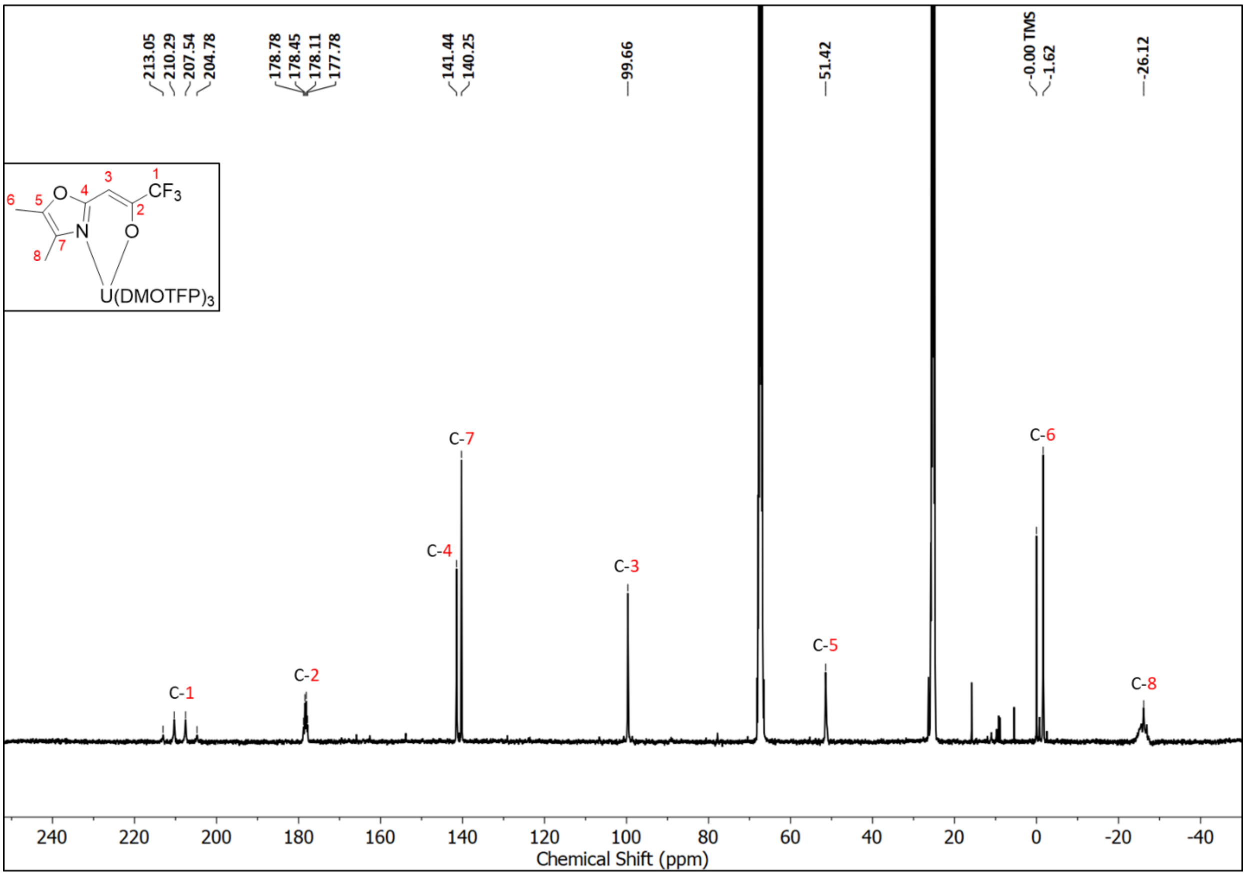

Figure S35. ^{13}C NMR spectrum of **U-3** in THF-d8 at 218K.

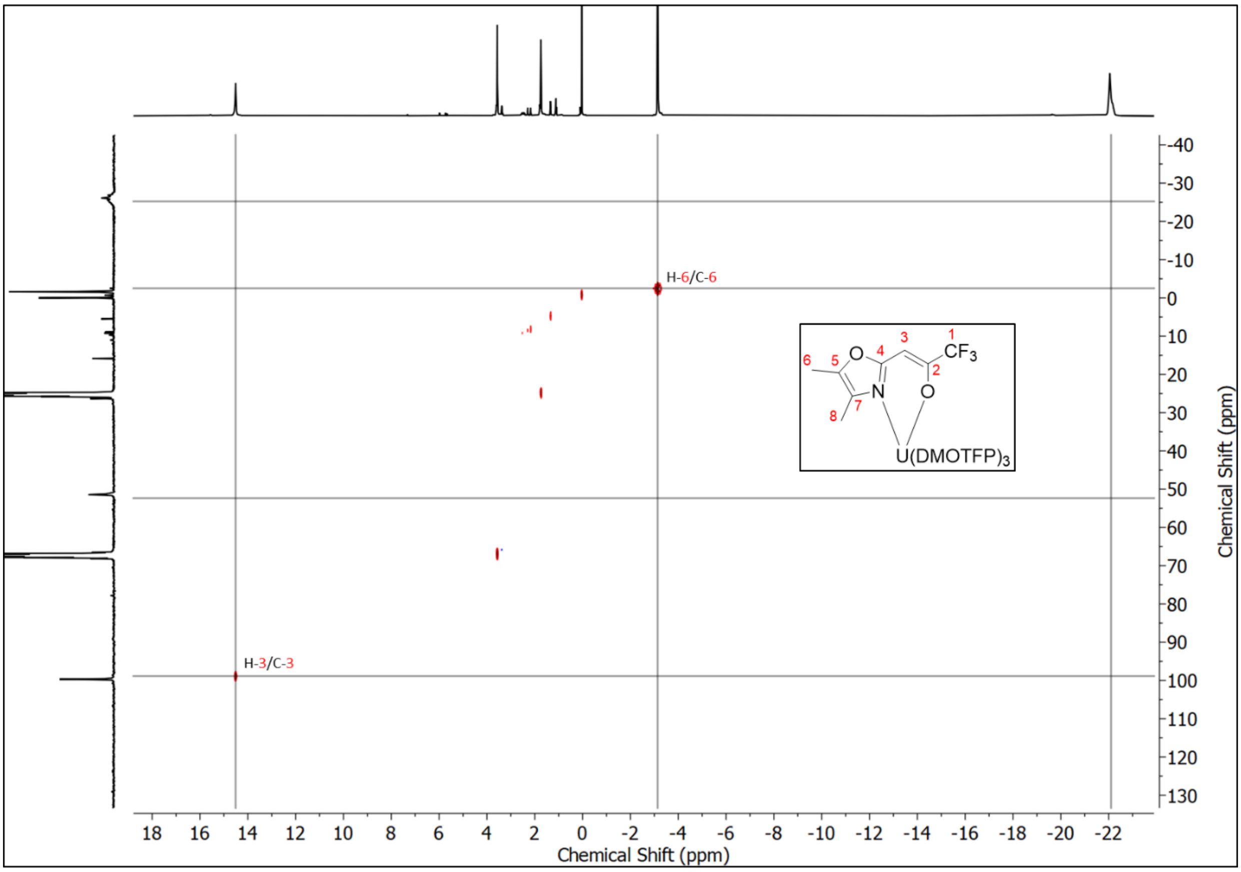

Figure S36. ^{1}H^{13}C HSQC experiment of **U-3** in THF-d8 at 218K.

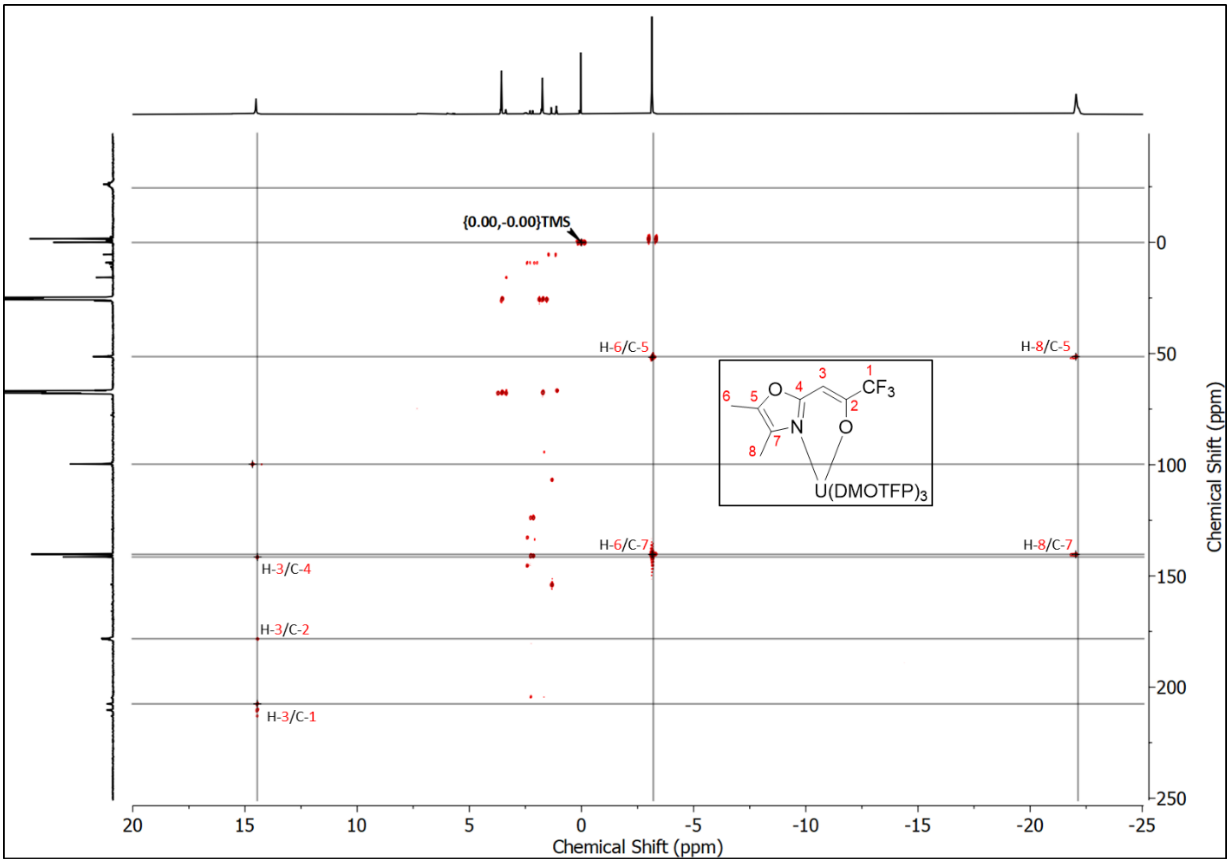

Figure S37. $^{1}H^{13}C$ HMBC experiment of **U-3** in THF-d8 at 218K.

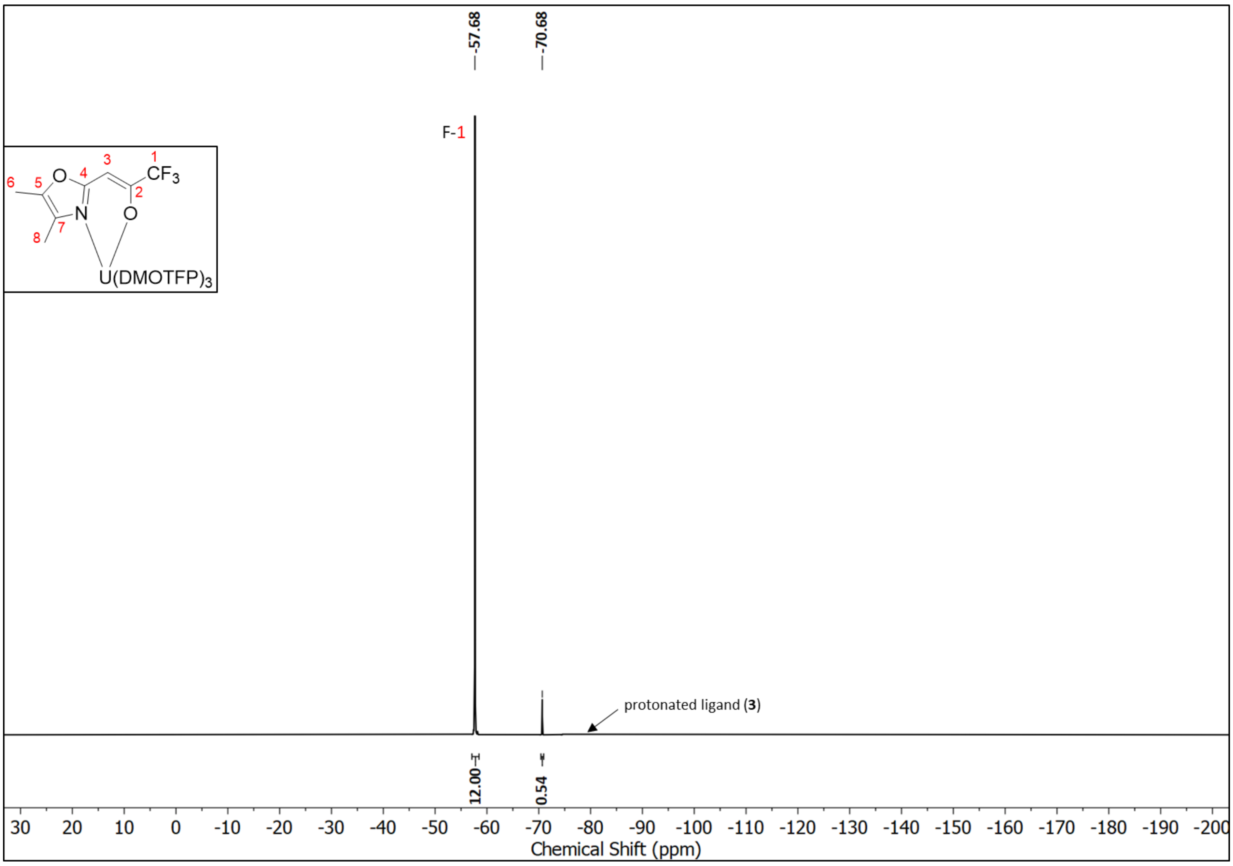

Figure S38. ^{19}F NMR spectrum of **U-3** in THF-d8 at 218K.

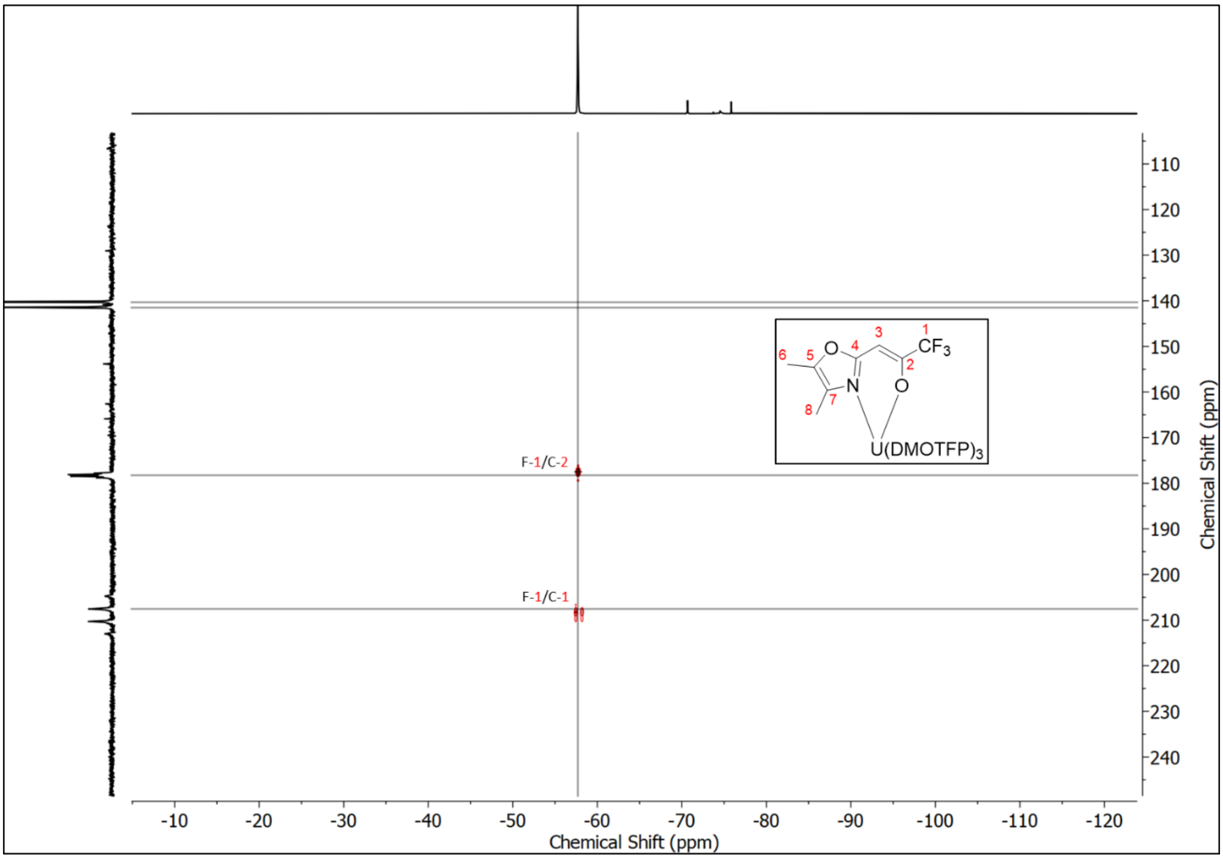

Figure S39. $^{19}F^{13}C$ HMBC experiment of **U-3** in THF-d8 at 218K.

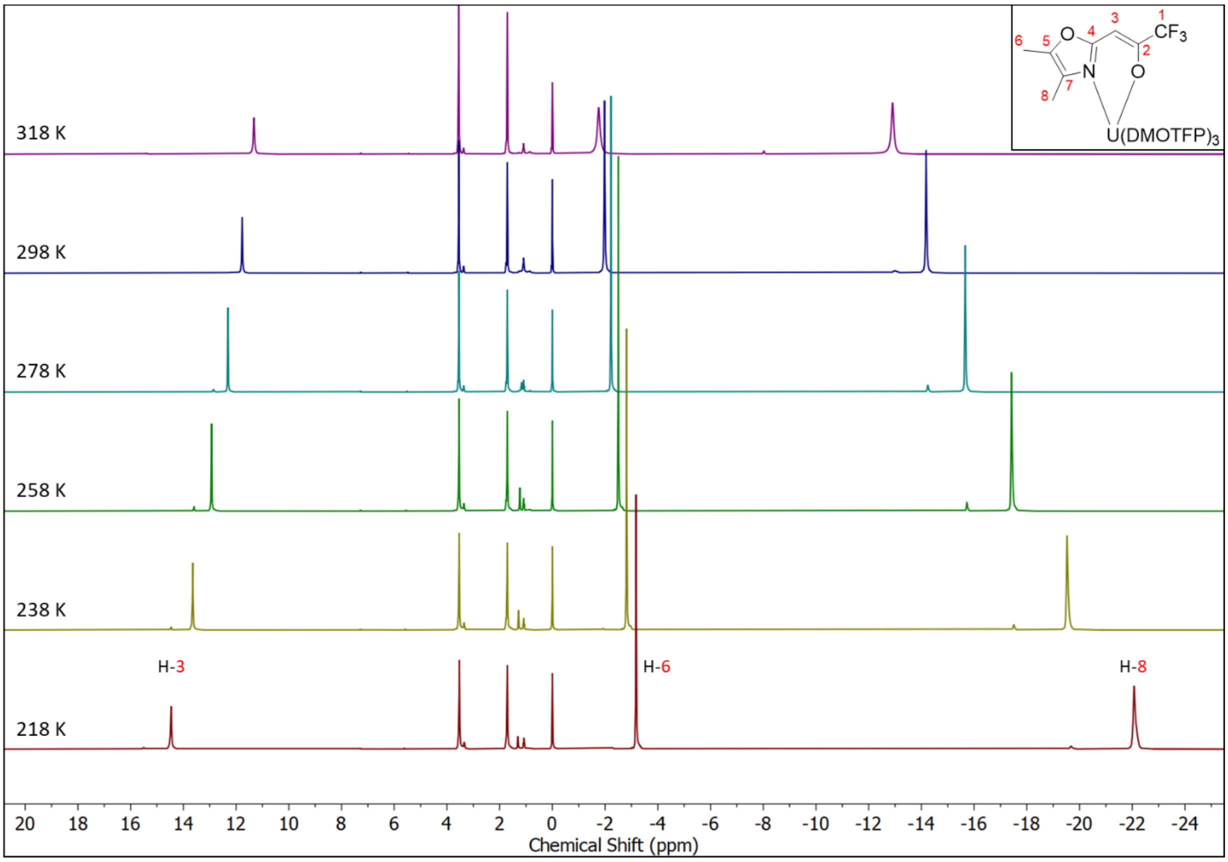

Figure S40. ^{1}H VT-NMR spectra of **U-3** between 218 and 318K in THF-d8.

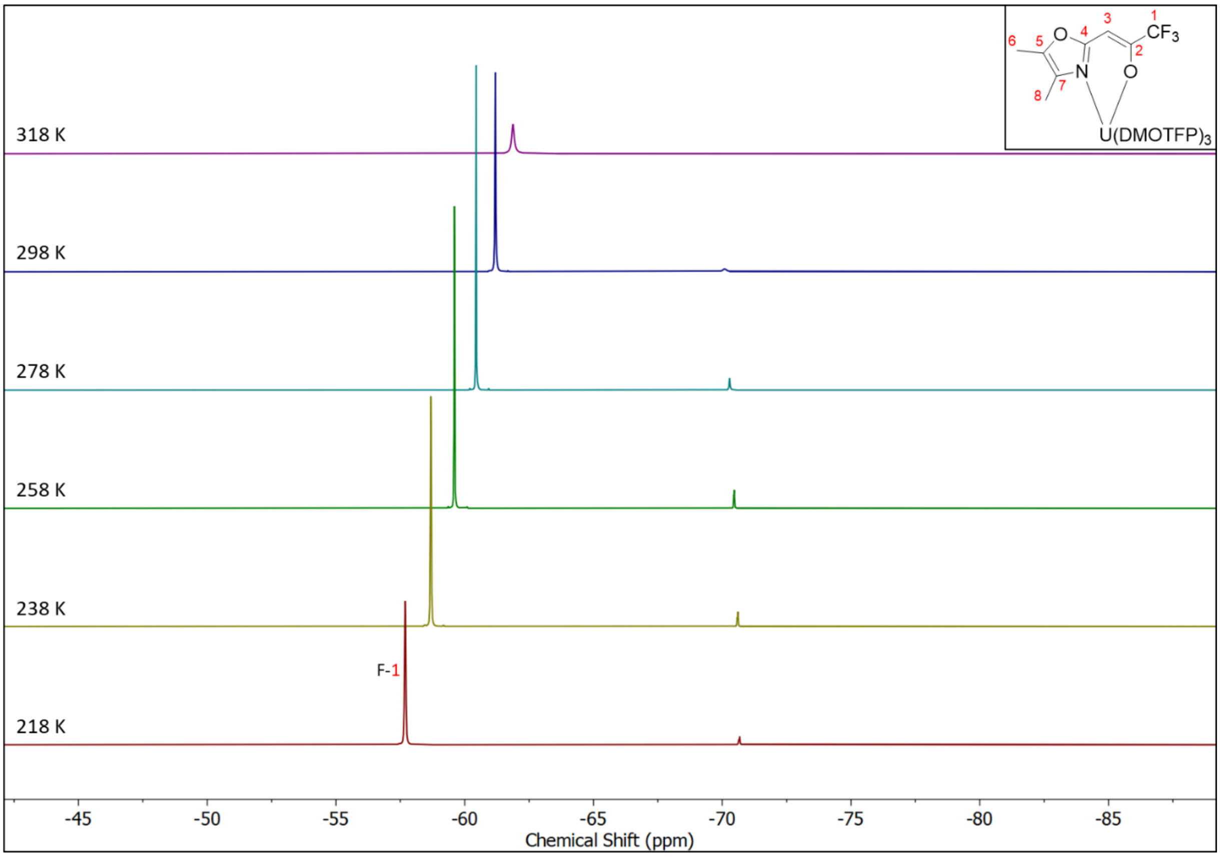

Figure S41. ^{19}F VT-NMR spectra of **U-3** between 218 and 318K in THF-d8.

APCI-MS Spectra

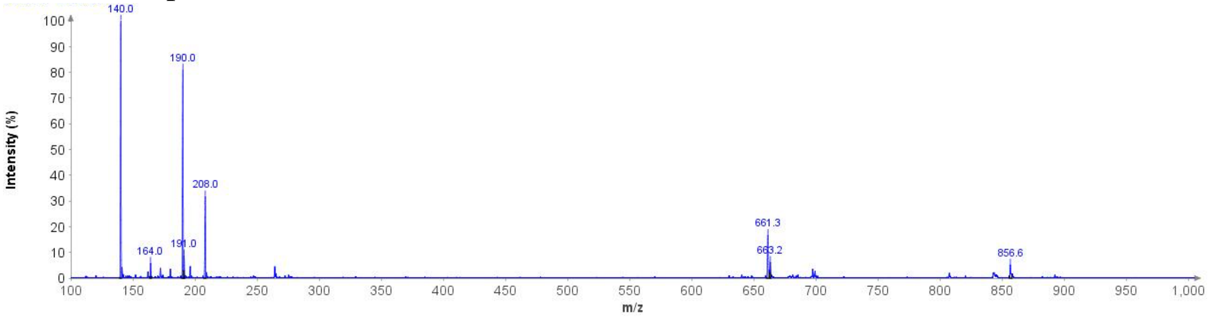

Figure S42. APCI-MS spectrum of **U-1** in positive mode at 200 °C.

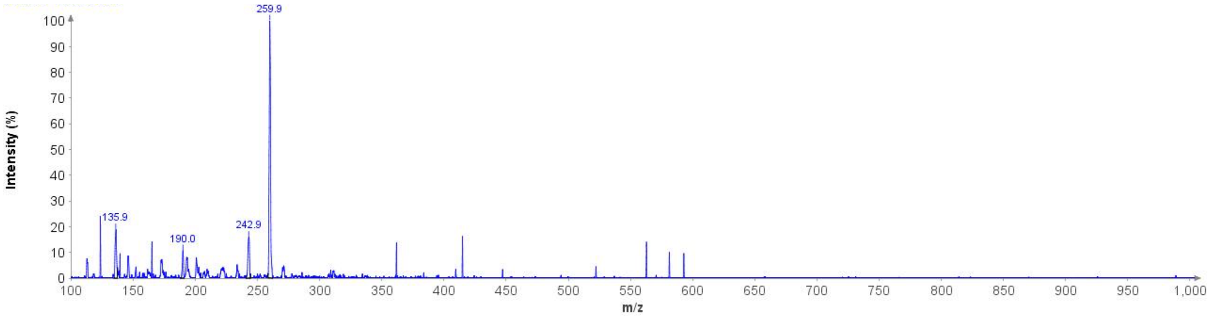

Figure S43. APCI-MS spectrum of **U-1** in negative mode at 200 °C.

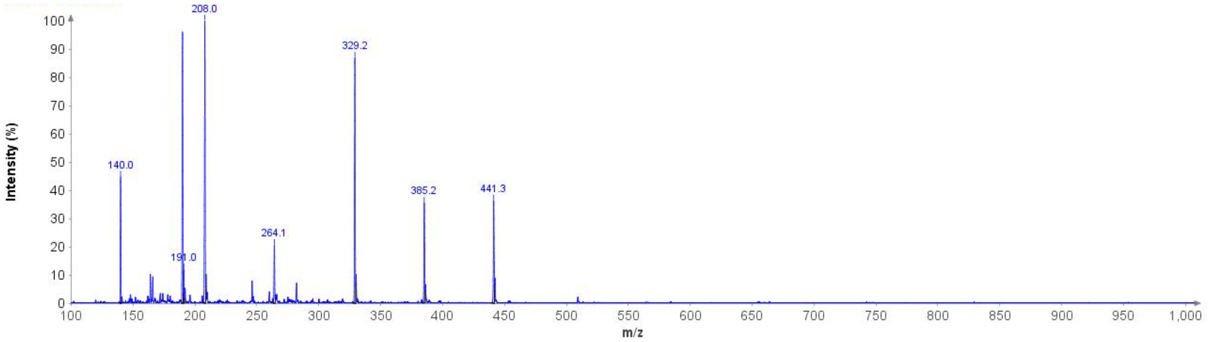

Figure S44. APCI-MS spectrum of **Np-1** in positive mode at 200 °C.

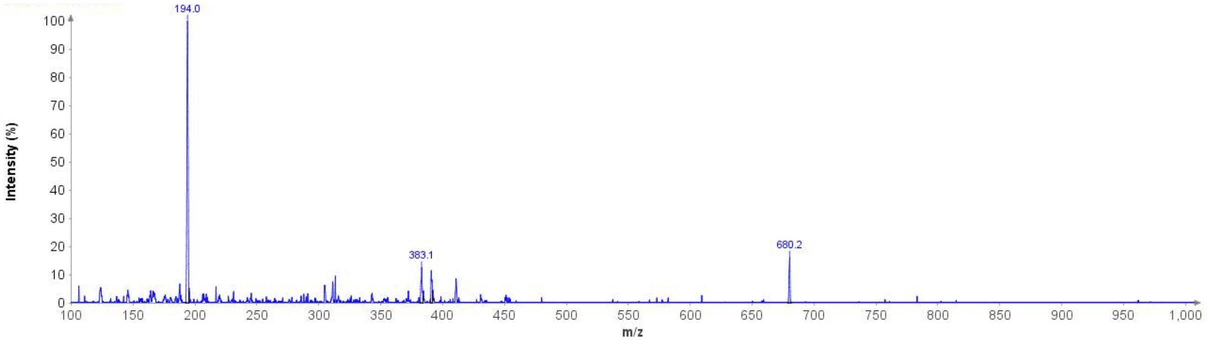

Figure S45. APCI-MS spectrum of **Np-1** in negative mode at 200 °C.

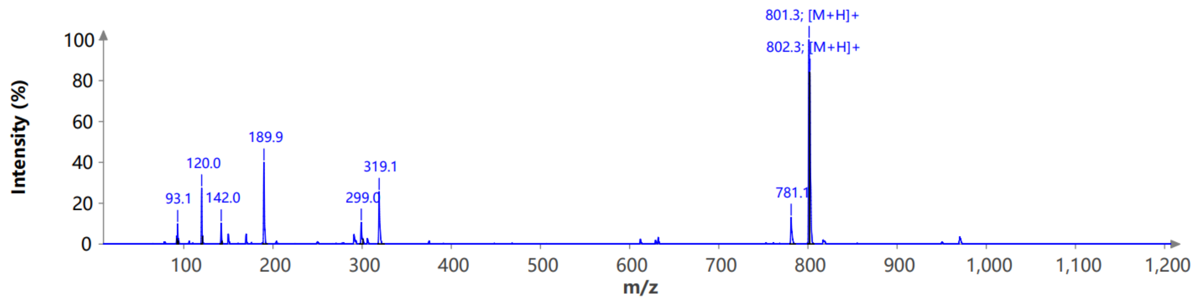

Figure S46. APCI-MS spectrum of **Np-2** in positive mode at 200 °C.

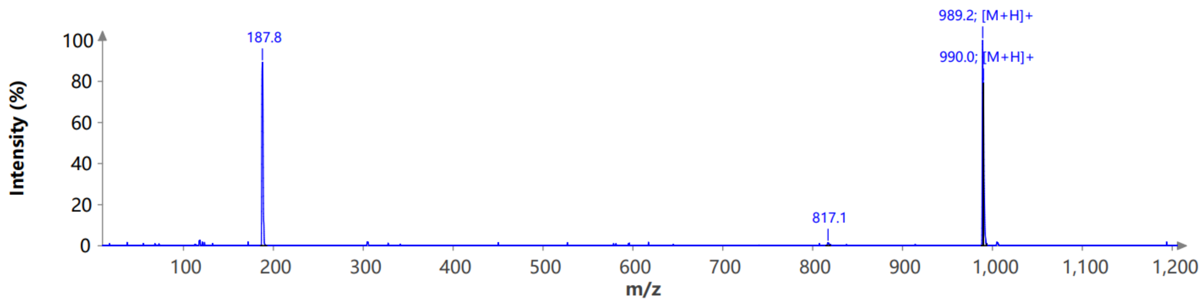

Figure S47. APCI-MS spectrum of **Np-2** in negative mode at 200 °C.

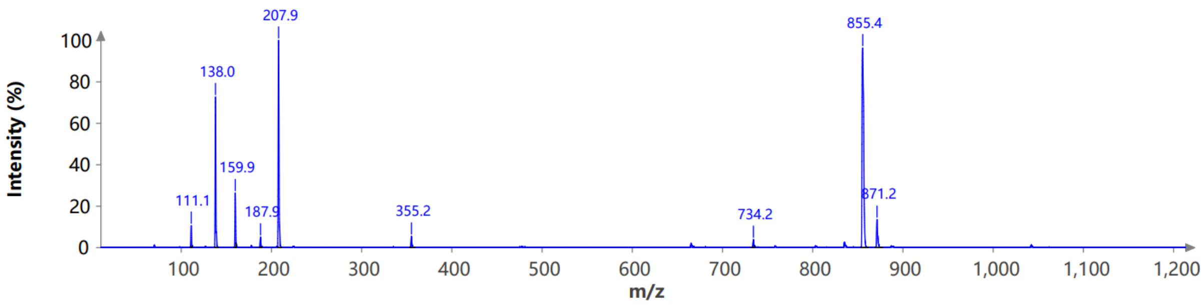

Figure S48. APCI-MS spectrum of **Np-3** in positive mode at 200 °C.

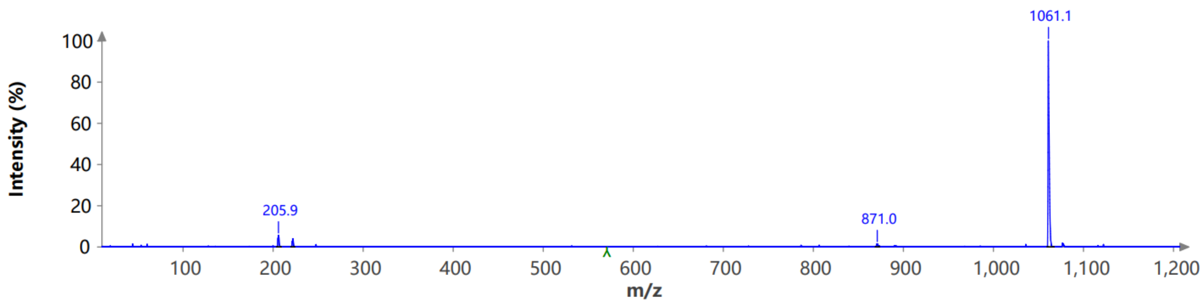

Figure S49. APCI-MS spectrum of **Np-3** in negative mode at 200 °C.

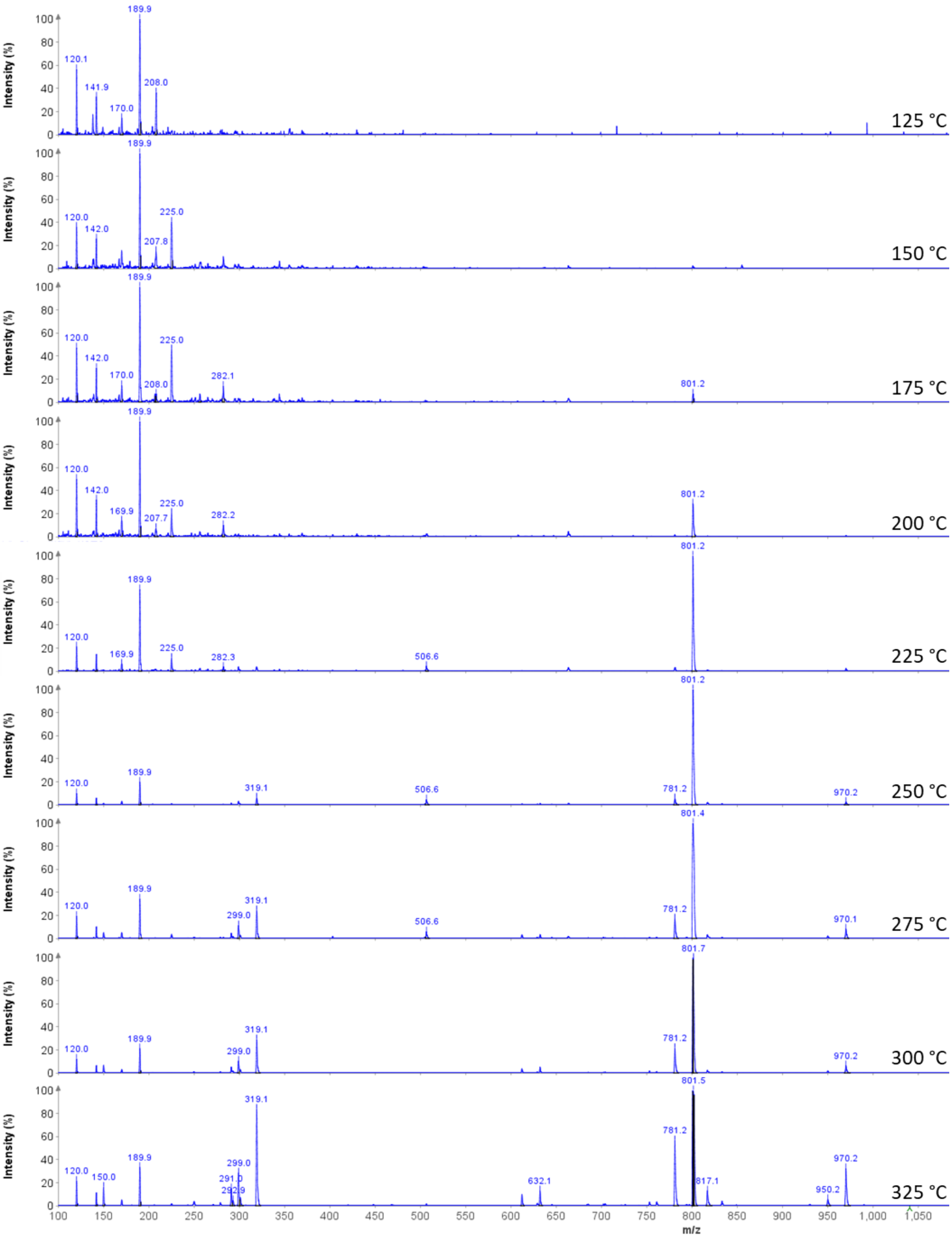

Figure S50. APCI-MS spectrum of **Np-2** in positive mode at 125 °C to 325 °C.

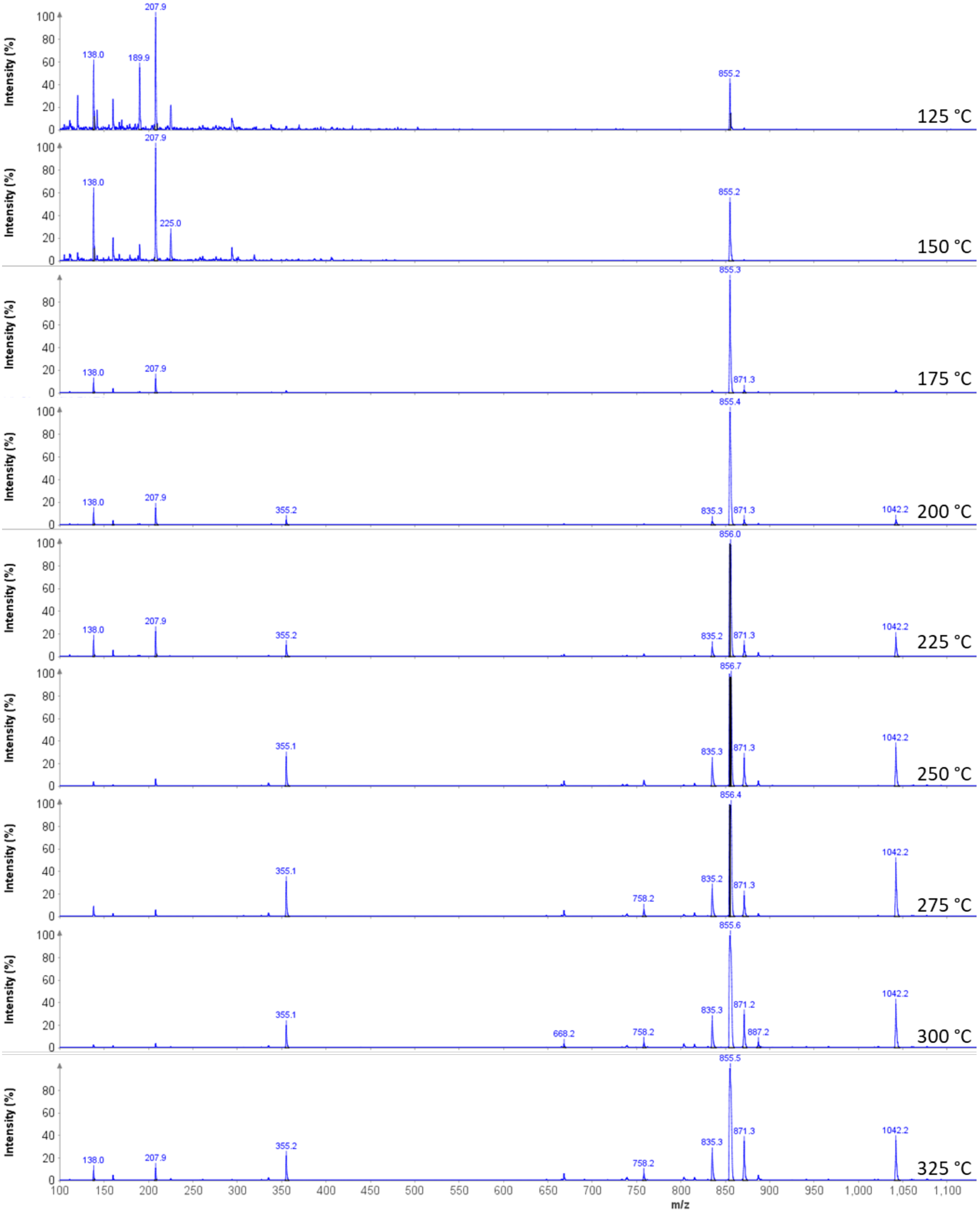

Figure S51. APCI-MS spectrum of **Np-3** in positive mode at 125 °C to 325 °C.

Crystallographic details

Table S1. Crystallographic data for [Np(PyTFP)$_4$] **Np-2** and [Np(DMOTFP)$_4$] **Np-3**.

**Complex**	**Np-2 (CCDC Number: 2370145)**	**Np-3 (CCDC Number: 2370143)**
Empirical formula	$C_{32}H_{20}F_{12}N_4NpO_4$	$C_{32}H_{28}F_{12}N_4NpO_8$
Formula weight	989.52	1061.58
Temperature/K	100.0	100.0
Crystal system	orthorhombic	monoclinic
Space group	Pbca	C2/c
a/Å	10.1300(7)	11.0763(11)
b/Å	20.2718(14)	18.9463(16)
c/Å	31.548(2)	18.2674(17)
α/°	90	90
β/°	90	105.210(4)
γ/°	90	90
Volume/Å^3	6478.4(8)	3699.2(6)
Z	8	4
ρ_{calc}g/cm^3	2.029	1.906
μ/mm^{-1}	3.323	2.924
F(000)	3784.0	2052.0
Crystal size/mm^3	0.25 × 0.098 × 0.059	0.244 × 0.2 × 0.14
Radiation	MoK$_\alpha$ (λ = 0.71073)	MoK$_\alpha$ (λ = 0.71073)
2Θ range for data collection/°	4.22 to 63.008	4.3 to 75.712
Index ranges	$-9 \leq h \leq 14$, $-29 \leq k \leq 22$, $-46 \leq l \leq 45$	$-19 \leq h \leq 18$, $-32 \leq k \leq 32$, $-31 \leq l \leq 31$
Reflections collected	88019	101589
Independent reflections	10758 [R_{int} = 0.0350, R_{sigma} = 0.0211]	9940 [R_{int} = 0.0454, R_{sigma} = 0.0217]
Data/restraints/parameters	10758/0/478	9940/0/276
Goodness-of-fit on F^2	1.269	1.102
Final R indexes [I≥2σ (I)]	R_1 = 0.0325, wR_2 = 0.0569	R_1 = 0.0200, wR_2 = 0.0422
Final R indexes [all data]	R_1 = 0.0399, wR_2 = 0.0589	R_1 = 0.0230, wR_2 = 0.0435
Largest diff. peak/hole / e Å^{-3}	1.52/-2.17	0.86/-1.04

Table S2. Crystallographic data for [U(DMOTFP)$_4$] **U-3** and [UCl_2(TFB-*t*BuA)$_2$(THF)] **U-1**.

**Complex**	**U-3 (CCDC Number: 2370142)**	**U-1 (CCDC Number: 2370144)**
Empirical formula	$C_{32}H_{28}F_{12}N_4UO_8$	$C_{20}H_{30}Cl_2F_6N_2O_3U$
Formula weight	1062.61	769.39
Temperature/K	100(2)	100(2)
Crystal system	monoclinic	orthorhombic
Space group	C2/c	Pccn
a/Å	11.3081(5)	19.0415(6)
b/Å	18.7812(8)	31.9499(9)
c/Å	17.9366(8)	8.9188(3)
α/°	90	90
β/°	104.7693(15)	90
γ/°	90	90
Volume/Å^3	3683.5(3)	5426.0(3)
Z	4	8
ρ_{calc}g/cm^3	1.916	1.884
μ/mm^{-1}	4.522	6.244
F(000)	2048.0	2944.0
Crystal size/mm^3	0.237 × 0.106 × 0.052	0.234 × 0.198 × 0.108
Radiation	MoK_α (λ = 0.71073)	MoK_α (λ = 0.71073)
2Θ range for data collection/°	4.31 to 56.588	4.382 to 54.53
Index ranges	$-15 \leq h \leq 15$, $-24 \leq k \leq 24$, $-23 \leq l \leq 23$	$-24 \leq h \leq 24$, $-41 \leq k \leq 41$, $-11 \leq l \leq 11$
Reflections collected	4566	73131
Independent reflections	4566 [R_{int} = 0.0563, R_{sigma} = 0.0217]	6078 [R_{int} = 0.0500, R_{sigma} = 0.0274]
Data/restraints/parameters	4566/37/303	6078/82/419
Goodness-of-fit on F^2	1.292	1.069
Final R indexes [I≥2σ (I)]	R_1 = 0.0284, wR_2 = 0.0626	R_1 = 0.0393, wR_2 = 0.0685
Final R indexes [all data]	R_1 = 0.0351, wR_2 = 0.0642	R_1 = 0.0623, wR_2 = 0.0731
Largest diff. peak/hole / e Å^{-3}	0.99/-1.71	1.41/-1.74

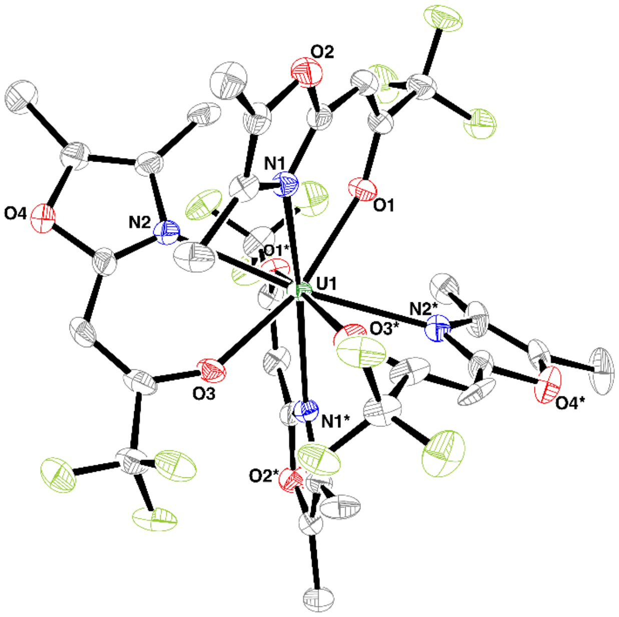

Figure S52. Molecular structure of **U-3**. Hydrogen atoms are omitted for clarity and ellipsoids are drawn at 50% probability level. The asymmetric unit of **U-3** comprises one half molecule of **U-3** (its atom U1 is located on a crystallographic two-fold rotation axis). Symmetry equivalent atom labels are asterisked. Selected bond lengths (Å): U1—O1 = 2.240(3), U1—O3 = 2.252(3), U1—N1 = 2.669(3), U1—N2 = 2.634(3).

Quantum Chemical Calculations

The geometry optimizations were carried out with ORCA 5.0.4[10] using the unrestricted PBE0 functional with a relativistically recontracted Karlsruhe basis sets ZORA-def2- TZVPP[11,12] for all atoms, the scalar relativistic ZORA Hamiltonian[13,14], atom-pairwise dispersion correction with the Becke–Johnson damping scheme (D3BJ)[15,16] and COSMO solvation (toluene, $\varepsilon = 2.94$, rsolv = 8.9). VeryTightSCF and slowconv options were applied with a radial integration accuracy of 10 for Np for all calculations. Calculations were started from the molecular structure obtained by single-crystal X-ray diffraction analysis and an isomer were generated therefrom. Numerical frequency calculations were performed to prove convergence at the local minimum after geometry optimization and to obtain the final Gibbs free energy (293.15 K). Graphics were generated using ChemCraft.[17]

Atomic coordinates and total energies from DFT calculations:

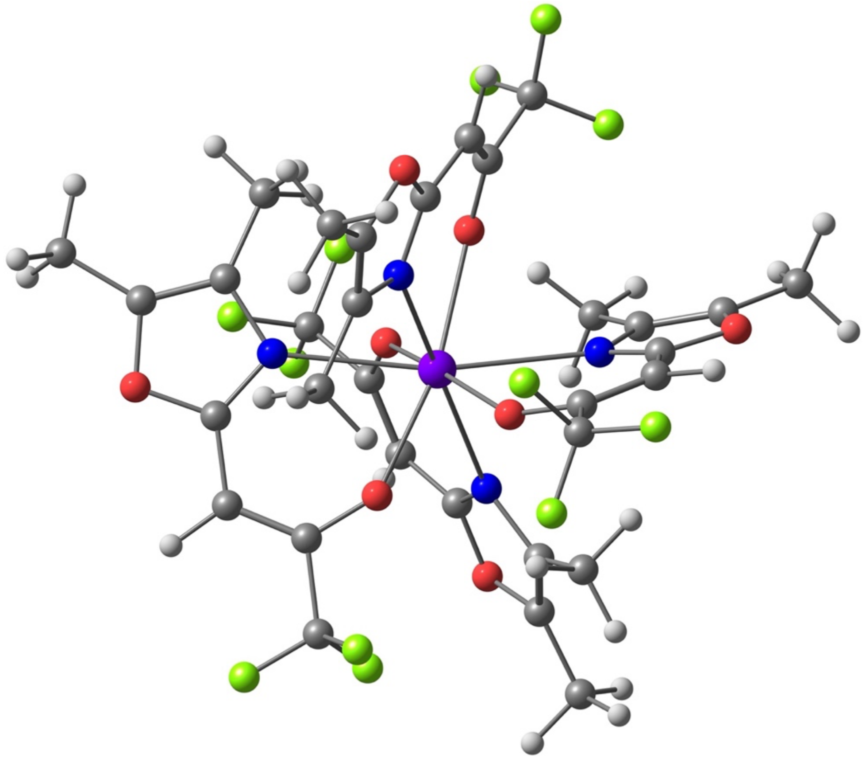

Figure S53. Optimized molecular structure of **Np-3**.

PBE0:

final single point energy: -33541.289497413112 a.u.
final Gibbs free energy: -33540.78522511 a.u.

Table S3. Atomic coordinates for optimized structure of **Np-3**.

Np	0.04365858168758	0.00011506872784	-0.00006584758474
F	-1.96814303408606	-0.27407107952397	-4.49505871025547
F	-3.15533997060020	1.14267735579733	-3.39062530838918
F	-4.00299836930244	-0.75355624528993	-3.96249034220487
F	2.24129900820728	-0.20382416028428	4.41524538514600
F	3.44723331121050	1.08522013429687	3.18174463146539
F	4.21736361660417	-0.82557053252494	3.81620894701447
O	-1.25121507645366	-0.24847844305265	-1.84083223441121
O	-3.26123847178657	-3.34290599587319	0.11760261834326
O	1.42464478019125	-0.24238517307991	1.76974485905241
O	3.16230806377785	-3.53227969321620	-0.11936522026932
N	-1.49281596033494	-2.04554555211438	0.28539125797000
N	1.47702628282004	-2.12741648391628	-0.29593241484809
C	-2.36913308302349	-0.75260425429980	-2.22490327508570
C	-2.88669333360809	-0.16455368259033	-3.52498559904468
C	-3.06619191050997	-1.73026021342846	-1.60213784960925
H	-3.98983798945127	-2.10959222779848	-2.00944930157976
C	-2.57171365232937	-2.32157998688274	-0.40850337572749
C	-1.47967378792884	-2.95398993517799	1.34479468005687
C	-0.43818948657013	-2.96670094073656	2.39586965089271
H	0.53423856285788	-3.24933617709876	1.99132354424050
H	-0.70117459052456	-3.67862099538273	3.17675054832483
H	-0.33617898206014	-1.97969959450726	2.84785296248887
C	-2.56819890135750	-3.74508948788741	1.23000700852817
C	2.50730865441064	-0.81578182418186	2.15441741452978

C	3.11574039278188	-0.19633020655637	3.39886719858809
C	3.10131741853190	-1.87749504246926	1.56390675514570
H	4.00577289679156	-2.30995765028067	1.96120931892692
C	2.54112582426749	-2.46260719417168	0.39666090402233
C	1.40988720308890	-3.04079656569089	-1.35012355834814
C	0.38089981255475	-2.99541726799510	-2.41208673599091
H	-0.61666028516148	-3.18164895515567	-2.01346235629174
H	0.59089381117348	-3.75124675814582	-3.16733361835990
H	0.36727754753881	-2.01768665091975	-2.89452998610818
C	2.44531266810777	-3.89898886407193	-1.22780617617941
C	2.93400282506917	-5.05506692757166	-1.99722738913930
H	3.94790019696707	-4.88062496488569	-2.36558822952528
H	2.28378991157032	-5.23350872426924	-2.85183418667706
H	2.95106370910047	-5.95873877157036	-1.38310557546791
C	-3.13343088734975	-4.85568315462006	2.01358764567885
H	-4.13165960386018	-4.60641921599503	2.38195917969122
H	-2.49480272663870	-5.06825488352412	2.86910080463538
H	-3.21510674209230	-5.76345036608856	1.41091453200825
F	2.24026545931424	0.20408997784391	-4.41576095852966
F	3.44567322882663	-1.08591084609281	-3.18275566518998
F	4.21678122915809	0.82458880271143	-3.81691229863750
F	-1.96950104718929	0.27537909612500	4.49452536988170
F	-3.15566042764863	-1.14194619244362	3.38967212735263
F	-4.00420309725313	0.75412073625454	3.96069634334837
O	1.42410374236751	0.24242744188766	-1.77013436927998
O	3.16394835656097	3.53042390820901	0.12008128251818
O	-1.25121854923357	0.24908702102540	1.84058990498834
O	-3.25982886505065	3.34448322330230	-0.11820507189787
N	1.47761788013039	2.12671762030279	0.29601083991750
N	-1.49174979461071	2.04663581368449	-0.28570876711782
C	2.50698298763782	0.81536821373116	-2.15483927593025
C	3.11487279282734	0.19588264920747	-3.39953554795761
C	3.10166135033976	1.87652411779183	-1.56402084387975
H	4.00636386744128	2.30856542036346	-1.96122138556758
C	2.54188765916343	2.46153074567871	-0.39650605450898
C	1.41122582353280	3.03961295143481	1.35067876896695
C	0.38222288230086	2.99459575344886	2.41264306921504
H	-0.61514971642650	3.18198570475168	2.01416180202948
H	0.59297008677789	3.74980206174008	3.16830228032208
H	0.36763380240125	2.01659861213231	2.89453654350317
C	2.44744406224850	3.89693207064550	1.22889883188757
C	-2.36928507595522	0.75335993285302	2.22406749471427
C	-2.88746990865034	0.16536834559113	3.52392768235057
C	-3.06588038119562	1.73125975333361	1.60117141028216
H	-3.98971159908499	2.11056323751228	2.00813718876343
C	-2.57075612146097	2.32285831465946	0.40792354541034
C	-1.47789556850434	2.95541078173254	-1.34482279218016
C	-0.43610221599825	2.96805955649270	-2.39558018117501
H	0.53628047863373	3.25047574533834	-1.99071168033833
H	-0.69874642364540	3.68009018341820	-3.17647334944262
H	-0.33407780455502	1.98110387013672	-2.84763698298844
C	-2.56626828451232	3.74675779433154	-1.23025862173098
C	-3.13095292260815	4.85764391289066	-2.01382558761469
H	-4.12901910998861	4.60862295171853	-2.38280189177312
H	-2.49184294151289	5.07039735691448	-2.86893489583177
H	-3.21283157403306	5.76523830307312	-1.41091889978375

C	2.93723846757362	5.05203165923387	1.99909497086035
H	3.95080895348055	4.87622673456767	2.36771744111737
H	2.28694372978439	5.23079115428834	2.85357311451649
H	2.95555935433542	5.95599479218580	1.38544355375740

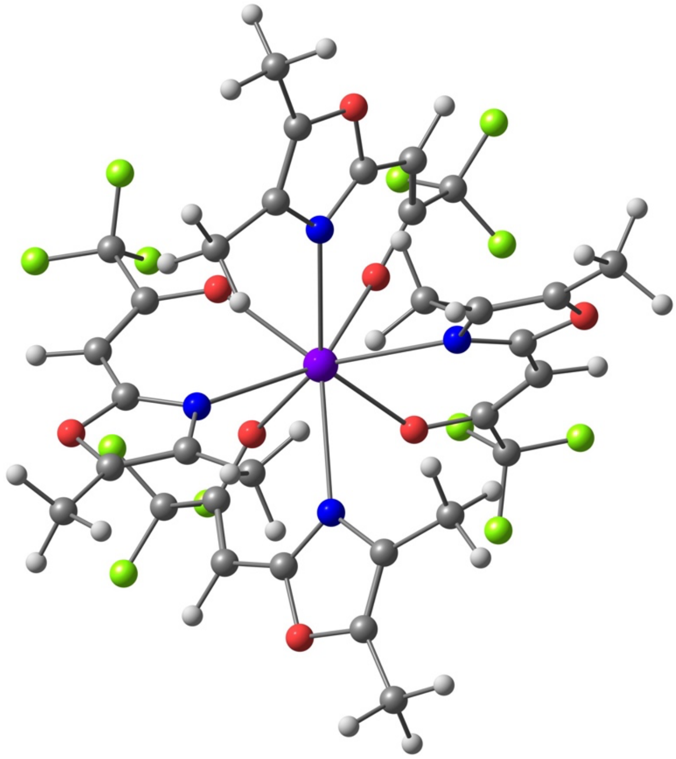

Figure S54. Optimized molecular structure of **Np-3'**.

PBE0:

final single point energy: -33541.262886263292 a.u.

final Gibbs free energy: -33540.75637037 a.u.

Table S4. Atomic coordinates for optimized structure of **Np-3'**.

Np	0.00415797060777	0.00845524879321	0.21220876492296
F	0.36826999889352	4.92986097661917	-2.48783281110044
F	0.76243254314194	2.96332404315814	-3.27896029147598
F	2.21602490657755	3.92370243092949	-2.01311132120868
F	-3.08570628400182	0.94574850516229	-3.57282903485579
F	-2.47528398358976	2.84151958566690	-2.76035718966476
F	-4.49839904233418	2.16065129389961	-2.48462400474711
O	0.78307535455375	1.94341762147845	-0.69534397355556
O	-2.00642892388408	4.02010521236407	1.63579034461547
O	-1.54936293566307	0.72211301226108	-1.22836924932247
O	-4.52738144708633	-0.65030197817408	1.40373789902704
N	-1.07210535517410	2.02669992579486	1.49299418971503
N	-2.33441981813676	-0.67543637060162	1.19979109341755
C	0.27467346290470	3.08381755847999	-0.97650467713096
C	0.89946097964303	3.73921463705001	-2.19609961855233
C	-0.69825779562088	3.72832807934580	-0.29050198703782
H	-1.04022234917662	4.70445324544585	-0.59545966162014
C	-1.24230626077621	3.19416945842267	0.90539791695135
C	-1.74393911185331	2.12837146561753	2.71560545572875
C	-1.77176510410715	1.07070098504954	3.75338569429136
H	-2.64542052838414	0.42477586027190	3.66253936937237
H	-1.78744826454809	1.51996632964204	4.74649952167458

H	-0.88901252878683	0.43822635065682	3.68883029648702
C	-2.31587030709172	3.34793336947242	2.78600242626602
C	-2.79426663690561	1.04256752868779	-1.24631895558647
C	-3.22329708856478	1.76137383704288	-2.51597233340581
C	-3.72528669683846	0.73584777151931	-0.31396007016702
H	-4.75095986735119	1.04244661244962	-0.44600339323258
C	-3.45596966678635	-0.15194578882200	0.76523631810972
C	-2.70980202482073	-1.62402960247641	2.15354861647888
C	-1.75676908054561	-2.50080938581113	2.86699910412124
H	-1.24922399034586	-3.17173197341743	2.17306317285598
H	-2.28521834713211	-3.10550603061666	3.60252938526219
H	-0.99156778374018	-1.92553849423700	3.38829162198250
C	-4.05418632119773	-1.58898884789188	2.27924769563729
C	-5.04167418492428	-2.29736131371394	3.11053609552973
H	-5.60786950759103	-1.59631762401378	3.72872842258395
H	-4.53781425436247	-3.00451579596016	3.76693158198106
H	-5.75371549993516	-2.84892999821724	2.49202697115456
C	-3.15152925518950	4.04026429280502	3.78041162830245
H	-2.70783065797410	4.99513639877214	4.07155042169965
H	-3.25693243152377	3.42053310769614	4.66950367167480
H	-4.15016756002230	4.24185050729168	3.38444202178745
F	-0.41098840636924	-4.90094560898582	-2.52348036862139
F	-0.77688906613918	-2.92801800676466	-3.31204180374533
F	-2.24371654040534	-3.86899312239920	-2.04668922807247
F	3.11552089201665	-0.92029125760990	-3.55316597654528
F	2.49244481443812	-2.81675039429127	-2.75226140784932
F	4.51760191055006	-2.14689223818226	-2.46426341957688
O	-0.78479231439535	-1.91173437404034	-0.72758442639211
O	1.98657182971712	-4.03609076073724	1.59262280790695
O	1.57251252271074	-0.69266503208199	-1.21500913017794
O	4.53293226047703	0.64691577475166	1.44060642650468
N	1.07407544718565	-2.03164538737180	1.46575456723800
N	2.34203445306852	0.68438466735040	1.21462029189269
C	-0.29033356122640	-3.05750733190032	-1.00941275417421
C	-0.92466639139629	-3.70303650922279	-2.22980181582936
C	0.67568103616529	-3.71694937436452	-0.32738777725127
H	1.00457611286361	-4.69582159387766	-0.63785986444467
C	1.22951508114145	-3.19675279258255	0.86997480712739
C	1.74821165359471	-2.14880463336368	2.68581749715894
C	1.79209209975156	-1.09866705841309	3.73096120224546
H	2.67727980044847	-0.46763314092362	3.64700200961543
H	1.79754661821949	-1.55503589405651	4.72098360568869
H	0.92127277144837	-0.44962614277212	3.66929268149957
C	2.30700044510917	-3.37494796346694	2.74630194428721
C	2.81423200397789	-1.02638178912750	-1.22863678863321
C	3.24458133265582	-1.74143010943337	-2.49957048696090
C	3.74039233818570	-0.73090665487274	-0.28833847461149
H	4.76526638836585	-1.04327169258534	-0.41272208348576
C	3.46529708145715	0.15578018279758	0.79046273189352
C	2.71308360423130	1.62830456659650	2.17463031007388
C	1.75795018756861	2.50681708166639	2.88285958385926
H	1.24882834530850	3.17245128866152	2.18509724637934
H	2.28506743592796	3.11664090517381	3.61510715751172
H	0.99428406157723	1.93246817979426	3.40748096783302
C	4.05598273402842	1.58631357368578	2.31338511361110
C	5.03889572292854	2.28811395332838	3.15560449613781

H	5.59138405262001	1.58397305384454	3.78264617827106
H	4.53271200436761	3.00105942767520	3.80388795826209
H	5.76324373705392	2.83214758026241	2.54477969539243
C	3.13880235832966	-4.08251529464607	3.73322006470986
H	2.68645003597960	-5.03484227042214	4.01934606142948
H	3.25320495575496	-3.46996610929171	4.62618932908773
H	4.13417383035114	-4.29141174569436	3.33280394178696

References

(1) *APEX4, Version 2021.4-0*; Bruker AXS Inc.: Madison, Wisconsin, USA, 2021.

(2) *SADABS*; Bruker AXS Inc.: Madison, Wisconsin, USA, 2016.

(3) Sheldrick, G. M. SHELXT – Integrated Space-Group and Crystal-Structure Determination. *Acta Crystallogr. Sect. Found. Adv.* **2015**, *71* (1), 3–8. https://doi.org/10.1107/S2053273314026370.

(4) Sheldrick, G. M. Crystal Structure Refinement with SHELXL. *Acta Crystallogr. Sect. C Struct. Chem.* **2015**, *71* (1), 3–8. https://doi.org/10.1107/S2053229614024218.

(5) Reilly, S. D.; Brown, J. L.; Scott, B. L.; Gaunt, A. J. Synthesis and Characterization of $NpCl_4(DME)_2$ and $PuCl_4(DME)_2$ Neutral Transuranic An(IV) Starting Materials. *Dalton Trans.* **2013**, *43* (4), 1498–1501. https://doi.org/10.1039/C3DT53058B.

(6) Whitefoot, M. A.; Perales, D.; Zeller, M.; Bart, S. C. Synthesis of Non-Aqueous Neptunium(III) Halide Solvates from NpO_2. *Chem. – Eur. J.* **2021**, *27* (72), 18054–18057. https://doi.org/10.1002/chem.202103265.

(7) Vanagas, N. A.; Wacker, J. N.; Rom, C. L.; Glass, E. N.; Colliard, I.; Qiao, Y.; Bertke, J. A.; Van Keuren, E.; Schelter, E. J.; Nyman, M.; Knope, K. E. Solution and Solid State Structural Chemistry of Th(IV) and U(IV) 4-Hydroxybenzoates. *Inorg. Chem.* **2018**, *57* (12), 7259–7269. https://doi.org/10.1021/acs.inorgchem.8b00919.

(8) Kawase, M.; Teshima, M.; Saito, S.; Tani, S. Trifluoroacetylation of Methylpyridines and Other Methylazines: A Convenient Access to Trifluoroacetonylazines. *Heterocycles* **1998**, *48* (10), 2103.

(9) Appel, L.; Leduc, J.; Webster, C. L.; Ziller, J. W.; Evans, W. J.; Mathur, S. Synthesis of Air-Stable, Volatile Uranium(IV) and (VI) Compounds and Their Gas-Phase Conversion To Uranium Oxide Films. *Angew. Chem. Int. Ed.* **2015**, *54* (7), 2209–2213. https://doi.org/10.1002/anie.201409606.

(10) Neese, F. Software Update: The ORCA Program System—Version 5.0. *WIREs Comput. Mol. Sci.* **2022**, *12* (5), e1606. https://doi.org/10.1002/wcms.1606.

(11) Weigend, F.; Ahlrichs, R. Balanced Basis Sets of Split Valence, Triple Zeta Valence and Quadruple Zeta Valence Quality for H to Rn: Design and Assessment of Accuracy. *Phys. Chem. Chem. Phys.* **2005**, *7* (18), 3297–3305. https://doi.org/10.1039/B508541A.

(12) Pantazis, D. A.; Neese, F. All-Electron Basis Sets for Heavy Elements. *WIREs Comput. Mol. Sci.* **2014**, *4* (4), 363–374. https://doi.org/10.1002/wcms.1177.

(13) van Lenthe, E.; van Leeuwen, R.; Baerends, E. J.; Snijders, J. G. Relativistic regular two-component Hamiltonians. *Int. J. Quantum Chem.* **1996**, *57* (3), 281–293. https://doi.org/10.1002/(SICI)1097-461X(1996)57:3<281::AID-QUA2>3.0.CO;2-U.

(14) van Wüllen, C. Molecular Density Functional Calculations in the Regular Relativistic Approximation: Method, Application to Coinage Metal Diatomics, Hydrides, Fluorides and Chlorides, and Comparison with First-Order Relativistic Calculations. *J. Chem. Phys.* **1998**, *109* (2), 392–399. https://doi.org/10.1063/1.476576.

(15) Grimme, S.; Ehrlich, S.; Goerigk, L. Effect of the Damping Function in Dispersion Corrected Density Functional Theory. *J. Comput. Chem.* **2011**, *32* (7), 1456–1465. https://doi.org/10.1002/jcc.21759.

(16) Grimme, S.; Antony, J.; Ehrlich, S.; Krieg, H. A Consistent and Accurate Ab Initio Parametrization of Density Functional Dispersion Correction (DFT-D) for the 94 Elements H-Pu. *J. Chem. Phys.* **2010**, *132* (15), 154104. https://doi.org/10.1063/1.3382344.

(17) *Chemcraft - Graphical program for visualization of quantum chemistry computations.* https://www.chemcraftprog.com/ (accessed 2024-09-10).
